# Evaluation of genetic diversity and population structure of *Annamocarya sinensis* using SCoT markers

**DOI:** 10.1371/journal.pone.0309283

**Published:** 2024-09-04

**Authors:** Hong Pan, Libao Deng, Kaixian Zhu, Deju Shi, Feiyong Wang, Guofa Cui

**Affiliations:** 1 College of Nature Conservation, Beijing Forestry University, Beijing, China; 2 Fangcheng Golden Camellia National Nature Reserve Management Center, Fangchenggang, China; 3 Guangxi Academy of Agricultural Sciences, Nanning, China; 4 Scientific Research Academy of Guangxi Environmental Protection, Nanning, China; 5 Guangxi University, Nanning, China; Nuclear Science and Technology Research Institute, ISLAMIC REPUBLIC OF IRAN

## Abstract

*Annamocarya sinensis* (Dode) Leroy, a relict plant from the Tertiary period, is a member of *Annamocarya* genus in the Juglandaceae family. Despite its wide distribution in Guangxi Province, the habitats of this species had become fragmented and isolated, causing it facing deterioration. For protecting this endangered species, it is crucial to understand its status in the wild and genetic diversity. In this study, 216 *A*. *sinensis* accessions from 18 populations in Guangxi were examined using Start Codon Target Polymorphism (SCoT) markers for PCR amplification, genetic diversity, and population structure analysis. Out of the 20 SCoT primers used, 222 sites were amplified, with 185 being polymorphic (PPB of 83.33%). Polymorphic information content values ranged from 0.4380 to 0.4999, Nei’s genetic diversity index ranging from 0.1573 to 0.2503, and Shannon diversity index ranged from 0.1583 to 0.3812. Through AMOVA analysis, the total genetic diversity and genetic diversity within populations was calculated out as 0.3271 and 0.1542 respectively, the genetic differentiation coefficient between populations was 0.5286, with a gene flow 0.4458. Cluster analysis categorized *A*. *sinensis* germplasm into three groups, while population structure analysis divided all accessions into three ancestral sources with 19.91% showing mixed ancestral origins. No significant correlation was observed between genetic and geographical distance on the Mentel test (r = 0.07348, p = 0.7468). Overall, *A*. *sinensis* displays a relatively rich genetic diversity at the species level, albeit with a fairly uniform genetic background and high genetic differentiation. This study provides a crucial basis for the conservation and innovative use of *A*. *sinensis* germplasm resources.

## Introduction

*Annamocarya sinensis* (Dode) Leroy, a tree belonging to the genus *Annamocarya* in the Juglandaceae family, is considered a relict plant from the Tertiary Period [1–3]. However, due to challenges in reproduction and natural regeneration, the habitat requirements for the *A*. *sinensis* are quit strict. In addition, human activities have further damaged its habitats, leading to a significant decline in its natural distribution communities and individual numbers [4]. This species is currently only found in Guangxi, Guizhou, Yunnan, Hunan, and northern Vietnam [5]. Recognized as critically endangered by the World Conservation Union (IUCN), the *A*. *sinensis* is classified as a national second-level protected plant and a species with a very small population in China [6]. The wood of the *A*. *sinensis* is characterized by its light density and tough fibers, making it an ideal material for crafting and shipbuilding [7, 8]; The oil of *A*. *sinensis* seeds can be used for both edible and industrial purposes, making it a versatile oilseed plant. [9]; Its fruit peel and leaves can also be used as medicine, with good effects such as anti-bacterial, anti-inflammatory, anti-itch and pain relief [10]. Urgent measures are needed for the rescue and protection of this species. Previous research on *A*. *sinensis* has primarily focused on community surveys, photosynthetic characteristics, nutritional components, and endophytic fungi. However, there has been limited exploration of its genetic diversity and population structure. The ISSR-PCR reaction system for *A*. *sinensis* was successfully established and optimized [6], laying a crucial groundwork for subsequent studies on genetic diversity assessment and phylogenetic analysis. Genetic diversity and population structure were analyzed by using 12 pairs of EST-SSR primers on 70 *A*. *sinensis* samples from four populations [5] in a separate study, the results revealed high genetic diversity and low genetic differentiation within and between populations, and no significant correlation was found between genetic distance and geographical distance. Furthermore, the genome of chloroplast DNA of *A*. *sinensis* was sequenced [3,11], providing valuable insights into its genetic characteristics.

The Start Codon Target Polymorphism (SCoT) molecular marker is a gene-based marker that amplifies the genome by targeting the ATG translation initiation site in plant genes with conserved primers [12–14]. SCoT markers are recognized for their simplicity, efficiency, high polymorphism, and repeatability, making them extensively utilized in plant genetic diversity analysis, gene mapping, genetic linkage map construction, and germplasm identification [15–18]. There are various indicators for measuring genetic diversity. Polymorphic percentage (PPB), polymorphic information content (PIC), and resolution (Rp) are commonly used to evaluate the amplification results [19, 20].

Despite the wide distribution of *A*. *sinensis* in Guangxi, it is found that the situation of *A*. *sinensis* resources in Guangxi is worrying. Most of remaining plants are located within local sacred mountains or near shrines, existing as single or a few scattered individuals in a sporadic or island-like distribution pattern. While the local indigenous people hold deep reverence for these sacred areas and generally refrain from cutting down trees in taboo places, occasional sacrificial activities by residents may also impact local microenvironment by causing trash pollutions, vegetation clearing on the ground, et al. Meanwhile, termites and some ant species also threat *A*. *sinensis*, they rely on gnawing the heartwood inside tree trunks or the woody part of large tree roots for survival, leading to a decrease in resistance to strong winds or heavy snow, which can cause the trees to collapse and die. In addition, the seed development process of *A*. *sinensis* is quite unique, it requires a high level of humidity and a long period of germination, during this time, the demand for water is extremely high, and this period happens to coincide with the autumn and winter seasons in the subtropical regions, which are dry periods in the southern part of China, resulting in excessive water loss in the seeds, causing them to lose viability.

Although the germplasms of *A*. *sinensis* had been investigated by some DNA maker methods, there is only limited information available on its population genetic diversity and structure based on investigating relatively small number of populations [5], and no SCoT marker had been applied to the species To address this gap, we employed SCoT molecular markers to assess a larger number (216 in total) of *A*. *sinensis* germplasm samples from a variety of 18 populations in Guangxi. The aim of this study is to unveil the genetic diversity and population structure of *A*. *sinensis* and identifying if there is gene differentiation, providing essential reference data for its classification, identification, conservation (in-situ or ex-situ), and innovative utilizations.

## Material and methods

### Sample collection

Although the plant investigated in this study is an endangered species, the demand for leaves sampling is minimal thus cause no harm to intact status of *A*. *sinensis*. Additionally, the sampling locations are not situated within natural reserves, eliminating the need for permission from forestry authorities. The procedures for obtaining germplasm samples were conducted with the necessary permissions and assistances from legitimate land users, thereby avoiding any ethical or moral concerns. A total of 216 wild *A*. *sinensis* accessions were collected from 18 counties in Guangxi Province, China. In northeastern Guangxi, 52 accessions were collected, including 9 from Sanjiang County (SJ), 10 from Luzhai (LZ) in Liuzhou City, 16 from Longsheng (LS) County in Guilin City, and 17 from Jinxiu (JX) County in Laibin City. In northwest, 135 accessions were collected, including 6 from Tian’e (TE) County, 9 from Donglan (DL), 6 from Nandan (ND), 11 from Huanjiang (HJ), 14 from Luocheng (LC), 18 from Jinchengjiang (JJ), 28 from Du’an (DA), 7 from Yizhou (YZ) in Hechi City, 9 from Tianlin (TL), 14 from Leye (LY), and 13 from Lingyun (LY) in Baise City. In southwest, 24 accessions were collected, including 6 from Jingxi (JX) County and 18 from Napo (NP) in Baise City. In central Guangxi Province, 5 accessions were collected from Xincheng (XC) County in Laibin City. Detailed information of sampling coordinates were listed in Table 1. Three healthy leaves for each accession were collected from *A*. *sinensis* plants with a diameter at breast height ≥ 3.3 cm (1 Chinese inch). They were then placed in a foam box filled with dry ice, transported back to the laboratory, and stored in a -40°C refrigerator for future use.

**Table 1:** *A*. *sinensis* germplasm resources (216 accessions) and their respective localities.

Localities	Populations	Voucher Number	Longitude and latitude of localities	Genotype Code
Lingyun	LY	AS1~AS13	E 106°36′07″,N 24°20′15″	LY1~LY13
Leye	LE	AS14~AS27	E 106°35′44″,N 24°44′47″	LE1~LE14
Tianlin	TL	AS28~AS36	E 106°11′22″,N 24°26′46″	TL1~TL9
Tian’e	TE	AS37~AS42	E 107°1′15″,N 25°11′55″	TE1~TE6
Nandan	ND	AS43~AS48	E 107°38′37″, N 25°5′58″	ND1~ND6
Huanjiang	HJ	AS49~AS59	E 108°11′40″,N 25°10′10″	HJ1~HJ11
Luocheng	LC	AS60~AS73	E 108°34′25″,N 24°54′11″	LC1~LC14
Jinchengjiang	JJ	AS74~AS91	E 108°3′51″,N 24°44′45″	JJ1~JJ18
Yizhou	YZ	AS92~AS98	E 108°29′57″,N 24°37′37″	YZ1~YZ7
Donglan	DL	AS99~AS107	E 107°21′57″,N 24°22′6″	DL1~DL9
Du’an	DA	AS108~AS135	E 107°49′51″,N 24°9′46″	DA1~DA28
Sanjiang	SJ	AS136~AS144	E 109°26′38″,N 25°38′39″	SJ1~SJ9
Longsheng	LS	AS145~AS160	E 109°49′35″,N 25°37′21″	LS1~LS16
Jinxiu	JX	AS161~AS177	E 110°5′20″,N 24°3′56″	JX1~JX17
Luzhai	LZ	AS178~AS187	E 110°5′54″,N 24°37′39″	LZ1~LZ10
Napo	NP	AS188~AS205	E 105°57′16″,N 23°15′06″	NP1~NP18
JingXi	JX	AS206~AS211	E 105°57′48″,N 23°7′12″	JI1~JI6
Xincheng	XC	AS212~AS216	E 108°45′51″, N 24°5′12″	XC1~XC5

### Extraction and purification of DNA

DNA extraction from *A*. *sinensis* leaves was conducted using the modified cetyl-trimethylammonium bromide (CTAB, Beijing Solarbio Science & Technology, China) protocol [21]. The concentration and quality of DNA were assessed using a UV spectrophotometer (Thermo Scientific™ NanoDrop™ One,Germany) and 1.5% agarose gel electrophoresis (DYY-6C, Beijing Liuyi Biotechnology Co.,Ltd, China), with subsequent separation of DNA from each sample using ddH_2_O. The concentration was diluted to 30 ng/μL and stored at 4°C for later use.

### SCoT-PCR reaction

A total of 56 SCoT primers adopted by previous amplification systems [12, 22] were custom-synthesized by Sangon Biotech (Shanghai) Co., Ltd. Following the established SCoT-PCR reaction system and amplification procedures [23], four DNA samples exhibiting significant phenotypic differences were chosen for primer screening, and primers with good polymorphism, amplification efficiency, and clear bands (S1 Raw image) were selected for further experiments. The SCoT-PCR amplification reaction was carried out using a Biometra Tprofessional PCR instrument (Biometra TAdvanced 96, Germany). The reaction system comprised ddH_2_O (8 μL), 2×Taq PCR Mix (10 μL, CoWin Biosciences (Beijing), China), 30 μmol/L primer (1 μL), and 30 ng/μL template DNA (1 μL). The amplification program included pre-denaturation at 94°C for 4 min, denaturation at 94°C for 1 min, annealing at a specified temperature for 1 min, extension at 72°C for 1 min for 38 cycles, and final cryogenic storage. GelRed stain (4 μL, Beijing Solarbio Science & Technology, China) was added to the PCR product, mixed thoroughly, and subjected to 1.5% agarose electrophoresis. The results were visualized using a gel imager (BIO-RAD, USA).

### Analysis and statistics

The presence or absence of the amplified band at each corresponding site in each sample was recorded to create a binary data matrix containing ’0’ and ’1’. The polymorphism information content (PIC) and resolving power (Rp) of 216 materials were calculated using Cervus 3.0 [24]. GenAlEx (V 6.51) was utilized for AMOVA variance analysis to assess genetic differentiation [25]. Popgene32 (V 1.32) software was employed to analyze Nei’s gene diversity index (H), Shannon information index (I), and Nei’s genetic distance [26]. Ntsys pc (V 2.10) software was used for genetic similarity coefficient analysis, principal coordinate analysis (PCoA), and Mantel Test correlation analysis [27]. MEGA (V 711) software was utilized for neighbor-joining cluster analysis based on Nei’s genetic distance to generate a dendrogram [28].

Structure (V 2.3.4) software was used to analyze population structure using a Bayesian mathematical model for calculating individual genetic similarity weight values (Q value) and assessing gene flow [29]. The number of subpopulations (K-values) within the populations was determined by setting a range of values from 2 to 18. Each K-value underwent 20 repetitions,with a burn-in iteration of 100,000 followed by 10,000 Markov Chain Monte Carlo (MCMC) replications post burn-in. The results of calculations were then uploaded to the website of the Structure Harvester (http://taylor0.biology.ucla.edu/struct_harvest) and the optimal K-value for population structure analysis was determined based on the highest likelihood value (ΔK). This value indicate the most accurate estimated number of clusters for the analysis of population structure [30].

## Results

### Polymorphism and diversity analysis of amplified products

Twenty SCoT primers were carefully chosen from an initial pool of 56, showcasing a diverse array of amplified bands and significant polymorphism. These primers were then used for PCR amplification on DNA samples extracted from 216 *A*. *sinensis* germplasms. The genetic indices (Ne, Na, I, He) of selected primers are detailed in S1 Table. A total of 222 bands were observed, with 193 exhibiting polymorphism, resulting in a polymorphic band percentage (PPB) of 86.94%. On average, each primer yielded between 8 to 14 bands, with a mean of 11.1 bands per primer. The amplified bands varied in length from 200 to 2200 bp, primarily falling within 300 to 1700 bp. The average Polymorphism Information Content (PIC) value for the 20 primers was 0.481, with individual values ranging from 0.4380 to 0.4999, indicating a high degree of polymorphism. Additionally, the average Resolving Power (Rp) was calculated at 9.197 (ranging from 6.59 to 12.90), underscoring the significant discriminatory capability of these primers (Table 2). The results suggest that these selected primers possess good polymorphic potential and high resolution [31], meeting the necessary experimental criteria.

**Table 2:** Comparison of amplification effects of 20 SCoT primers.

Primer	Primer sequence	FS (bp)	TNB	PNB	PPB (%)	PIC	Rp	AT (°C)
SCoT 6	CAACAATGGCTACCACGC	300–1500	12	10	83.33	0.4332	9.18	56
SCoT 11	AAGCAATGGCTACCACCA	200–1000	10	8	80	0.4992	8.1	54
SCoT 12	ACGACATGGCGACCAACG	400–1700	8	7	87.5	0.4975	6.59	58
SCoT 14	ACGACATGGCGACCACGC	300–2000	10	9	90	0.4936	7.4	60
SCoT 17	ACCATGGCTACCACCGAG	300–1500	10	9	90	0.4795	7.63	58
SCoT 18	ACCATGGCTACCACCGCC	300–2000	12	11	91.67	0.4955	10.57	60
SCoT 19	ACCATGGCTACCACCGGC	450–1600	11	9	81.82	0.497	9.15	60
SCoT 20	ACCATGGCTACCACCGCG	500–2000	12	10	83.33	0.4348	10.16	60
SCoT 21	ACGACATGGCGACCCACA	350–2200	13	11	84.62	0.4659	11.31	58
SCoT 23	CACCATGGCTACCACCAG	400–2200	13	12	92.31	0.4631	10.19	58
SCoT 24	CACCATGGCTACCACCAT	300–1500	9	8	88.89	0.4834	7.08	56
SCoT 31	CCATGGCTACCACCGCCT	350–1500	12	11	91.67	0.4906	9.82	60
SCoT 32	CCATGGCTACCACCGCAC	450–1500	11	10	90.91	0.4829	8.86	60
SCoT 34	ACCATGGCTACCACCGCA	400–1400	11	10	90.91	0.4999	8.43	58
SCoT 35	CATGGCTACCACCGGCCC	300–1800	14	12	85.71	0.478	12.9	62
SCoT 36	GCAACAATGGCTACCACC	300–1800	13	11	84.62	0.438	11.5	56
SCoT 38	CAATGGCTACCACTAACG	350–1200	10	8	80	0.4964	8.38	54
SCoT 40	CAATGGCTACCACTACAG	250–1100	9	7	77.78	0.4804	8.18	54
SCoT 43	CAATGGCTACCACCGCAG	250–1500	11	10	90.912	0.4991	9.89	58
SCoT 46	ACAATGGCTACCACTGCC	350–1800	11	10	90.91	0.4999	8.61	54
Mean		-	11.1	9.65	86.84	0.4804	9.2	
Total		200–2200	222	193	86.94-	-	-	

Note: FS, fragment size; TNB, total number of bands; NPB, number of polymorphic bands; PPB, polymorphic band percentage; PIC, polymorphism information content; Rp, resolving power; AT, annealing temperature

The observed alleles (Na) in various populations range from 1.2613 to 1.8108, with an average of 1.4667. Effective alleles (Ne) range from 1.1866 to 1.4207, averaging at 1.2626. The Shannon diversity index (I) ranges between 0.1583 and 0.3812, with an average of 0.2325. Nei’s genetic diversity index (H) varies from 0.1072 to 0.2503, averaging at 0.1542. The Jinxiu population (JX) exhibits the highest genetic polymorphism (81.08%), while the Nandan population (ND) shows the lowest genetic diversity index (26.13%). By excluding the Jinxiu population (JX), genetic diversity index values in other populations were below 60%. The ranking of populations based on genetic diversity, from highest to lowest, were as follows: Jinxiu (JX) > Luzhai (LZ) > Leye (LE) > Napo (NP) > Luocheng (LC) > Longsheng (LS) > Jinchengjiang (JJ) > Du’an (DA) > Xincheng (XC) > Lingyun (LY) > Sanjiang (SJ) > Jingxi (JI) > Yizhou (YZ) > Huanjiang (HJ) > Tian’e (TE) > Tianlin (TL) > Donglan (DL) > Nandan (ND) (Table 3).

**Table 3:** Genetic diversity levels of 18 populations of *A*. *sinensis*.

Population	PPB %	N_a_	N_e_	I	H
LY	48.20	1.482	1.2702	0.2382	0.158
LE	56.70	1.5676	1.3016	0.2738	0.1801
TL	30.10	1.3018	1.1866	0.1617	0.1086
TE	34.23	1.3423	1.2067	0.1813	0.1211
ND	26.13	1.2613	1.2011	0.1596	0.1107
HJ	36.49	1.3649	1.2224	0.1923	0.129
LC	55.86	1.5586	1.3122	0.2725	0.181
JJ	52.25	1.5225	1.2842	0.2531	0.1674
YZ	37.39	1.3739	1.2508	0.2085	0.1416
DL	28.83	1.2883	1.1874	0.1583	0.1072
DA	50.45	1.5045	1.2674	0.2386	0.1573
SJ	45.95	1.4595	1.237	0.2183	0.1425
LS	52.70	1.527	1.2598	0.2387	0.1558
JX	81.08	1.8108	1.4207	0.3812	0.2503
LZ	59.46	1.5946	1.331	0.294	0.1944
NP	55.86	1.5586	1.2488	0.2426	0.1549
JI	38.74	1.3874	1.2373	0.2076	0.1389
XC	49.55	1.4955	1.3024	0.2647	0.1769
Mean	46.67	1.4667	1.2626	0.2325	0.1542
Species	86.94	1.9955	1.5462	0.491	0.3243

Note: PPB, Percentage of Polymorphism Na, Observed number of alleles; Ne, Effective number of alleles; H, Nei’s gene diversity; I, Shannon’s Information index.

### Neighbor-joining clustering

**Fig 1:**
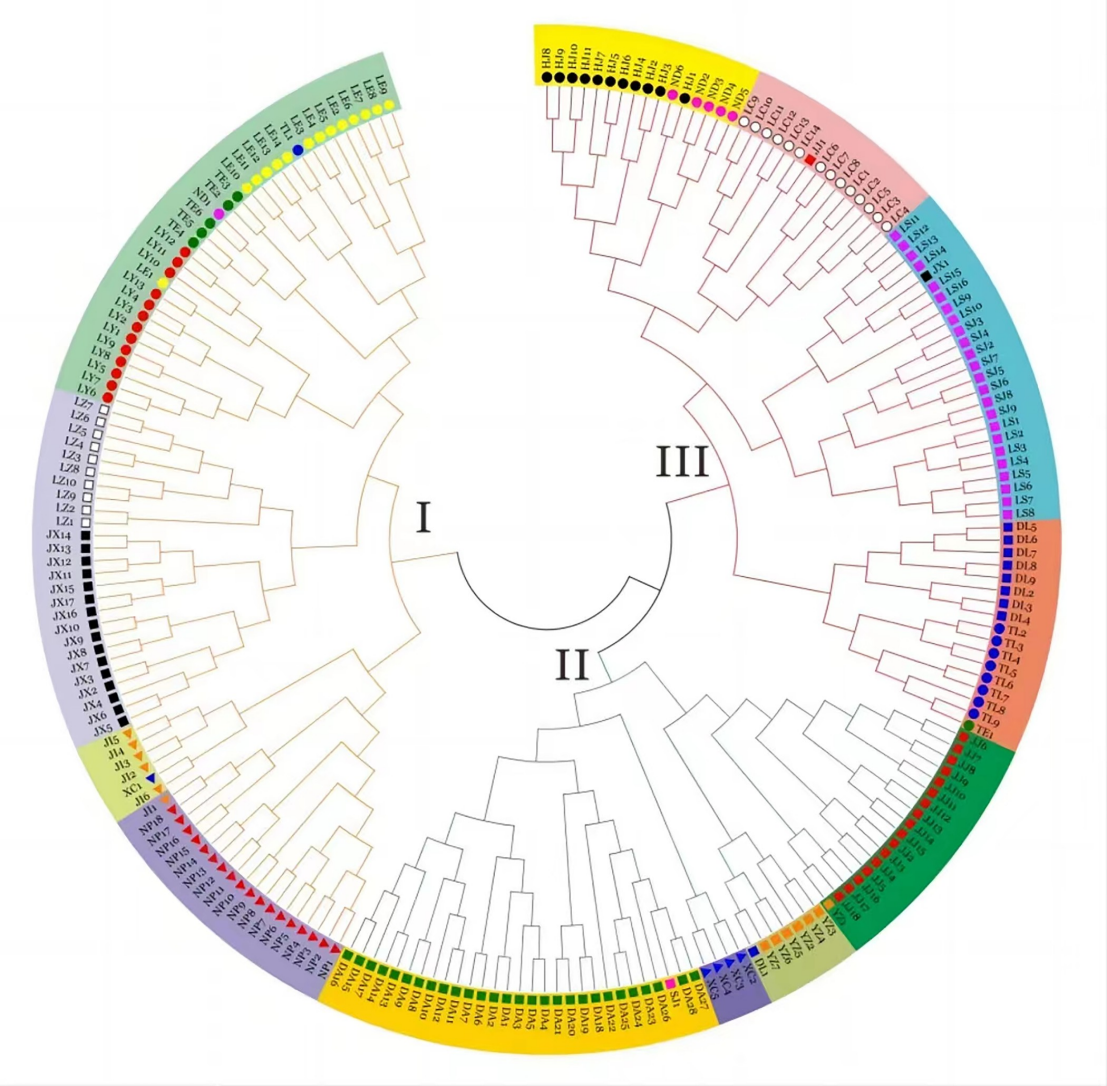
Neighbor-Joining cluster diagram of 216 *A*. *sinensis* samples.

Neighbor-Joining cluster analysis results indicate that at a Nei’s genetic distance threshold of 0.342, 216 *A*. *sinensis* samples can be classified into 3 main categories. Category I comprises individuals from Lingyun (LY), Leye (LE), Jinxiu (JX), Luzhai (LZ), Jingxi (JI), and Napo (NP) populations, along with some individuals from Tianlin (TL1), Nandan (ND1), and Xincheng (XC1). Category II includes individuals from Duan (DA), Xincheng (XC), Yizhou (YZ), and Jinchengjiang (JJ) populations. Category III encompasses individuals from Tianlin (TL), Donglan (DL), Longsheng (LS), Sanjiang (S), Luocheng (LC), Nandan (ND), and Huanjiang (HJ) populations, as well as individuals from Tian’e (TE, 1), Jinxiu (JX, 1), and Jinchengjiang (JJ1). When the genetic distance threshold is set at 0.281, the *A*. *sinensis* samples are further divided into 12 subgroups. Subgroup I-I includes individuals from Lingyun (LY), Leye (LE), Tian’e (TE), and some individuals from Tianlin (TL1). Subgroup I-II comprises samples from Luzhai (LZ) and Jinxiu (XJ) populations. Subgroup I-III includes individuals from Jingxi (JI) and Xincheng (XC1) populations. Subgroup I-IV consists of samples from Napo (NP) and Jingxi (JI1). Subgroup II-I only includes individuals from Duan (DA) population. Subgroup II-II comprises samples from Xincheng (XC) population. Subgroup II-III includes individuals from Yizhou (YZ) and Tianlin (TL1) populations. Subgroup II-IV consists of individuals from Jinchengjiang (JJ) and Yizhou (YZ1) populations. Subgroup III-I includes individuals from Tianlin (TL), Donglan (DL), and Tian’e (TE1) populations. Subgroup III-II comprises individuals from Longsheng (LS), Sanjiang (SJ), and Jinxiu (JX1) populations. Subgroup III-III includes individuals from Luocheng (LC) and Jinchengjiang (JJ1) populations. Subgroup III-IV consists of samples from Huanjiang (HJ) and Nandan (ND) populations (Fig 1). The division results indicated that most genotypes collected from the same location tended to cluster together, indicating that the tested *A*. *sinensis* samples share similar genetic backgrounds. The Mantel test analysis revealed no significant correlation between genetic distance and geographical distance (S2 Table, S1 Fig) of *A*. *sinensis* (r = 0.07348, p = 0.7468). This finding also explains why certain populations that are geographically close to each other were not clustered accordingly in the Neighbor-Joining clustering analysis.

### PCoA analysis

**Fig 2:**
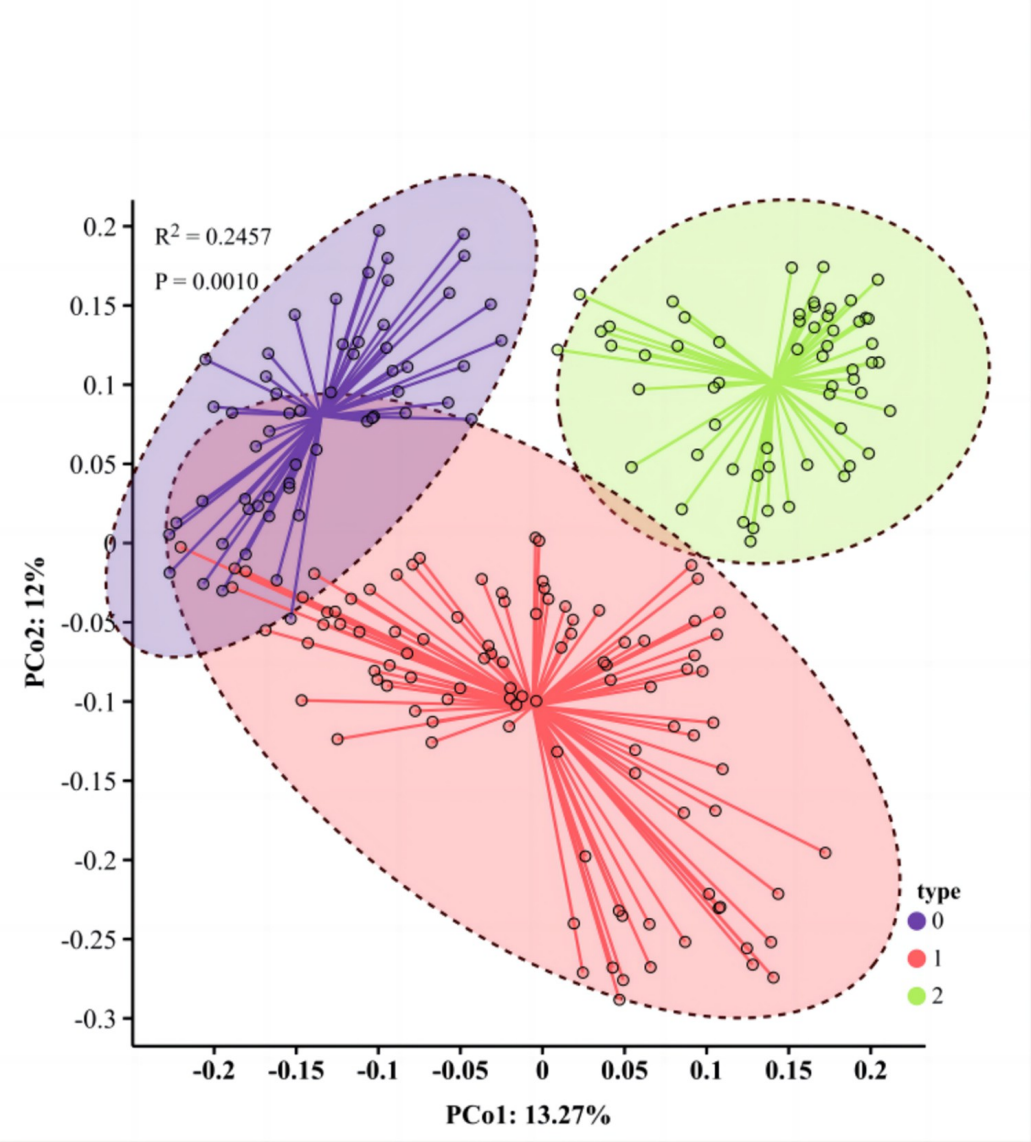
Analysis of dimensionality reduction of PCoA in *A*. *sinensis*.

PCoA was performed to validate the clustering results obtained. By assessing the spatial projection area ratio of different materials, the consistency of variation among groups was assessed based on the contribution rate of each component. The PCoA results of 216 *A*. *sinensis* samples revealed that the first three principal components accounted for 10.17%, 8.73%, and 5.78% of the variation, respectively, with a cumulative contribution rate of 24.68%. This cumulative rate effectively captured the main information from the original data. The three-dimensional distribution map showed a clear division of the tested materials into three main categories. Category 1 included Lingyun (LY), Leye (LE), Napo (NP), and Jingxi (JI); Category 2 comprised Longsheng (LS), Donglan (DL), Tianlin (TL), Huanjiang (HJ), Sanjiang (SJ), Luocheng (LC), Nandan (ND), Jinxiu (JX), and Luzhai (LZ); and Category 3 consisted of Duan (DA), Jinchengjiang (JJ), Xincheng (XC), and Yizhou (YZ) (Fig 2). The PCoA clustering results for *A*. *sinensis* samples were consistent with those obtained from Neighbor-Joining cluster analysis.

### Population structure analysis

**Fig 3:**
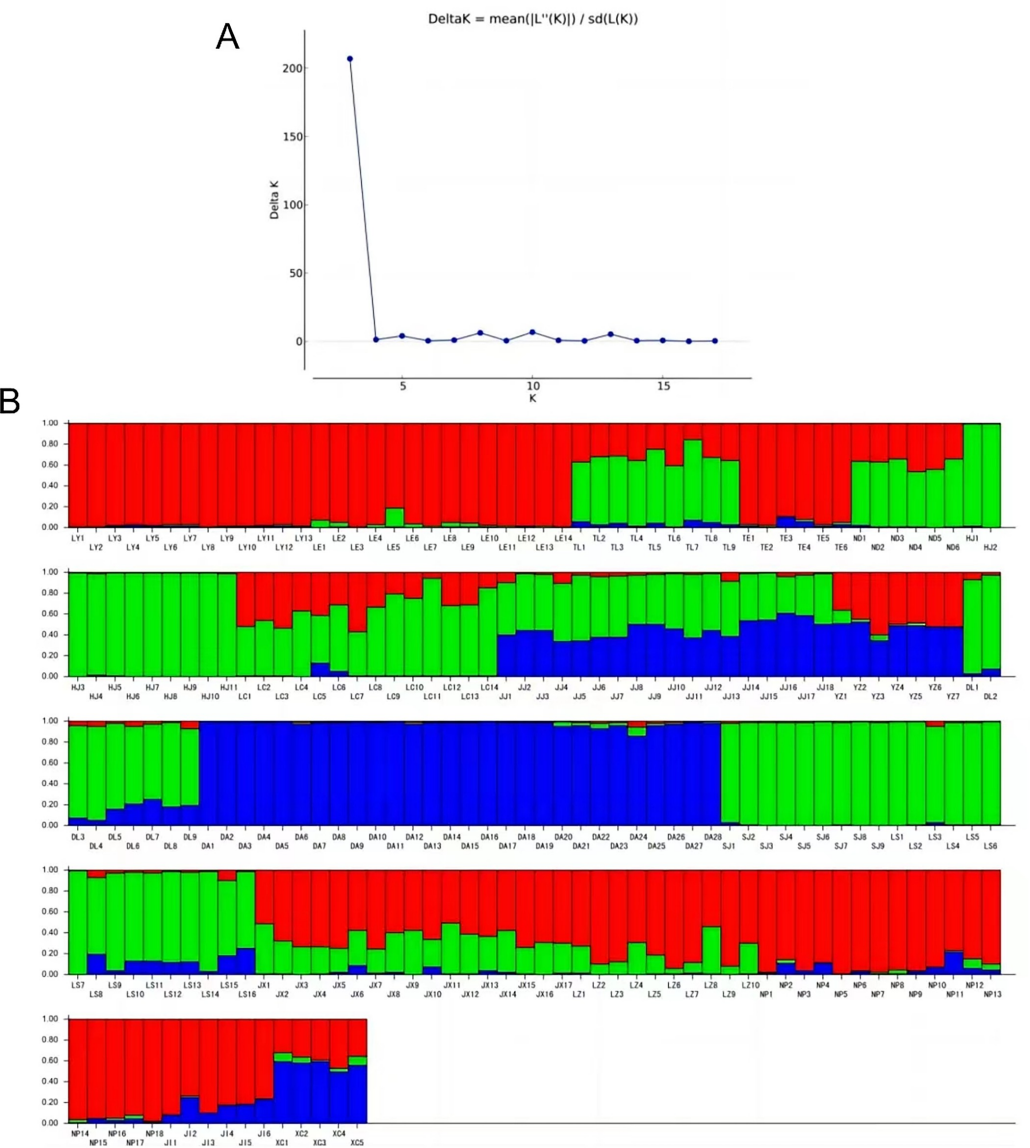
**(A)**K value change rate graph; **(B)** Population genetic structure analysis of 216 *A*. *sinensis*.

Population structure analysis was conducted using Structure software to analyze the test materials, with the ΔK value used to determine the optimal K value [30]. The peak value (Fig 3A) indicated the optimal population number of *A*. *sinensis* (K = 3), suggesting that the test materials could be divided into three major groups (Fig 3B). Group 1 comprised samples with red patches, including most of Lingyun (LY), Leye (LE), Tian’e (TE), Luzhai (LZ), Napo (NP), Jingxi (JX), and Jinxiu (JX); Group 2 included samples with green patches, such as Tianlin (TL), Nandan (ND), Huanjiang (HJ), Donglan (DL), Sanjiang (SJ), Longsheng (LS), and Luochengda, along with some samples from Jinchengjiang (JJ); Group 3 consisted of samples with blue patches, including those from Duan (DA), Xincheng (XC), and some from Jinchengjiang (JJ) and Yizhou (YZ). Geographical origin comparisons revealed that populations within the same group were not entirely clustered based on geographical distance, with some samples remaining unassigned to a group due to unknown mixed color patches. Different colors represented distinct ancestral sources with their content (expressed by Q value) indicating proximity to a specific ancestor. Out of the 216 accessions analyzed, 173 (80.08%) had Q values ≥ 0.6 and were categorized into three groups, suggesting relatively simple genetic structures. Cluster 1 comprised 78 samples, cluster 2 had 66 samples, and cluster 3 included 29 samples. The remaining 43 samples (19.91%) were provisionally classified as mixed groups due to unknown mixed components (Q value < 0.6) (Fig 3B, S3 Table).

Analyzing the remaining mixed taxa, 14 materials (TL1, TL6, ND4, ND5, LC1, LC3, LC5, LC7, JX6, JX8, JX9, JX11, JX14, and LZ8) exhibit similar characteristics. Their genetic composition is mainly derived from ancestors Q1 and Q2. While some samples in the JJ population have a single genetic background, the majority display mixed genetic origins, predominantly influenced by ancestors Q2 and Q3. The YZ population and the population of XC (excluding XC1) all have a mixed genetic background of Q1 and Q3. These 43 materials with mixed ancestral origins suggest historical gene exchange or gene penetration events between their populations, resulting in complex genetic backgrounds that are remarkably similar.

### AMOVA analysis

AMOVA variance analysis was conducted using POPGEN32 software to assess the genetic variation among the samples. The total genetic diversity (Ht) was calculated as 0.3271, with genetic diversity within populations (Hs) at 0.1542, and the genetic differentiation coefficient (Gst) between populations as 0.5286. The results indicate that 52.86% of the total genetic diversity variation arises from inter-population differences, while 47.14% comes from intra-population differentiation. Further AMOVA analysis was performed using GenAlEx 6.51b2 software on the test samples, confirming these findings. The variance test results (Table 4) showed that 51.0% of the genetic variation originated from differences between populations, and 49.0% from within populations, both statistically significant. These AMOVA results closely align with the analysis from the POPGEN software, suggesting that genetic differentiation in *A*. *sinensis* is influenced by factors both within and outside the population. The gene flow (Nm) was calculated as 0.4458.

**Table 4:** AMOVA analysis within and among populations of *A*. *sinensis*.

Source of variance	df	SSD	MSD	Variance component	Percentage (%)	P value
Among populations	17	4543.401	267.259	20.872	51	< 0.001**
Within populations	198	3991.247	20.158	20.158	49	< 0.001**
Total	215	8534.648		41.03		

Note: df,degree of freedom; SSD, Sum of Squared Deviations; MSD, Mean of Squared Deviations

## Discussion

Evolutionary biologists share common interest in understanding patterns related to rare species. Their goal is to investigate whether these species exhibit decreased genetic diversity or restricted gene flow among populations, as expected by population genetic theory for small, isolated populations [32]. Our study focuses on analyzing genetic diversity distribution and population structure of *A*. *sinensis* to provide valuable insights for developing effective conservation strategies, particularly when prioritizing specific populations for protection. In situations of low population structure, the lose an individual population has minimum impact on the genetic diversity of the entire species. However, in cases of high population structure, the loss of a single population can significantly decrease overall genetic variation.

### Genetic diversity and differentiation of *A*. *sinensis* in Guangxi

Genetic diversity and differentiation characteristics of *A*. *sinensis* are crucial for the stability, continuity, and development of this species. A decrease in genetic diversity can hinder the species’ ability to adapt to its environment [17, 33, 34]. The scattered distribution of *A*. *sinensis* resources leads to reproductive isolation, limiting gene exchange and decreasing population genetic diversity. Given the delicate ecological niche of *A*. *sinensis*, investigating its genetic diversity is vital. Parameters such as PPB, PIC, and Rp are commonly used to evaluate polymorphism levels. A species is considered to have high polymorphism when PIC > 0.5, medium polymorphism when 0.25 < PIC ≤ 0.5, and low polymorphism when PIC ≤ 0.25. The study found a PPB range of 72.73% to 92.31%, with PIC and Rp values ranging from 0.4380 to 0.4999 and 6.59 to 12.90, respectively. The selected primers demonstrated moderate to high polymorphism and amplification resolution, meeting the necessary criteria for subsequent tests.

The analysis of genetic diversity in *A*. *sinensis* revealed that the average diversity parameter values within populations (Na = 1.4667, Ne = 1.2626, H = 0.1542, I = 0.2325) were notably lower than the genetic diversity parameter values at species level (Na = 1.9955, Ne = 1.5462, H = 0.3243, I = 0.4910). Further investigation highlighted that, except for the Jinxiu (JX) population exhibiting relatively high genetic diversity parameter values (Na = 1.8108, Ne = 1.4207, I = 0.3812, H = 0.2503), the other populations exhibited lower diversity parameter values, suggesting significant barriers to genetic exchange between populations and resulting in a low mutation rate at gene sites. This observation may be associated with population size and reproductive strategies. Subsequent research found that the Jinxiu (JX) population consists of 31 *A*. *sinensis* trees, with 7 being mature trees with a diameter at breast height of ≥ 60 cm, while most individuals are saplings or nearly mature trees with diameters at breast height ranging from 3.3 to 30 cm. Population structure analysis also confirmed a diverse genetic background with ancestors originating from various sources.

Most species of walnut display a hybridity and morphological characteristics such as wind-pollinated flowers, separated male and female inflorescences, and dichogamy [35–37],*A*. *sinensis* has the same characteristics [38]. Only 6.2% of seeds from a single *A*. *sinensis* plant were found germinating normally within a few kilometers [5],indicating that *A*. *sinensis*, like many walnut varieties, demonstrates hybrid vigor, a trait indirectly supported by previous studies [35, 36, 39]. Extensive phenological observations revealed that *A*. *sinensis* is a monoecious and dioecious plant, with male flowers located in the middle of fruiting branches and female flowers at the top, creating spatially separated pollination pathways. This spatial arrangement may enhance receptivity to cross-pollination, leading to diverse variations and differentiation from pollen of the same genus. While it remains uncertain if self-pollination exists among *A*. *sinensis*, however, mature *A*. *sinensis* plants in wild were observed flowered and yield fruits even in the absence of conspecifics within several kilometers. This observation suggests the potential evidence for the presence of apomixis in *A*. *sinensis*,which could account for the low genetic diversity observed in some populations. Previous study suggested that pecan exhibits apomictic reproduction, developing embryos directly from nucellar cells without fertilization [40]. Given the close relation of the genus *Carya cathayensis* to *A*. *sinensis*, it is possible that a similar phenomenon occurs in *A*. *sinensis* during seed formation, akin to *Carya cathayensis*. This insight sheds light on why populations like Huanjiang (HJ) possess a relatively large number of individuals (30 strains) but low polymorphism or diversity. Diverging from previous results that AMOVA findings consistently demonstrated genetic differentiation within and among the population in four *A*. *sinensis* populations, which highlighted that 87% of the genetic variation was within the population, while 13% was between populations [5]. The discrepancy in results could be attributed to the use of different molecular markers and variations in sampling locations. Despite a broader sampling range, the number of samples was lower compared to this study. Wang et al. utilized EST-SSR markers to analyze 134 accessions of the endangered *Juglans mandshurica* Maxim germplasm [41], finding a high level of polymorphism within the population (PIC = 0.507) coupled with low genetic diversity (H_O_ = 0.333, I = 0.705). They observed low genetic differentiation between populations (11%) and high gene flow (Fst = 0.103, Nm = 2.894), potentially influenced by pollen length. Xiao et al. investigated the genetic diversity of 128 accessions of the endangered *Michelia crassipe* using 14 SSR primers [42], reporting a moderate level of genetic diversity (He = 0.536, I = 1.121) and low genetic differentiation (Fst = 0.108). Kumar et al. studied 36 accessions of the endangered *Aconitum heterophyllum* using SSR primers [43], They reported low genetic diversity within populations (I = 0.246, He = 0.194) and high genetic variation between populations (92%), with low variation within populations (8%). A correlation between geographical distance and genetic distance was observed, likely attributed to limited genetic diversity, high genetic differentiation, and low gene flow resulting from population fragmentation. The Fixation Index (Fst) is used to estimate genetic differentiation between populations, the value of Fst above 0.25 indicate significantly great differentiation [44]. However, since Fst is based on biallelic markers, gene differentiation coefficient (Gst)—a conceptual similar estimator is used for evaluating genetic differentiation between populations for multiallelic markers [45]. In this study, the Gst among populations was 0.5286, indicating very significant differentiation between populations.

The gene flow observed in this study (Nm = 0.4458) indicated a low level of gene flow between *A*. *sinensis* populations. This could be associated with habitat fragmentation or the distinct pollination pathway of *A*. *sinensis*. When Nm < 1, genetic shift could play a key role in gene differentiation within populations [46]. The index of genetic diversity (PPB, Ne, Na, I, He) of populations and species level were relatively consistent with information reveled by results of Gst and Nm, showing genetic differentiation between populations is significant while gene flow is poor, the populations are facing deterioration due to potential declining genetic diversity.

### Cluster analysis, PCoA analysis, and population structure analysis

The Neighbor-Joining clustering results, based on Nei’s genetic distance, identified genetic distance thresholds of 0.342 and 0.281, leading to the classification of 3 major categories and 12 minor categories. PCoA analysis demonstrated that the *A*. *sinensis* samples could be classified into 3 categrories, accounting for 24.68% of the total variation. The optimal K value determined in the population structure analysis was 3, indicating the presence of three major groups among the tested materials. These analyses yielded results that were largely consistent with a previous study [5], highlighting the sensitivity and diversity of the selected SCoT marker. The Mantel test correlation indicated a weak association between genetic and geographical distances (r = 0.07348, p = 0.7468), consistent with prior research on *A*. *sinensis* and related walnut species [47], suggesting that genetic differentiation in *A*. *sinensis* populations may not be solely influenced by geographical distance but also by environmental adaptability. Notably, Neighbor-Joining clustering consistently showed that several individual samples clustered with geographically distant groups rather than those from the same population origin, suggesting closer relationships. A similar pattern was observed in the population structure analysis, where 19.91% of individual samples displayed mixed ancestry, suggesting past gene exchange events. The partitioning in the Neighbor-Joining cluster analysis aligns with the STRUCTURE cluster analysis results, indicating some gene introgression between populations. The role of environmental factors or human activities in species evolution should also be considered: since *A*. *sinensis* tend to grow near water and have relatively large heavy seeds, it is possible some seeds were washed downstream by water flow to other habitats; It is also possible that seeds were purposefully carried by humans to distant habitats, particularly germplasms near sacred shrine forests, possibly transported and planted as talismans. The presence of these unique individuals could play an important role in maintaining health genetic diversities in specific populations and preventing their deterioration.

### The current state of *A*. *sinensis* and recommendations for protection

Recent surveys and studies have shown that *A*. *sinensis* in Guangxi are facing severe habitat fragmentation. It is estimated that there are fewer than 400 *A*. *sinensis* trees remaining in the wild, with less than 200 mature trees having a diameter at breast height of ≥ 30 cm. These trees are predominantly located in sacred mountains and shrine forests, often found in the peaks and depressions of lava landforms. Despite being protected, the species is at risk due to its specific habitat requirements, limited breeding ability, increasing human interference, and rising threats from pests and diseases. The tree age composition of most populations has not been updated for a long time, indicating a concerning lack of regeneration. The species is predicted to face a high risk of extinction in the future.

It is crucial for focusing on the conservation of this endangered tree species. This can be achieved through in-situ and ex-situ protection measures aimed to increase the population and genetic diversity of *A*. *sinensis*, ensuring their continued survival and development. Additionally, investing in scientific research to understand the primary causes of endangerment, evolutionary trends, and implementing modern biotechnology for efficient regeneration and seed bank preservation is essential. By expanding the application of *A*. *sinensis* in agriculture and forestry while promoting their conservation, a balance between utilization and protection can be achieved. Furthermore, efforts should be made to reduce human interference and encourage natural population regeneration.

It is found that fragmentation or isolation of *A*. *sinensis* populations hinders gene flow, reducing the frequency of pollination and genetic exchange among populations, leading to inbreeding, and reducing seed viability, ultimately resulting in a deterioration of genetic diversity. The results of this study suggest the following conservation strategies: In-situ conservation should prioritize populations with high genetic diversities (such as JX population in this study); individual plant of unique genotypes (identified by Neighbor-Joining and population structure analysis) should also be prioritized for protection and considered for ex-situ transplanting. Through genetic diversity investigations, information on core germplasm resources of *A*. *sinensis* should be collected for screening and breeding, the clustering analysis can significantly reduce the redundancy in constructing core germplasm.

## Conclusion

It is imperative to focus on the conservation of the endangered tree species *A*. *sinensis*. This can be achieved through both in-situ and ex-situ protection measures aimed at increasing the population and genetic diversity of *A*. *sinensis* to ensure their continued survival and development. Investing in scientific research to understand the primary causes of endangerment, evolutionary trends, and implementing modern biotechnology for efficient regeneration and seed bank preservation is essential. By expanding the application of *A*. *sinensis* in agriculture and forestry while promoting their conservation, a balance between utilization and protection can be achieved. Efforts should also be made to reduce human interference and encourage natural population regeneration. Fragmentation or isolation of *A*. *sinensis* populations has been found to hinder gene flow, reducing the frequency of pollination and genetic exchange among populations, leading to inbreeding and reduced seed viability, ultimately resulting in a deterioration of genetic diversity. The results of this study suggest the following conservation strategies: In-situ conservation should prioritize populations with high genetic diversities, such as the JX population in this study. Individual plants with unique genotypes, identified through Neighbor-Joining and population structure analysis, should also be prioritized for protection, and considered for ex-situ transplanting. Through genetic diversity investigations, information on core germplasm resources of *A*. *sinensis* should be collected for screening and breeding, and clustering analysis can significantly reduce redundancy in constructing core germplasm.

## Supporting information

S1 Raw image (PDF)

S1 Table Genetic diversity index of 20 primers. (PDF)

S2 Table Genetic distance and geographical distances between populations in Guangxi. (PDF)

S3 Table Q value compositions of 216 A. sinensis accessions. (PDF)

S1 Fig Correlation analysis of mantel test between genetic distance and geographical distance. (PDF)

S1 Dataset (ZIP)
