# Supplementary material for: Evaluation of genetic diversity and population structure of Annamocarya sinensis using SCoT markers
Source: PLoS One. 2024 Sep 4;19(9):e0309283. doi: 10.1371/journal.pone.0309283 (PMC11373820; doi:10.1371/journal.pone.0309283)
Supplement: S1 Raw image — (PDF) [file pone.0309283.s001.pdf]

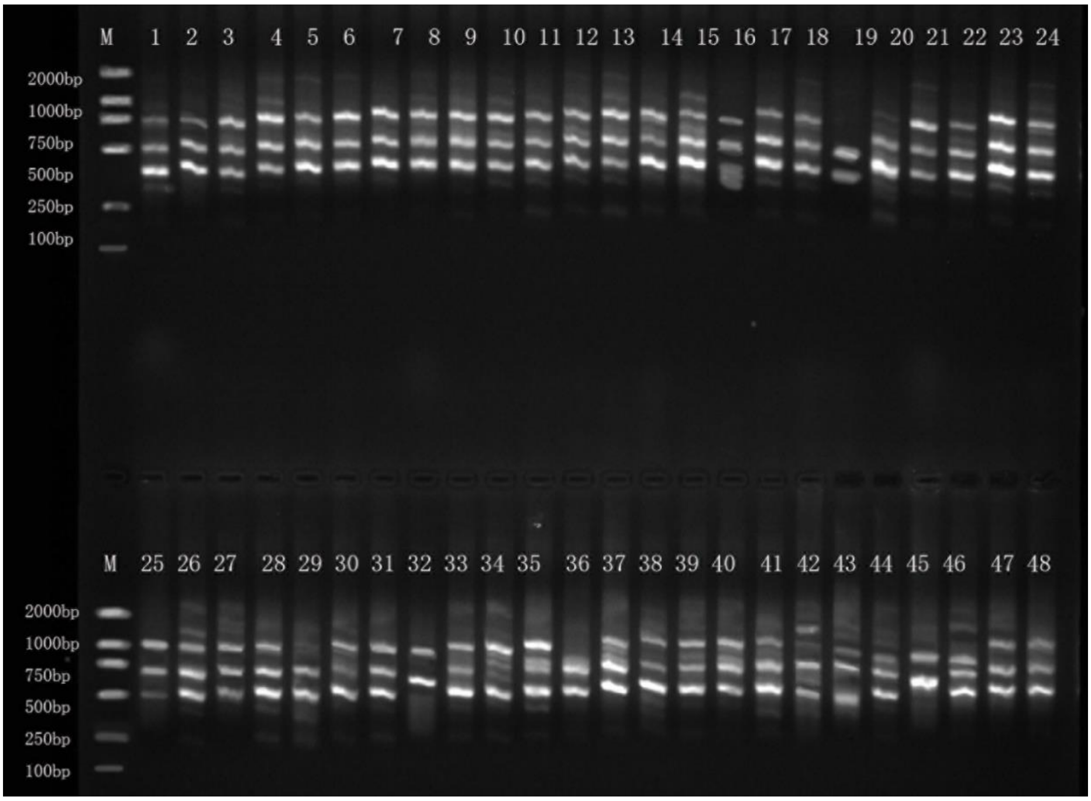

1. Amplification effect of primer ScoT 6 on *A.sinesis* samples (Voucher No. AS 1-48)

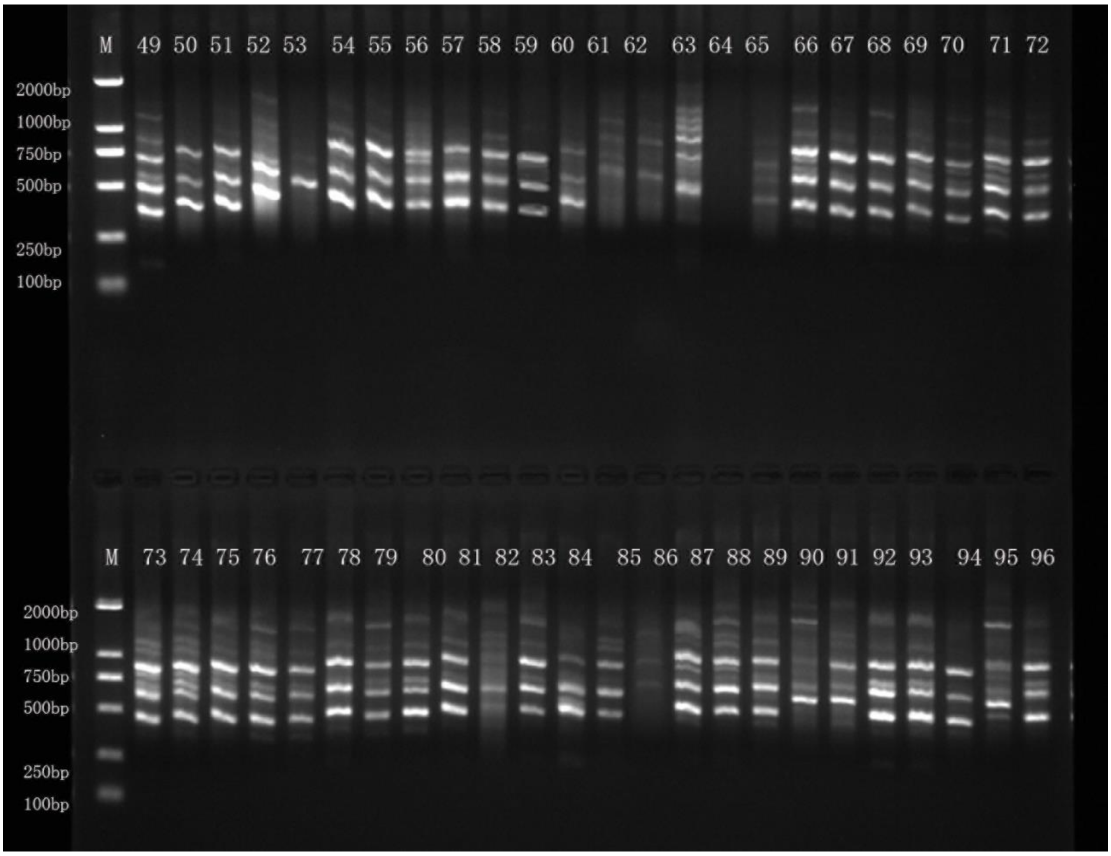

2. Amplification effect of primer ScoT 6 on *A.sinesis* samples (Voucher No. AS 49-96)

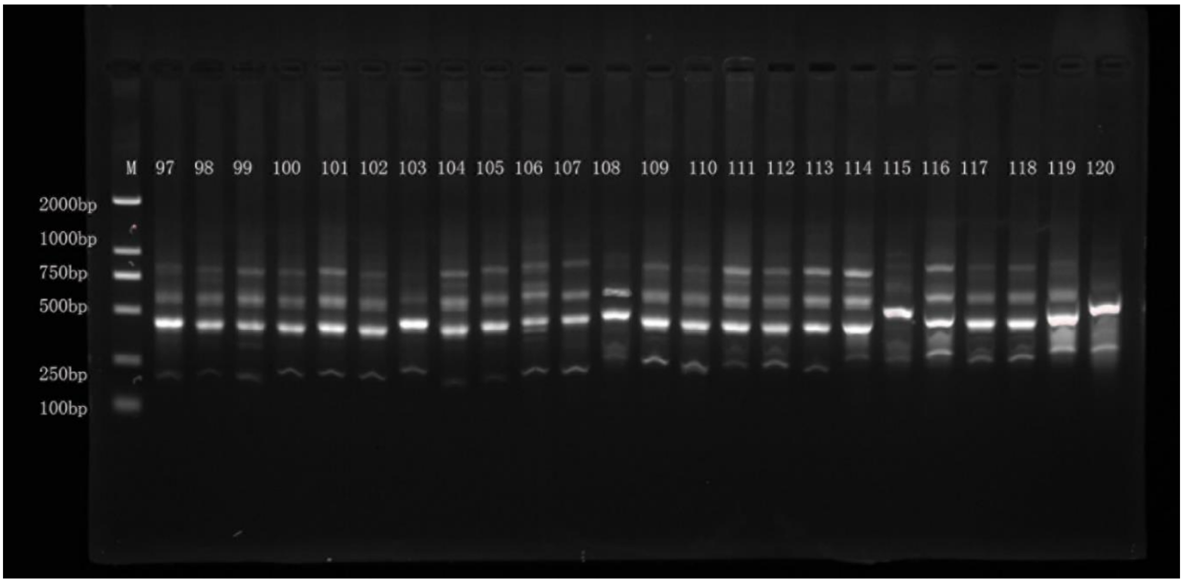

3. Amplification effect of primer ScoT 6 on *A.sinesis* samples (Voucher No. AS 97-120)

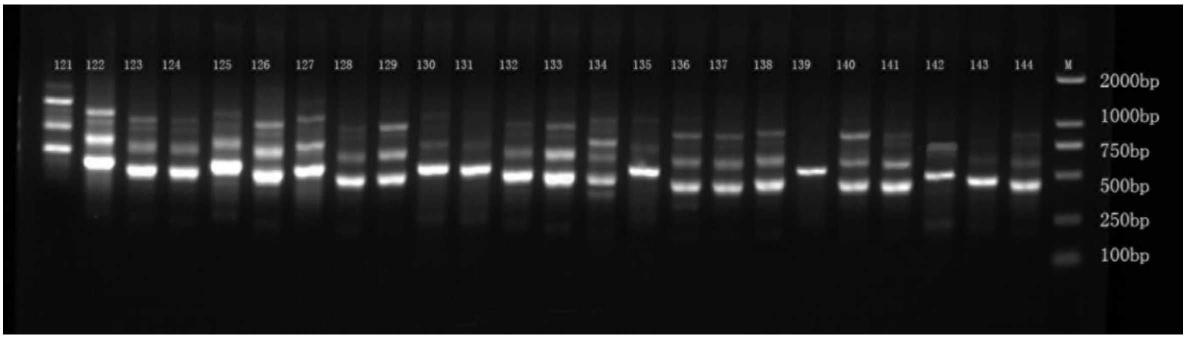

4. Amplification effect of primer ScoT 6 on *A.sinesis* samples (Voucher No. AS 121-144)

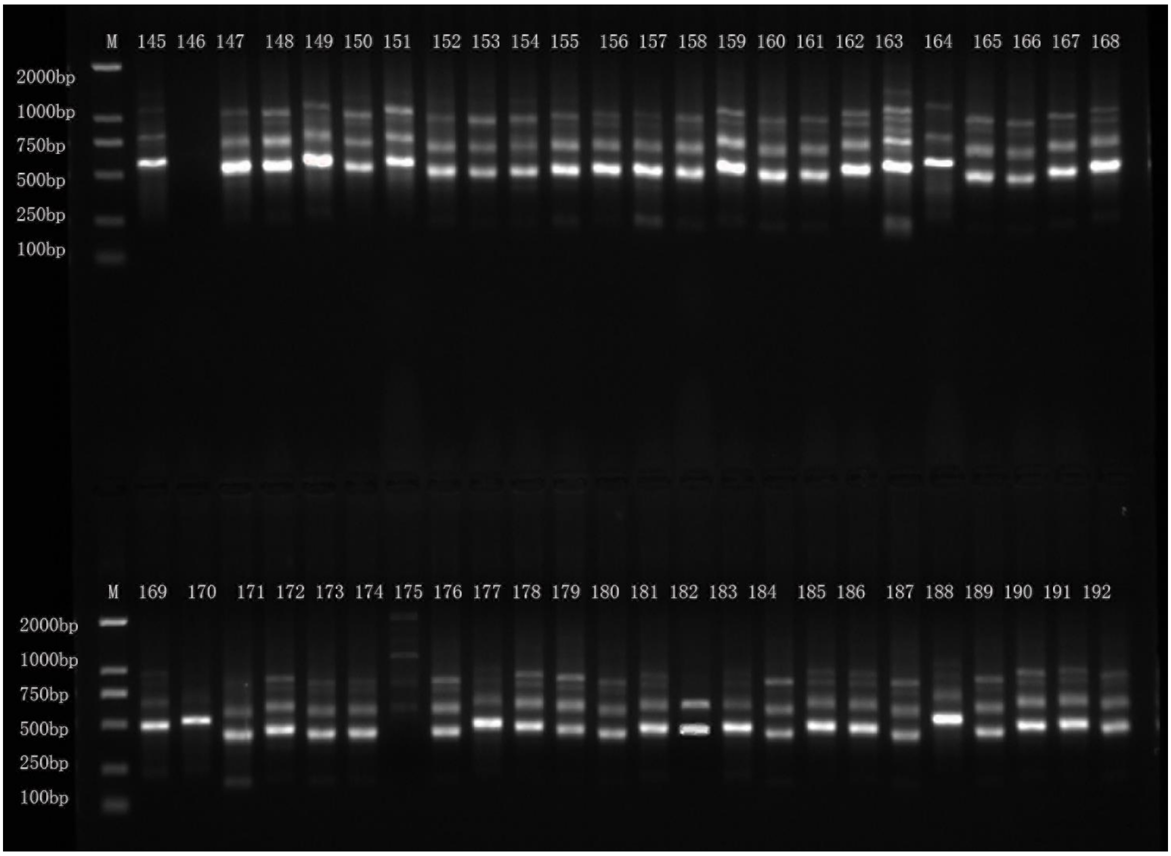

5. Amplification effect of primer ScoT 6 on *A.sinesis* samples (Voucher No. AS 145-192)

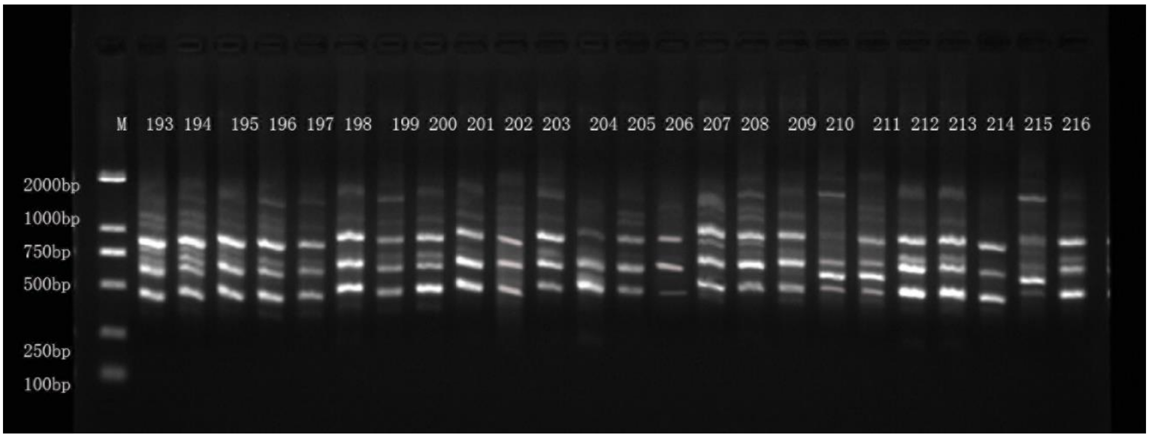

6. Amplification effect of primer ScoT 6 on *A.sinesis* samples (Voucher No. AS 193-216)

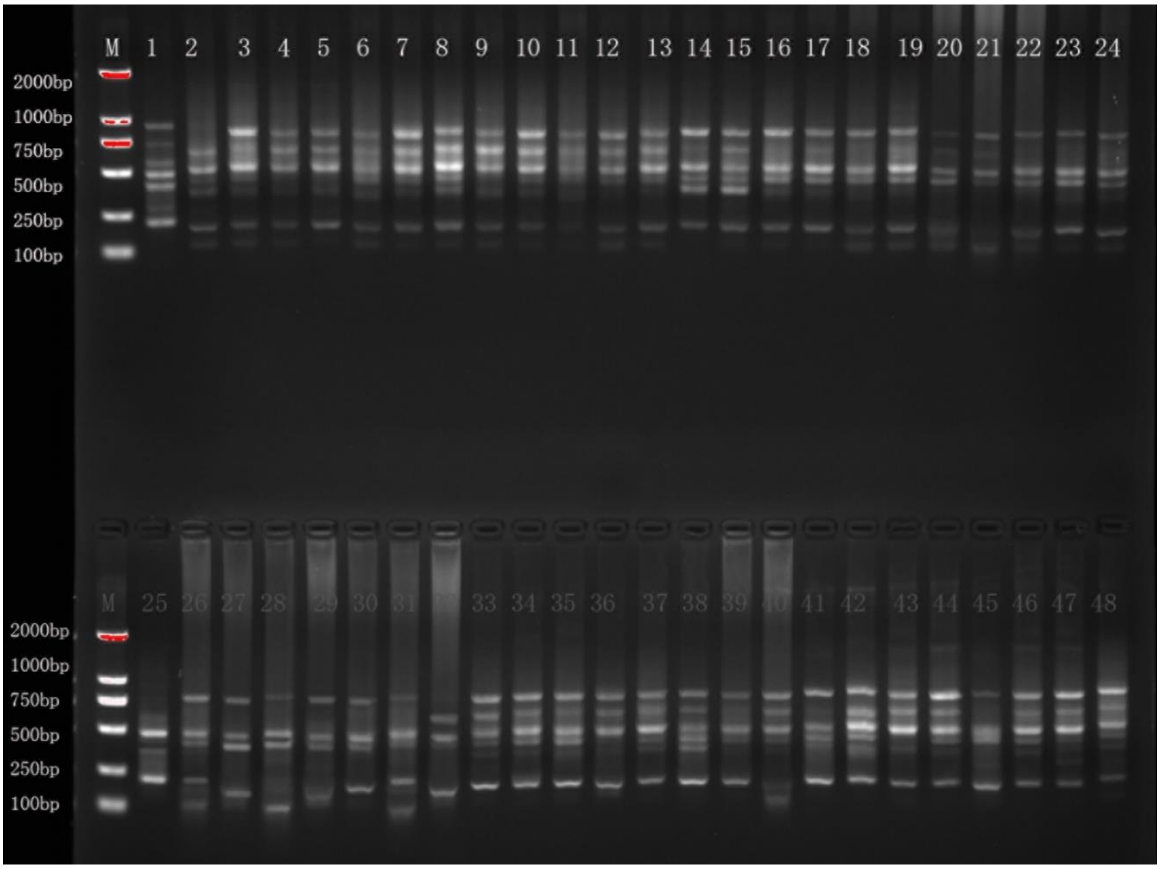

7. Amplification effect of primer ScoT 11 on *A.sinesis* samples (Voucher No. AS 1-48)

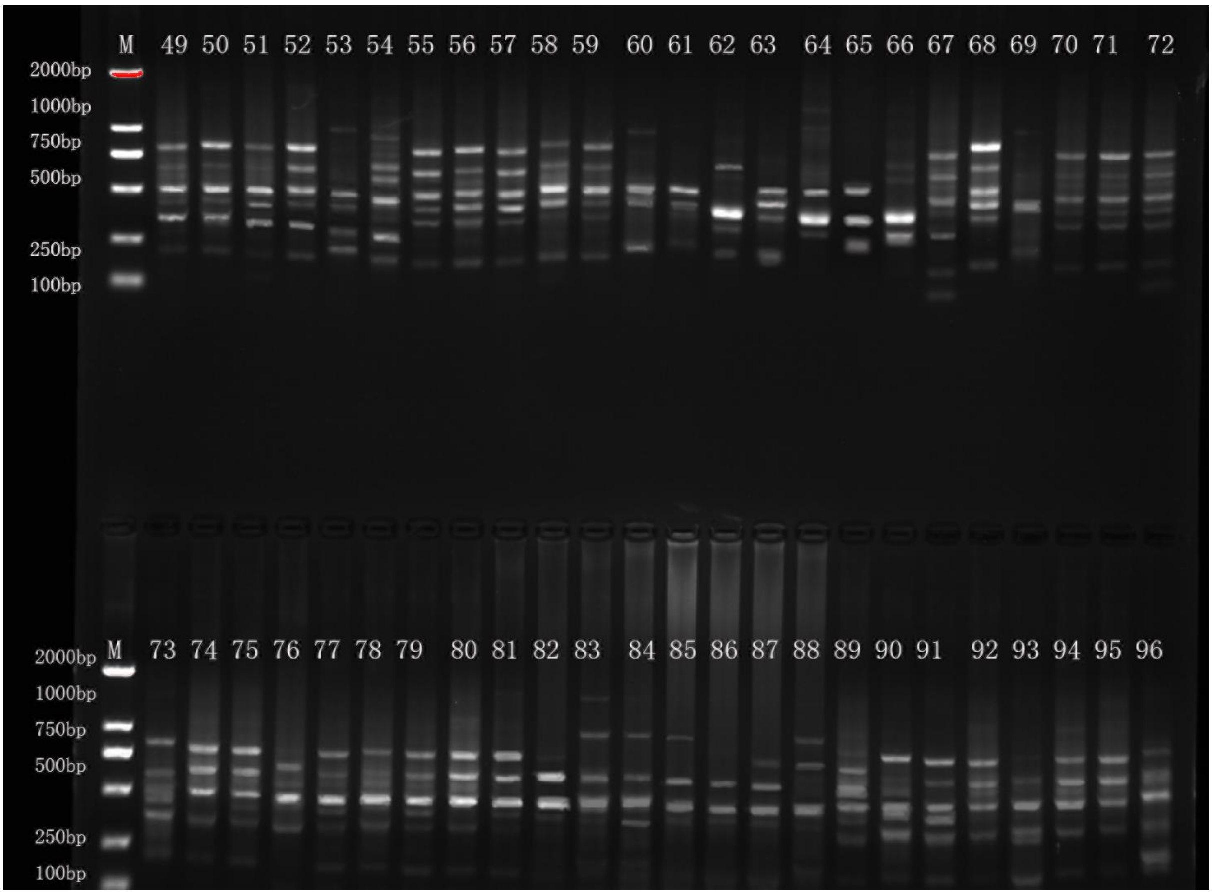

8. Amplification effect of primer ScoT 11 on *A.sinesis* samples (Voucher No. AS 49-96)

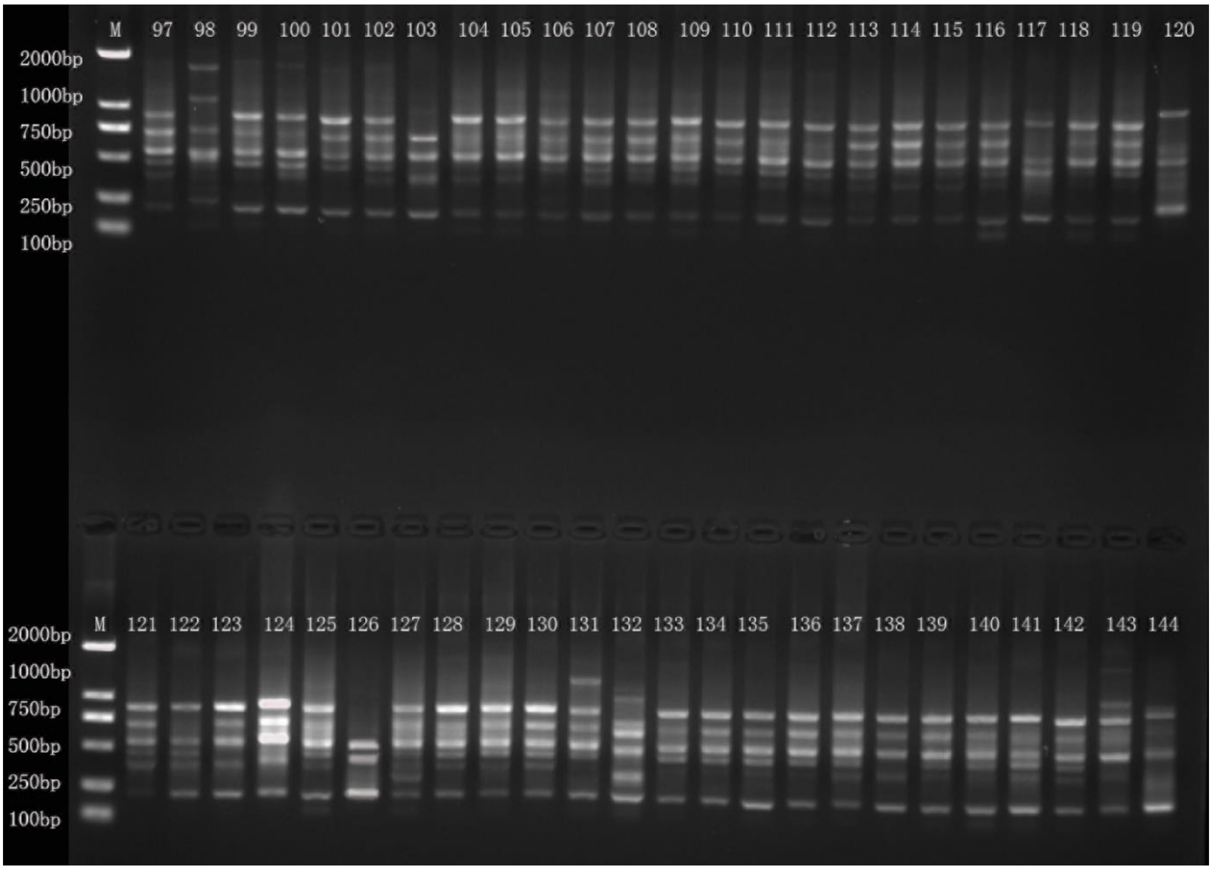

9. Amplification effect of primer ScoT 11 on *A.sinesis* samples (Voucher No. AS 97-144)

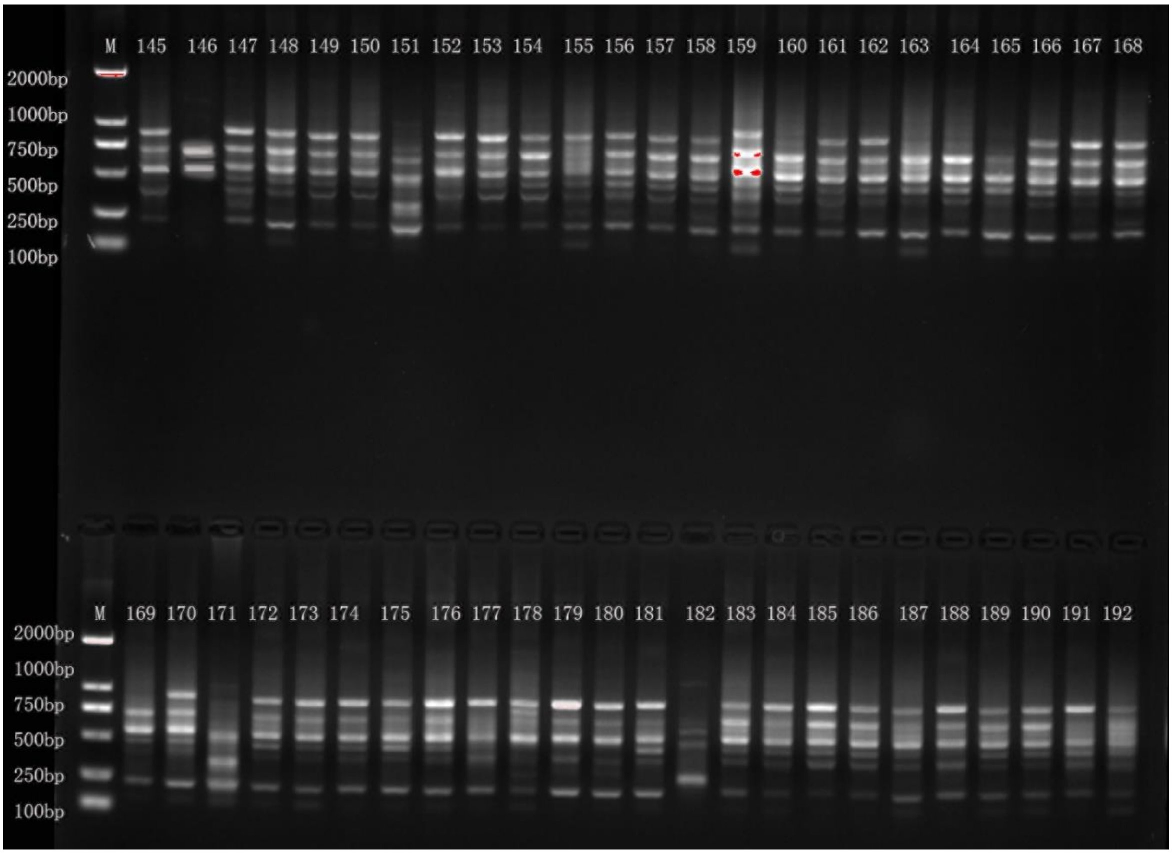

10. Amplification effect of primer ScoT 11 on *A.sinesis* samples (Voucher No. AS 145-192)

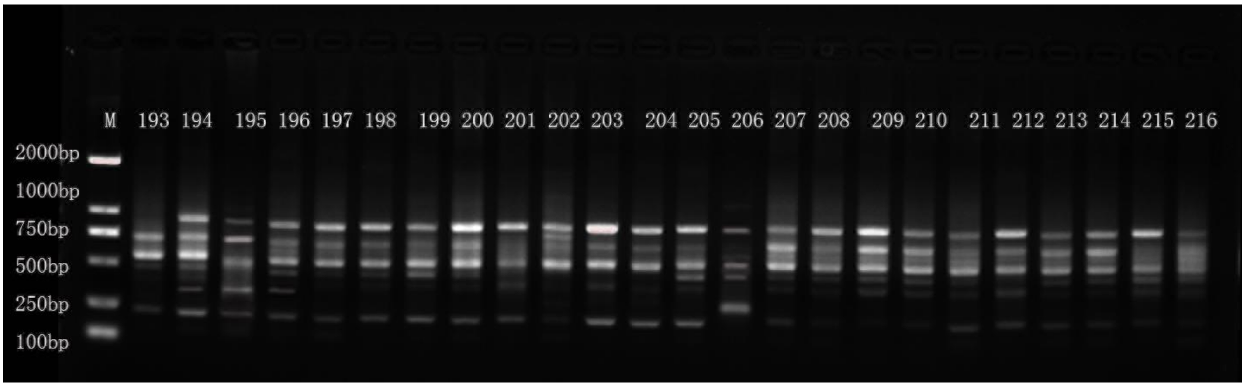

11. Amplification effect of primer ScoT 11 on *A.sinesis* samples (Voucher No. AS 193-216)

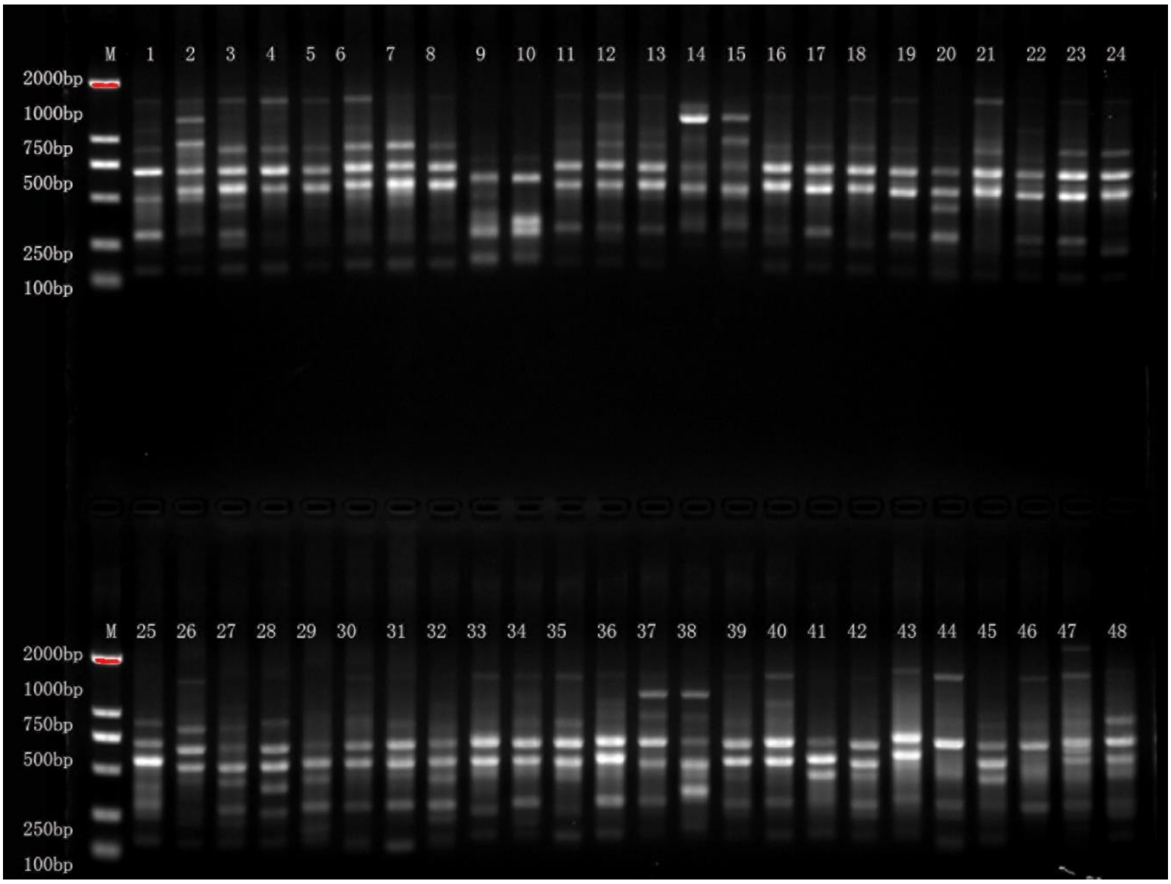

12. Amplification effect of primer ScoT 12 on *A.sinesis* samples (Voucher No. AS 1-48)

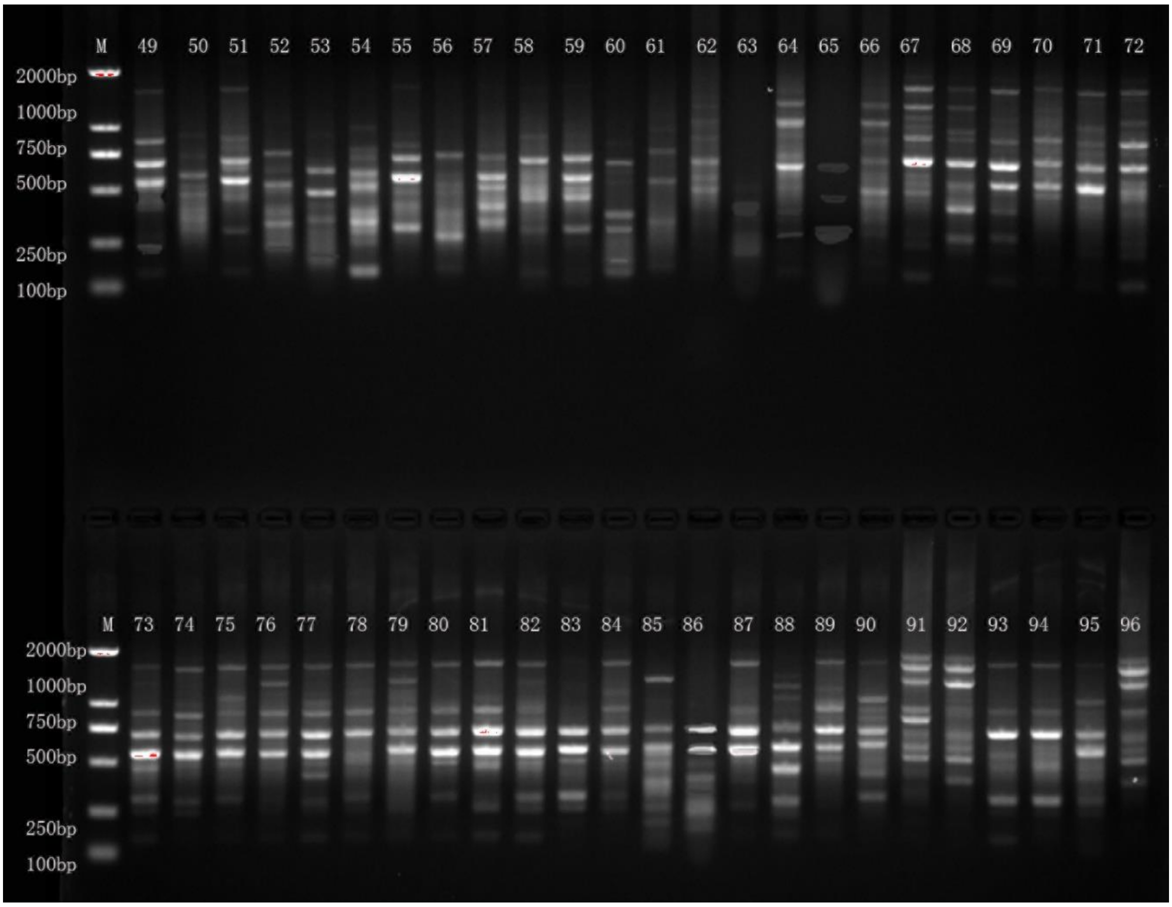

13. Amplification effect of primer ScoT 12 on *A.sinesis* samples (Voucher No. AS 49-96)

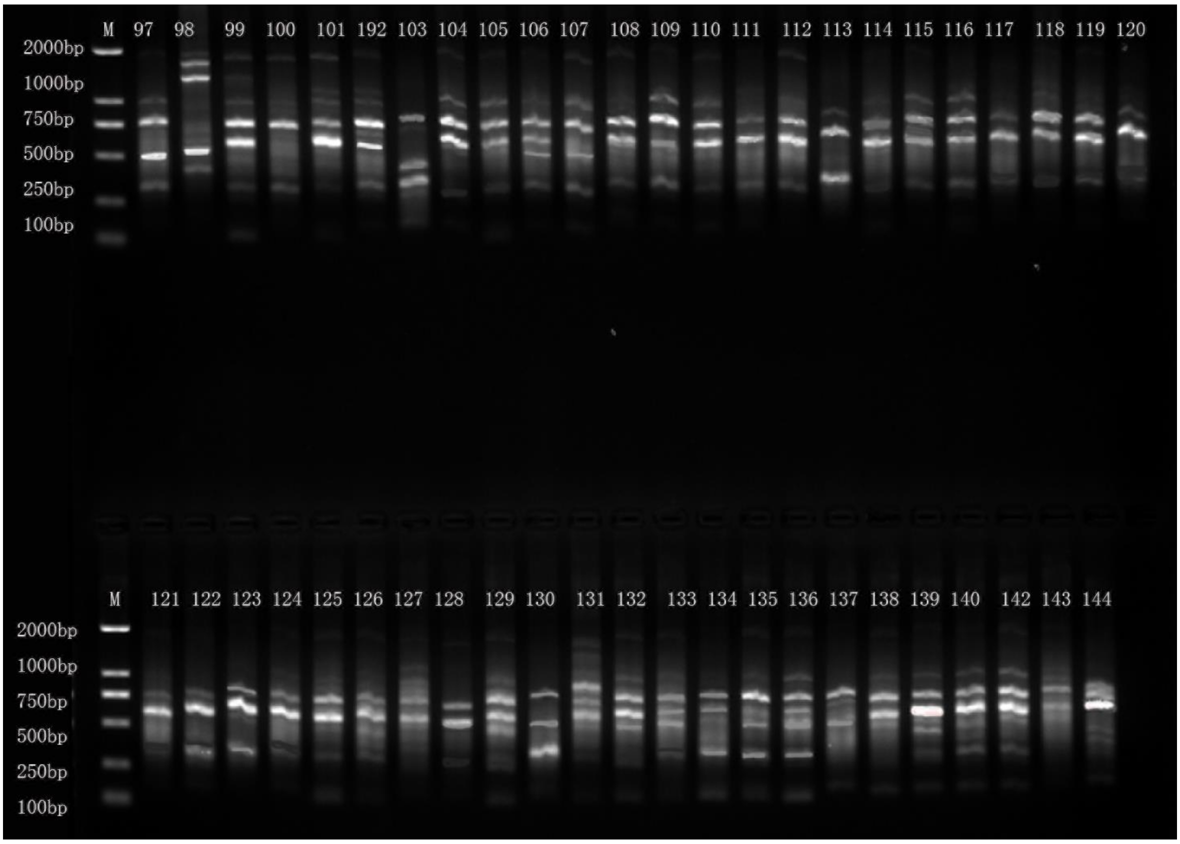

14. Amplification effect of primer ScoT 12 on *A.sinesis* samples (Voucher No. AS 97-144)

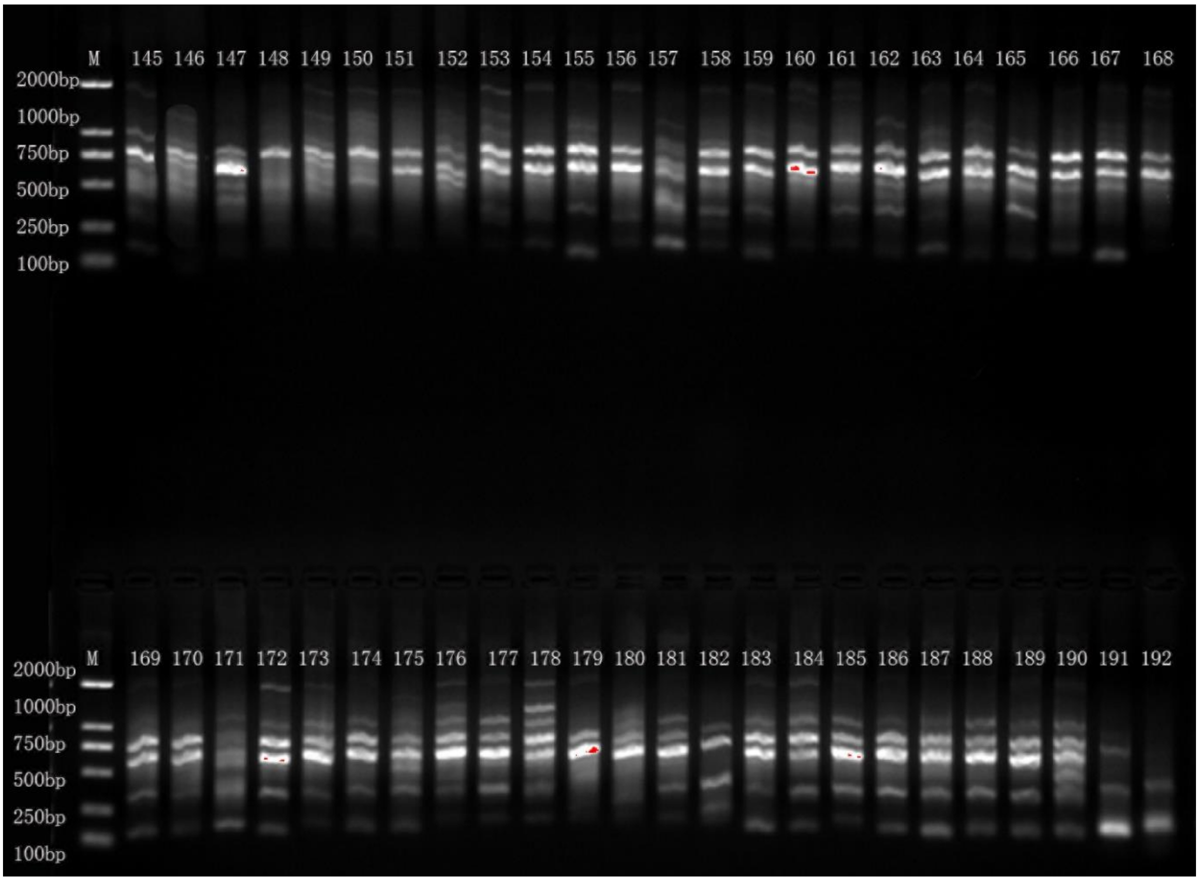

15. Amplification effect of primer ScoT 12 on *A.sinesis* samples (Voucher No. AS 145-192)

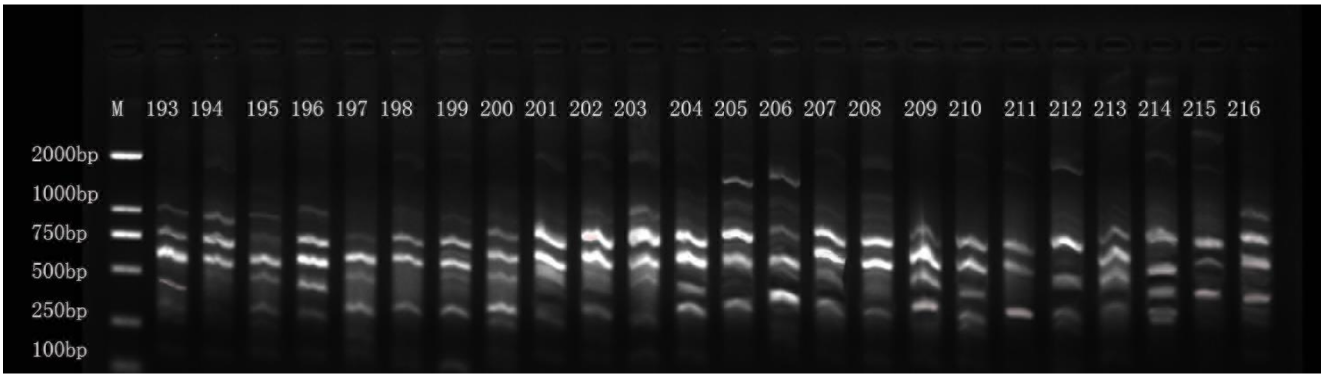

16. Amplification effect of primer ScoT 12 on *A.sinesis* samples (Voucher No. AS 193-216)

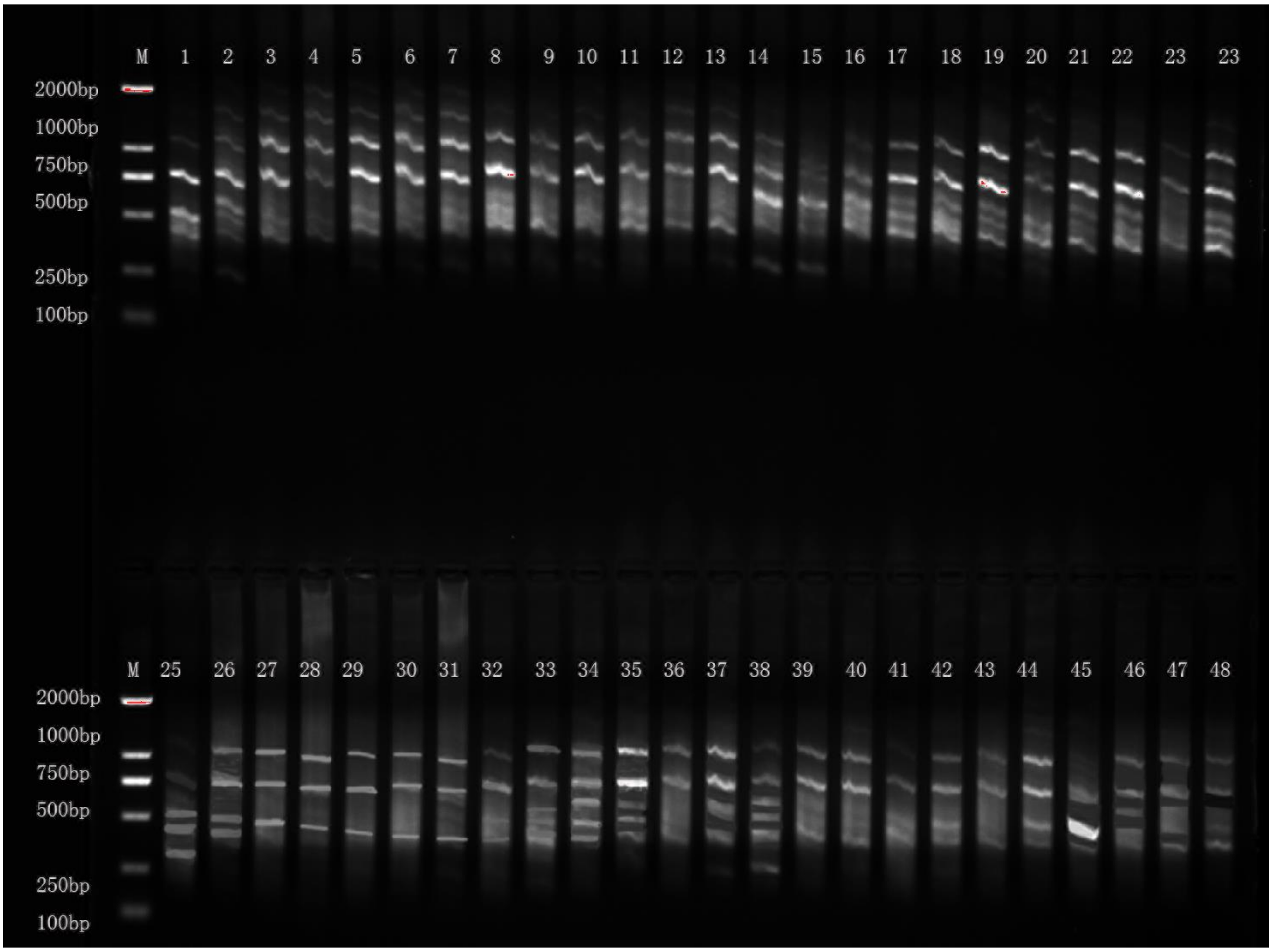

17. Amplification effect of primer ScoT 14 on *A.sinesis* samples (Voucher No. AS 1-48)

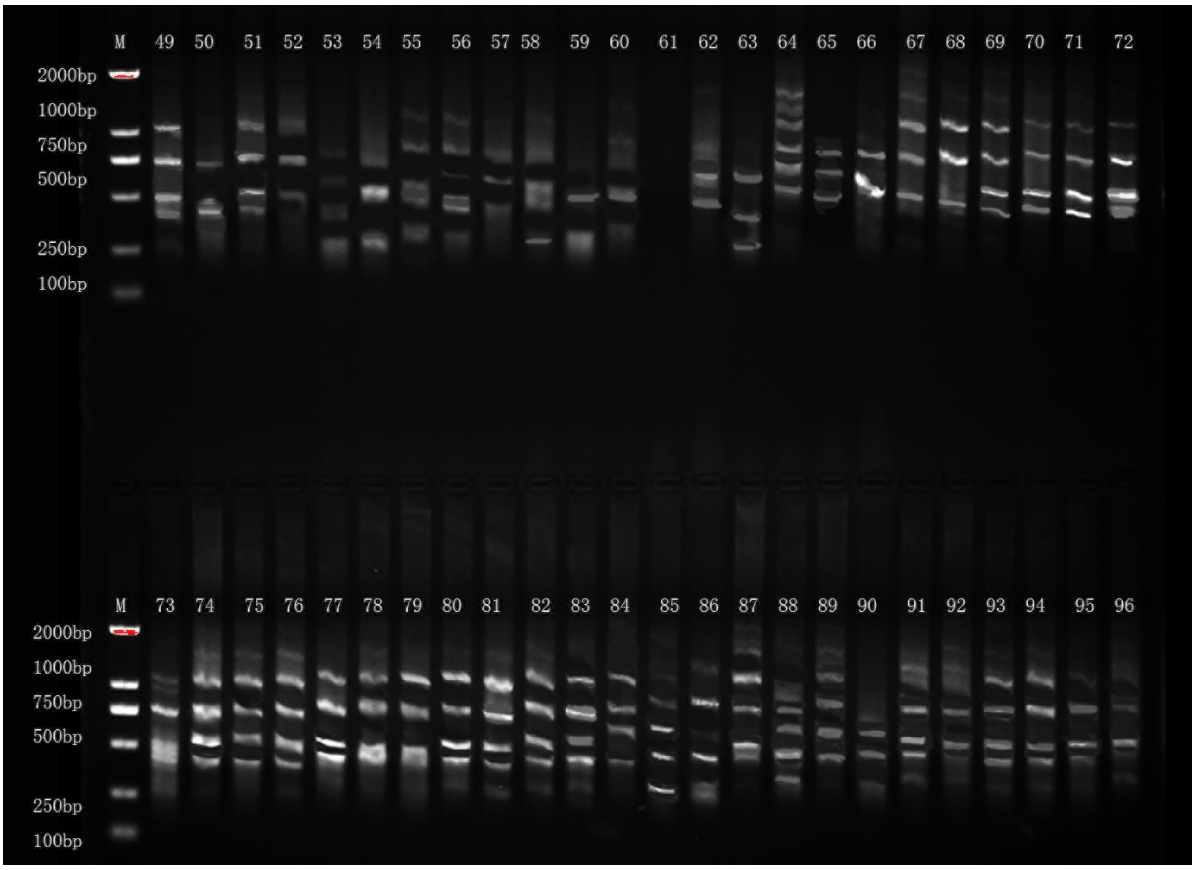

18. Amplification effect of primer ScoT 14 on *A.sinesis* samples (Voucher No. AS 49-96)

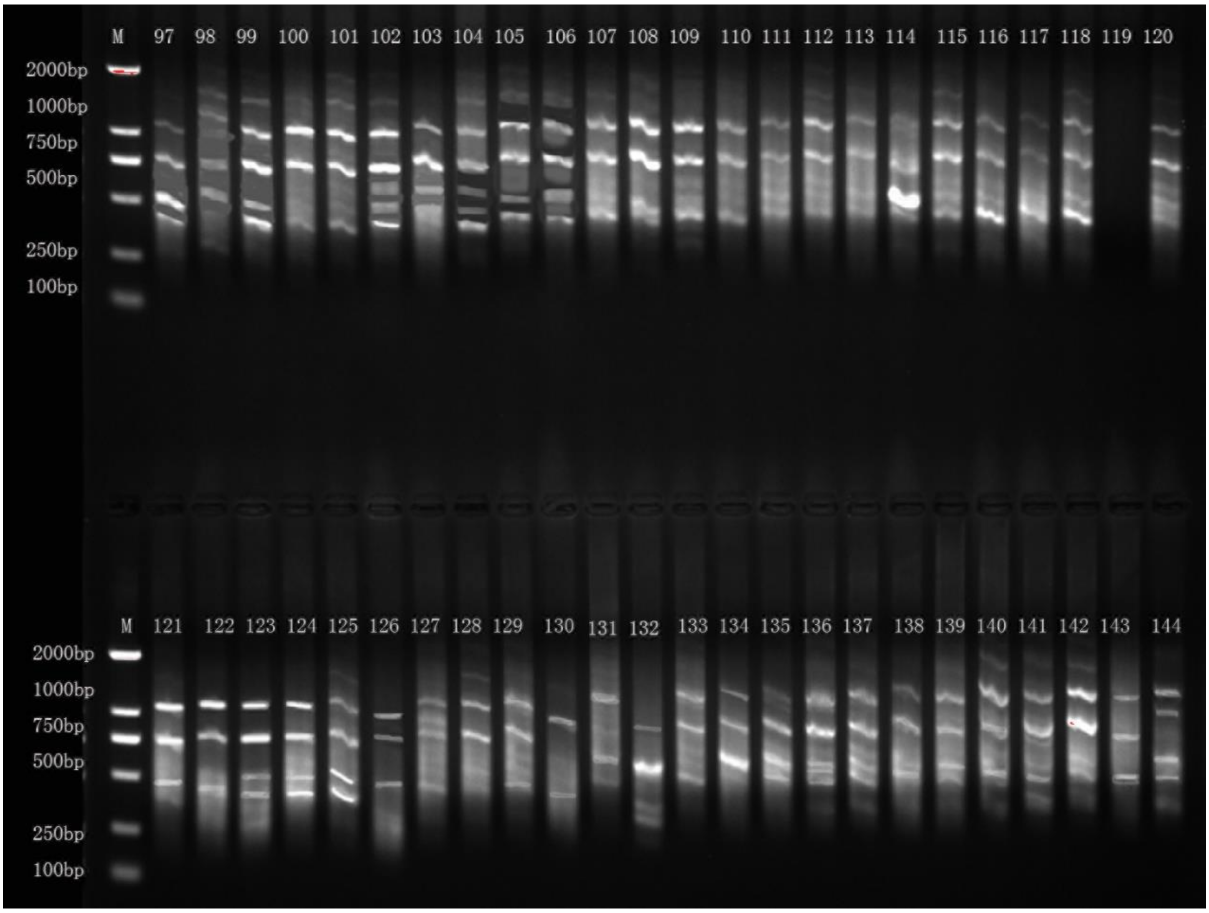

19. Amplification effect of primer ScoT 14 on A.sinesis samples (Voucher No. AS 97-144)

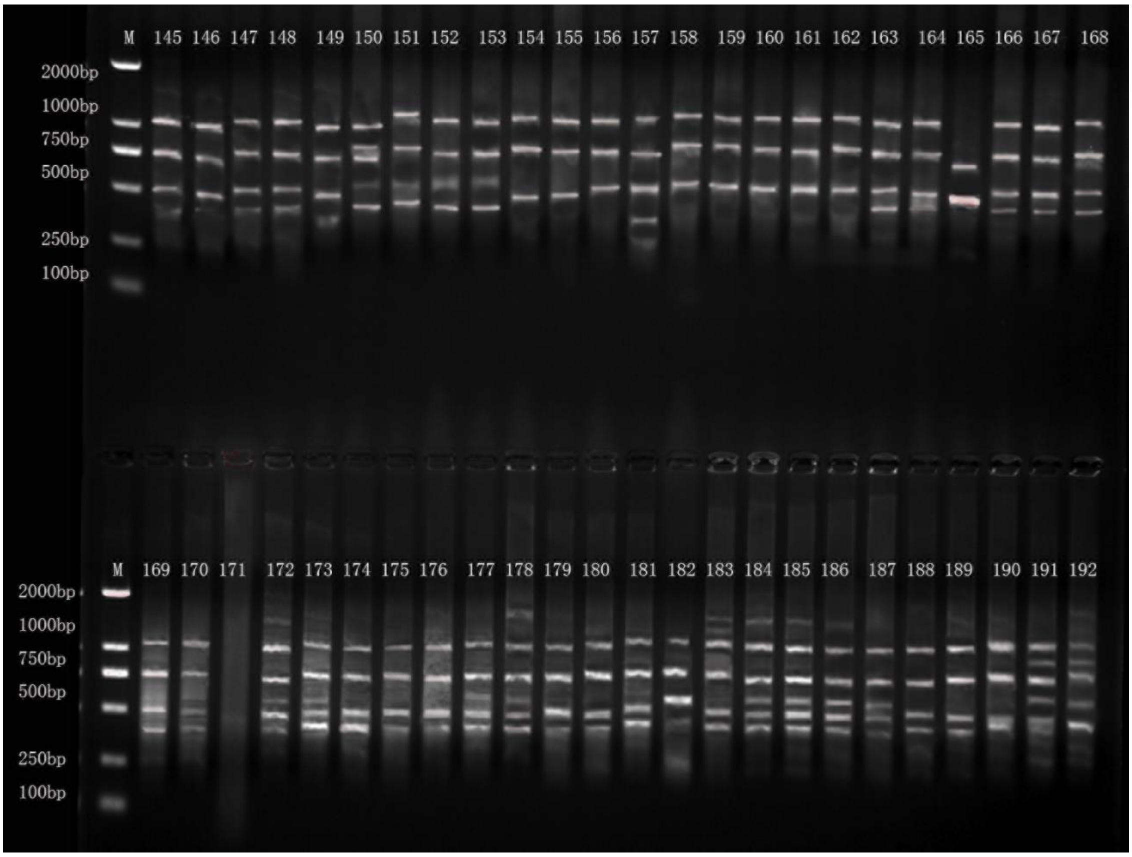

20. Amplification effect of primer ScoT 14 on *A.sinesis* samples (Voucher No. AS 145-192)

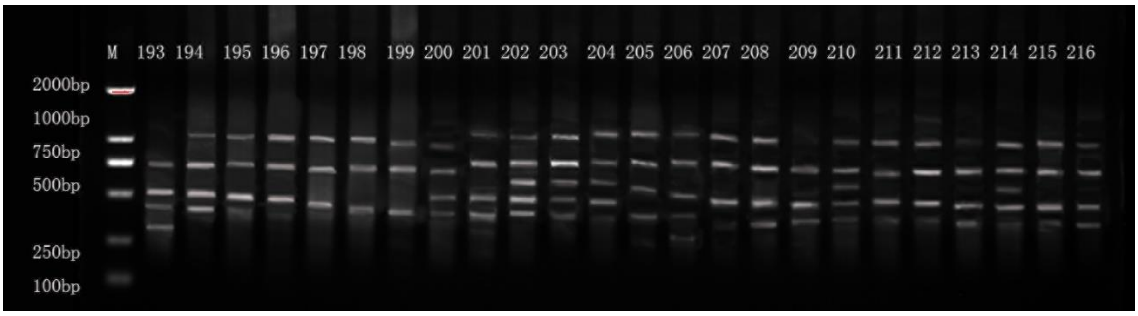

21. Amplification effect of primer ScoT 14 on *A.sinesis* samples (Voucher No. AS 193-216)

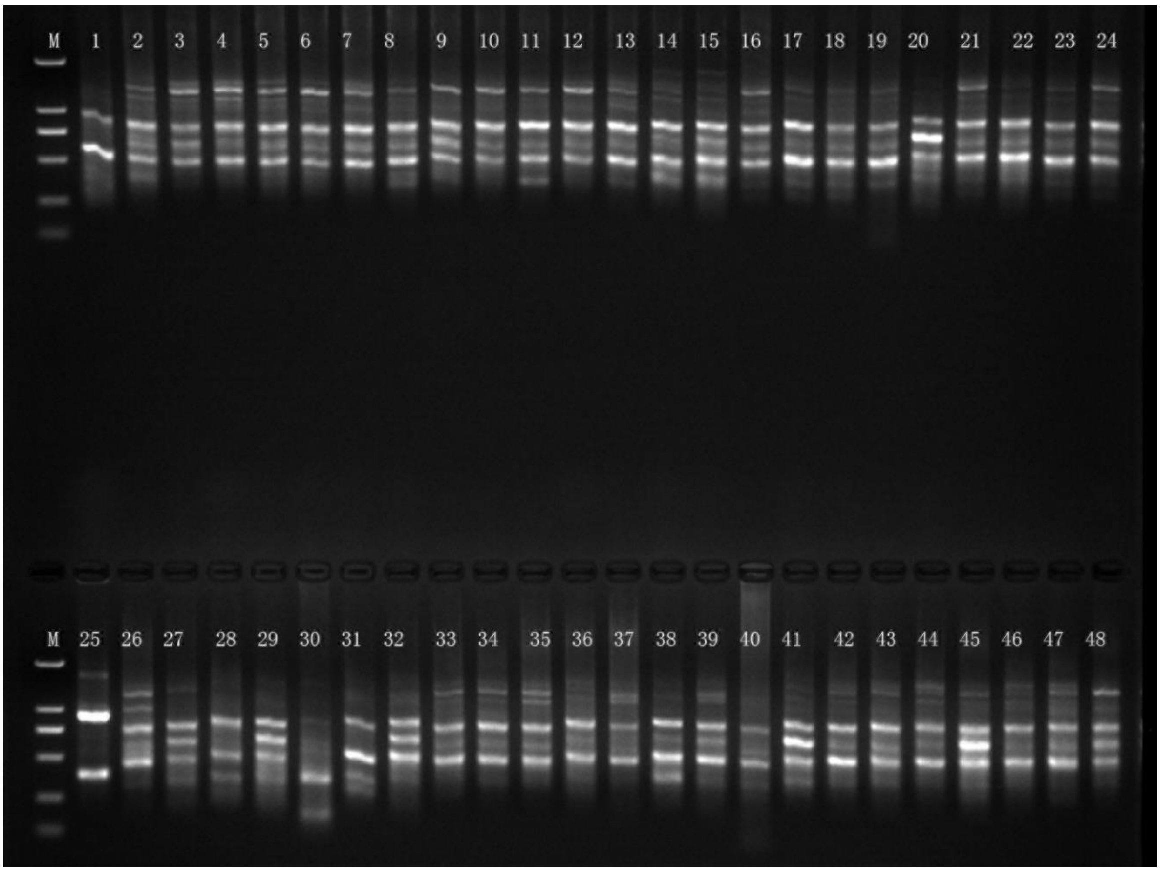

22. Amplification effect of primer ScoT 17 on *A.sinesis* samples (Voucher No. AS 1-48)

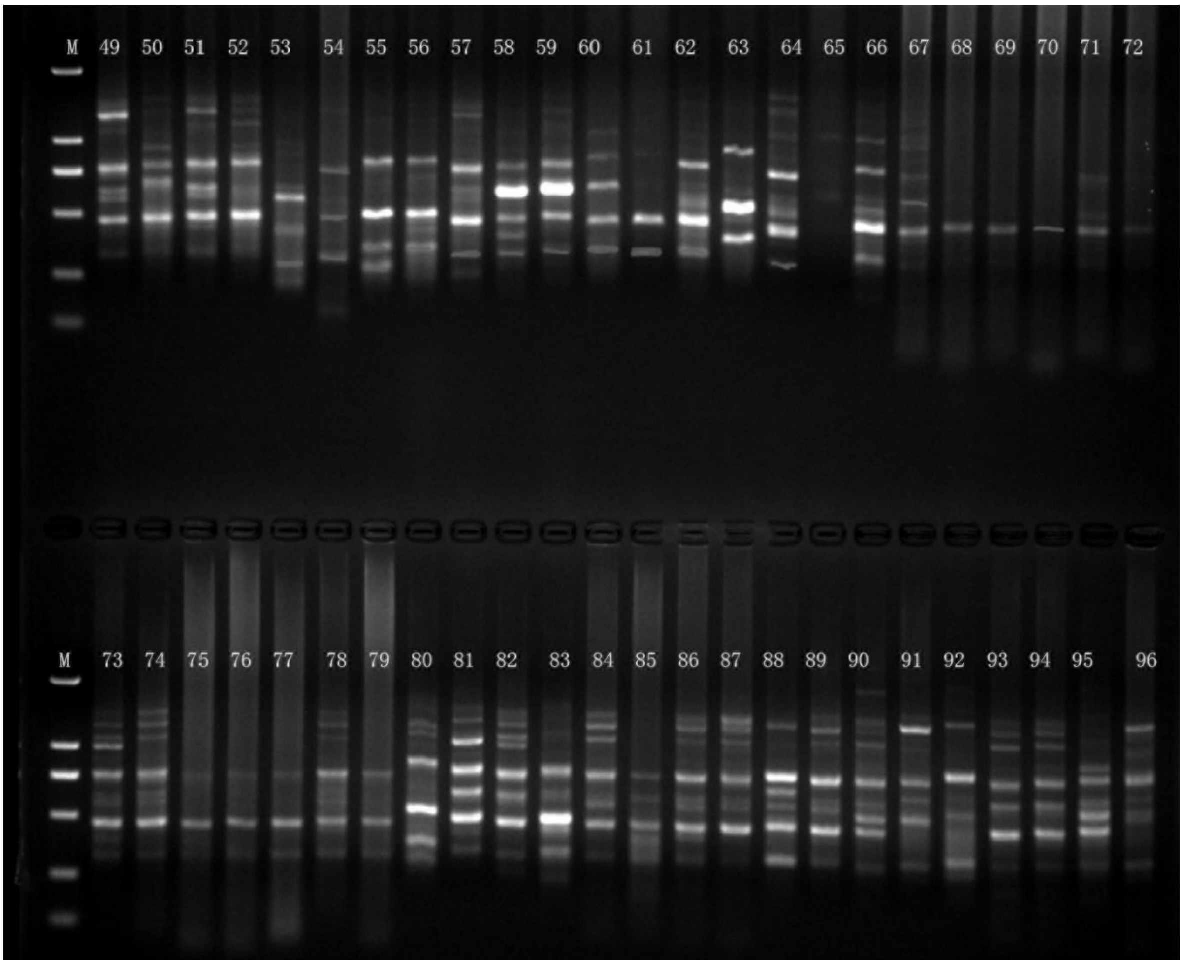

23. Amplification effect of primer ScoT 17 on A.sinesis samples (Voucher No. AS 49-96)

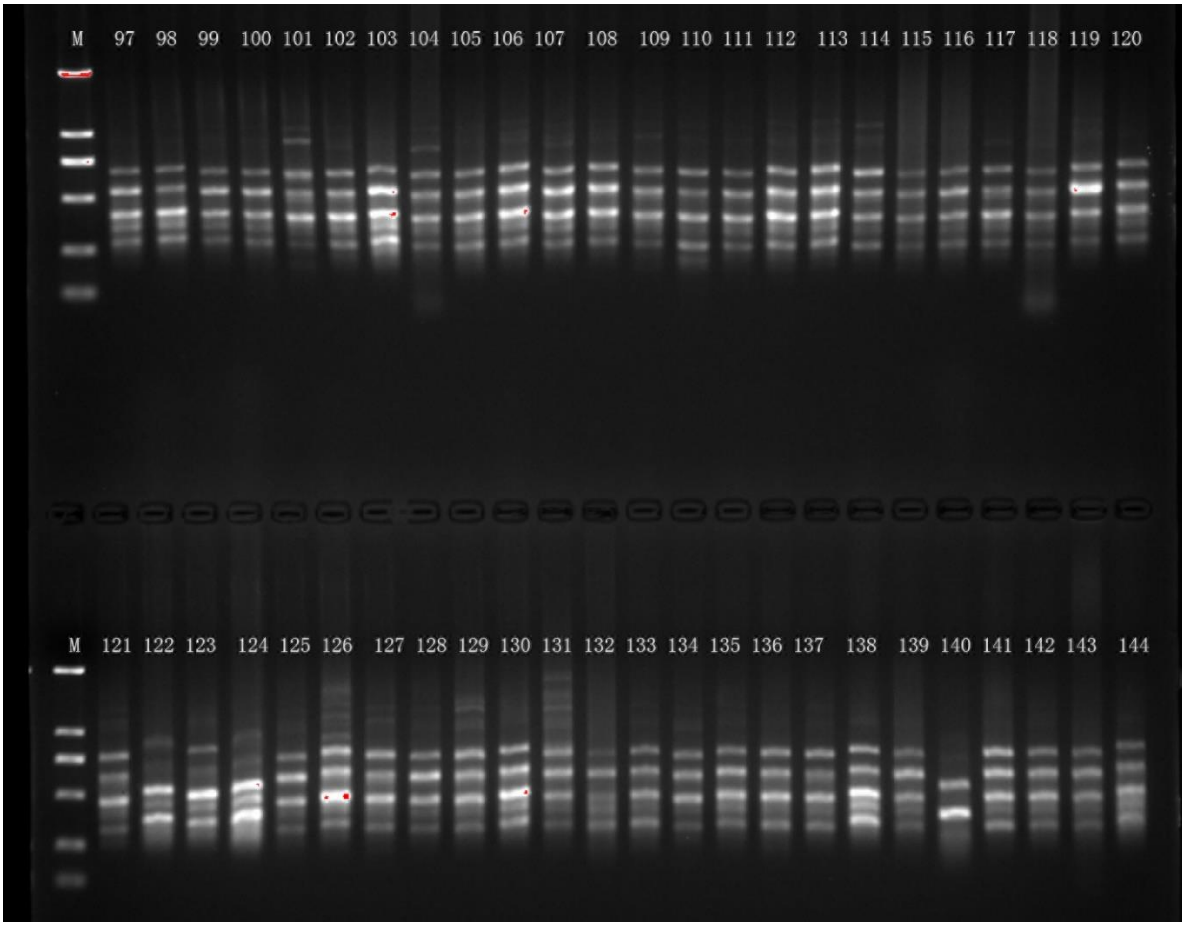

24. Amplification effect of primer ScoT 17 on A.sinesis samples (Voucher No. AS 97-144)

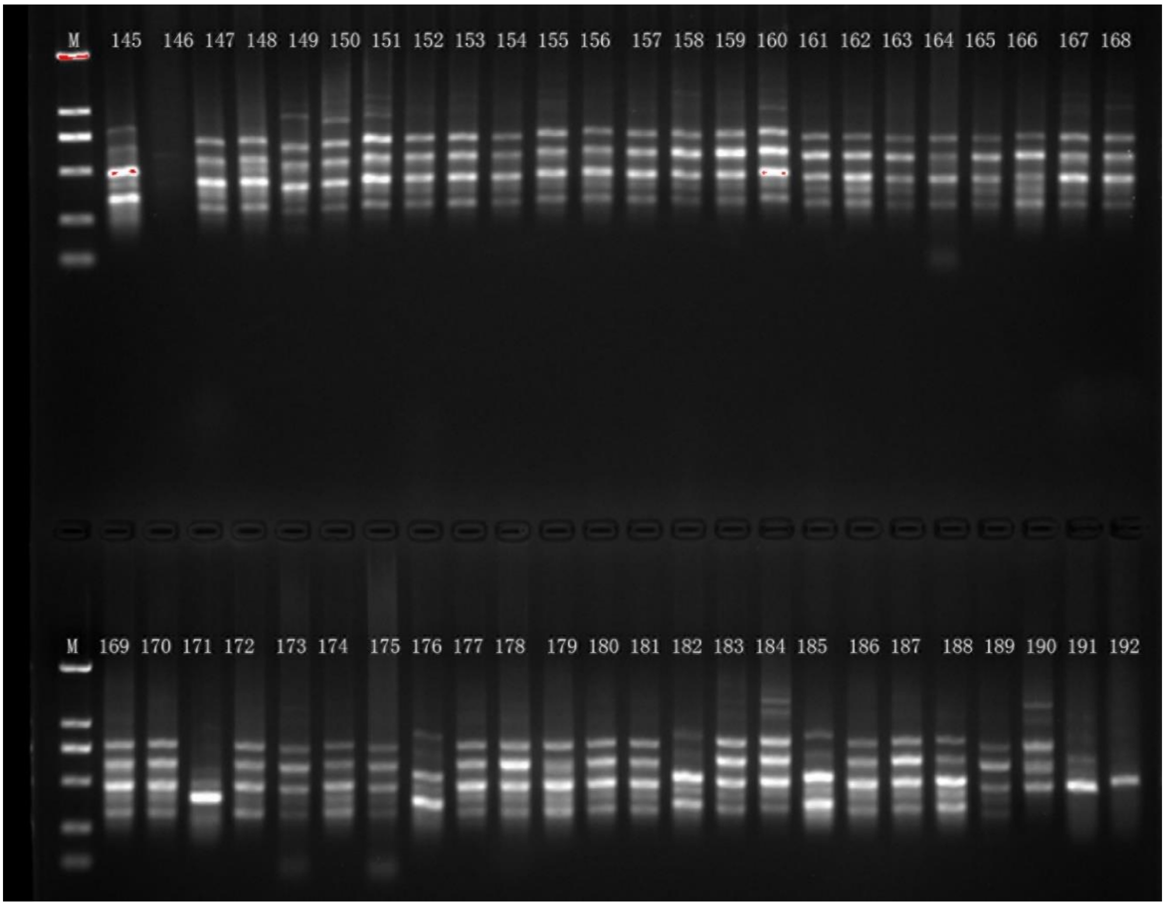

25. Amplification effect of primer ScoT 17 on A.sinesis samples (Voucher No. AS 145-192)

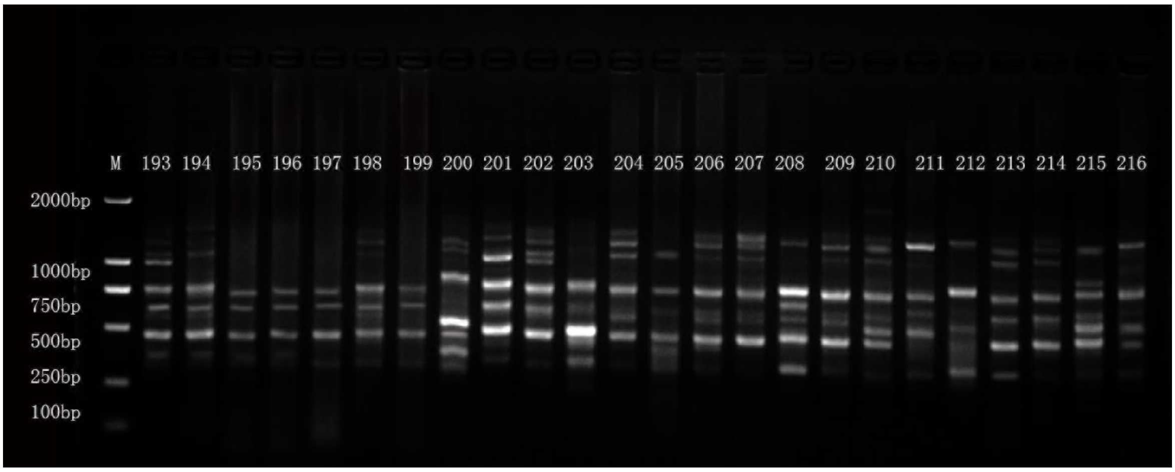

26. Amplification effect of primer ScoT 17 on *A.sinesis* samples (Voucher No. AS 193-216)

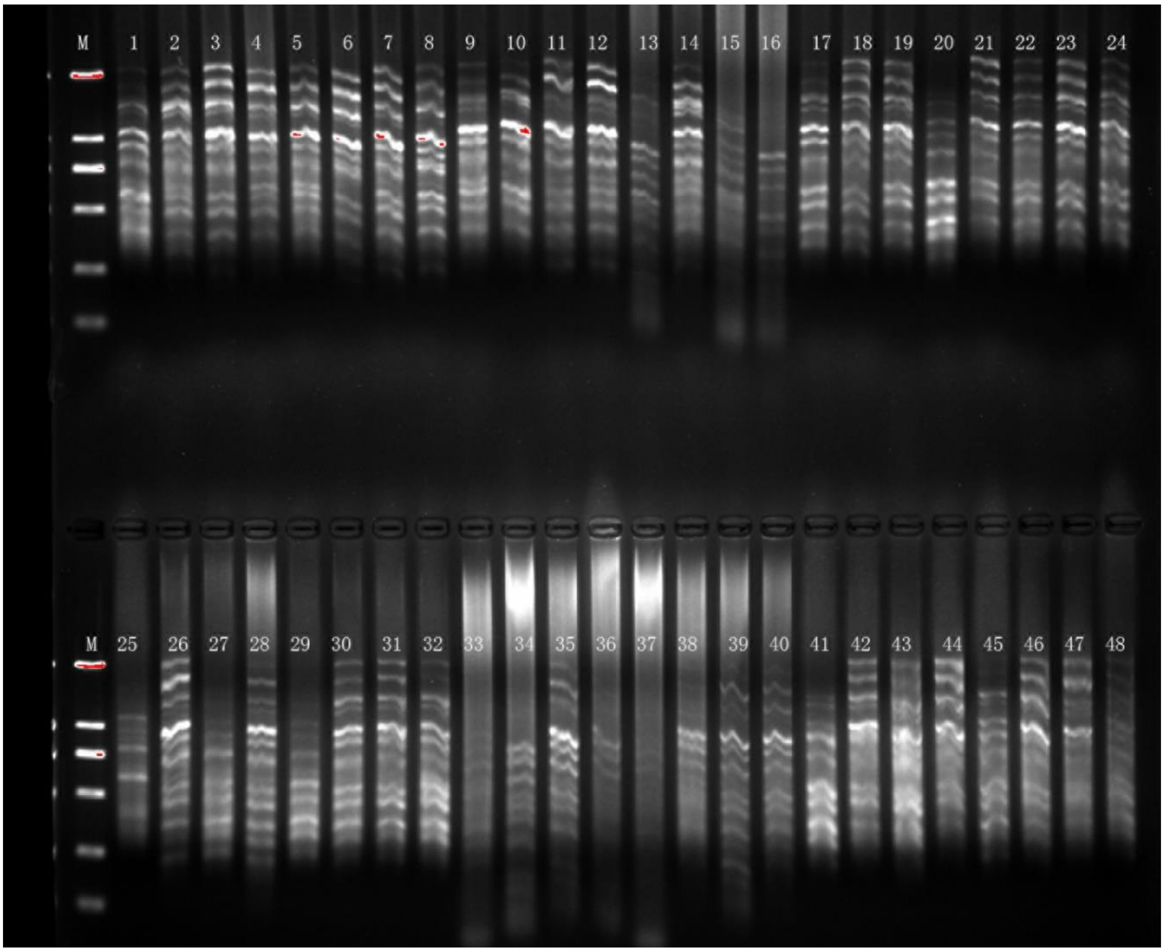

27. Amplification effect of primer ScoT 18 on *A.sinesis* samples (Voucher No. AS 1-48)

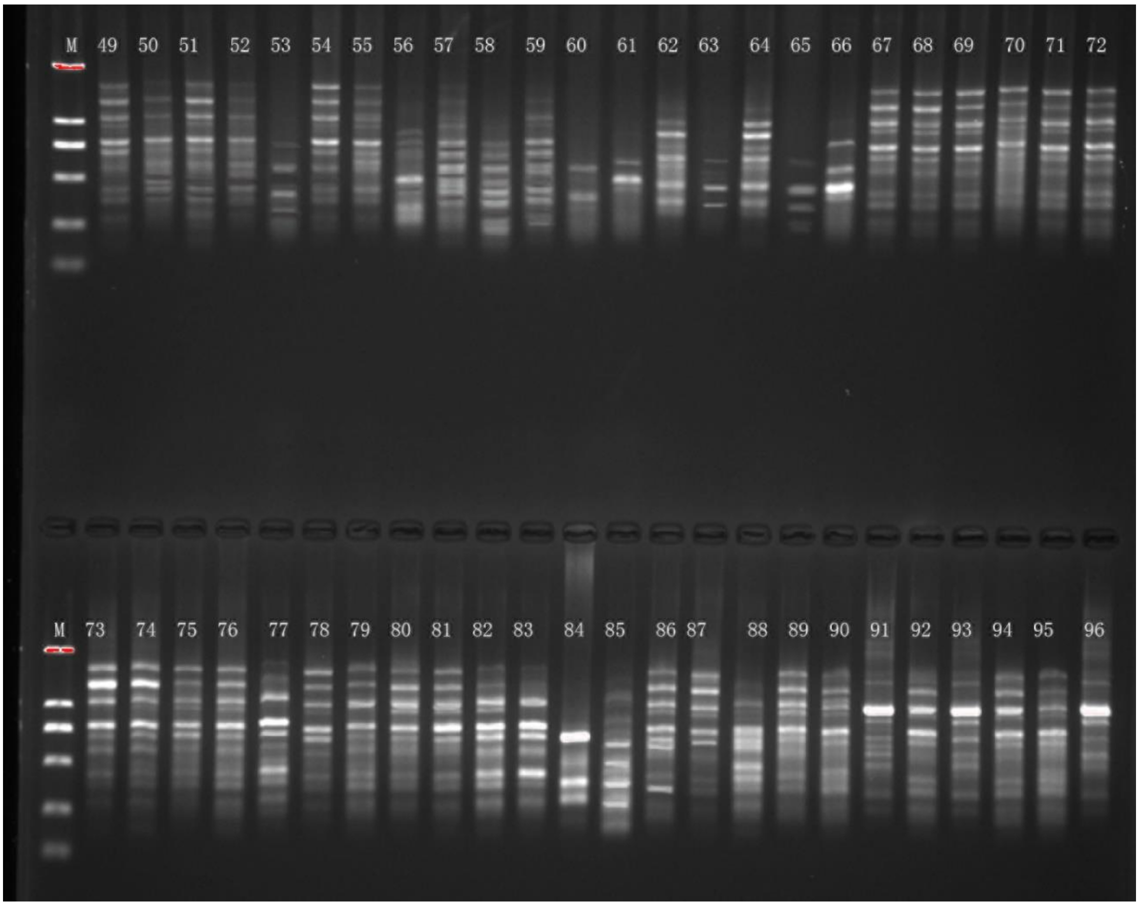

28. Amplification effect of primer ScoT 18 on *A.sinesis* samples (Voucher No. AS 49-96)

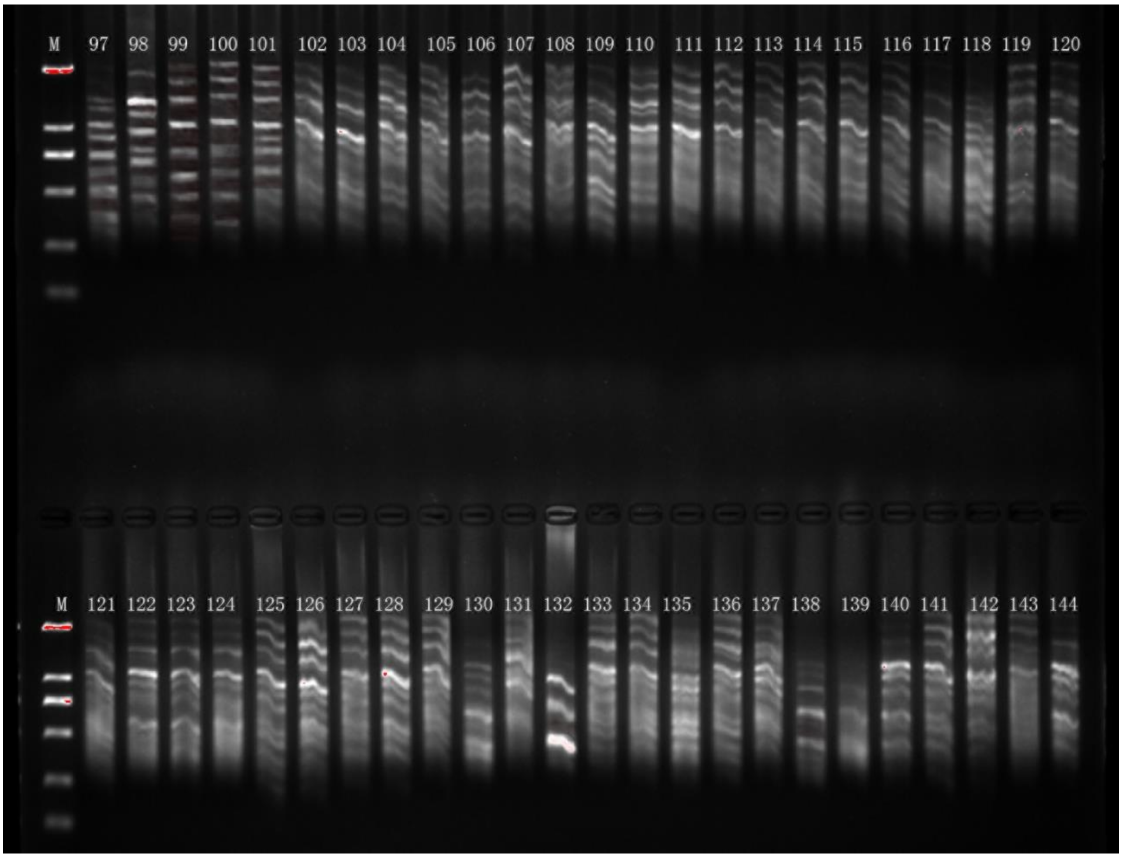

29. Amplification effect of primer ScoT 18 on *A.sinesis* samples (Voucher No. AS 97-144)

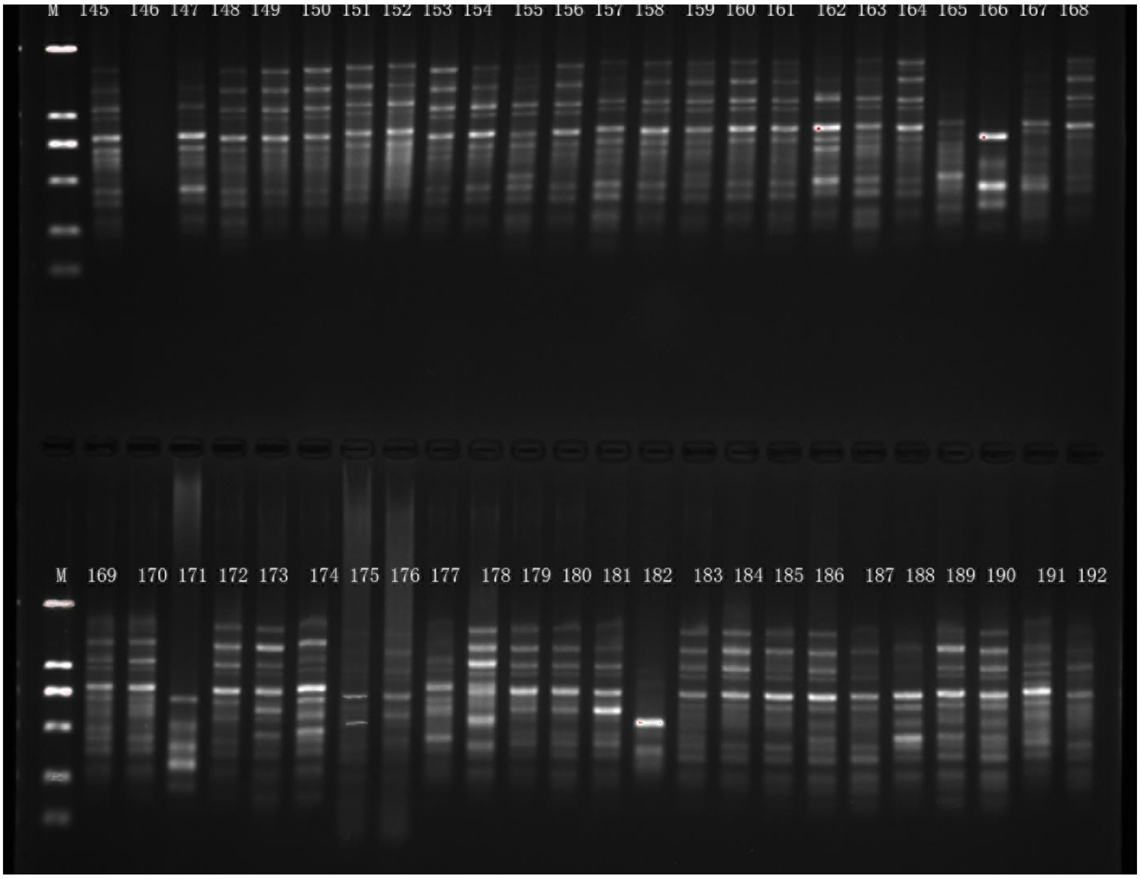

30. Amplification effect of primer ScoT 18 on *A.sinesis* samples (Voucher No. AS 145-192)

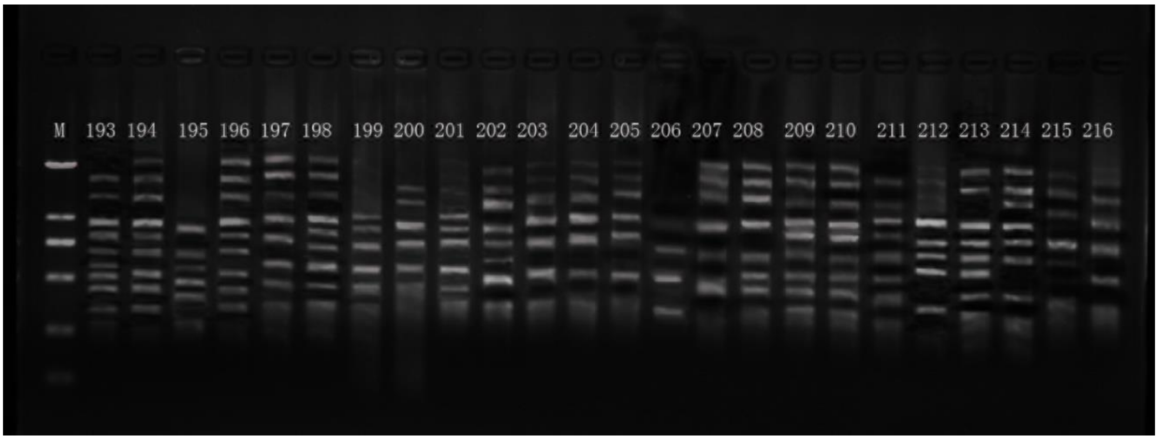

31. Amplification effect of primer ScoT 18 on *A.sinesis* samples (Voucher No. AS 193-216)

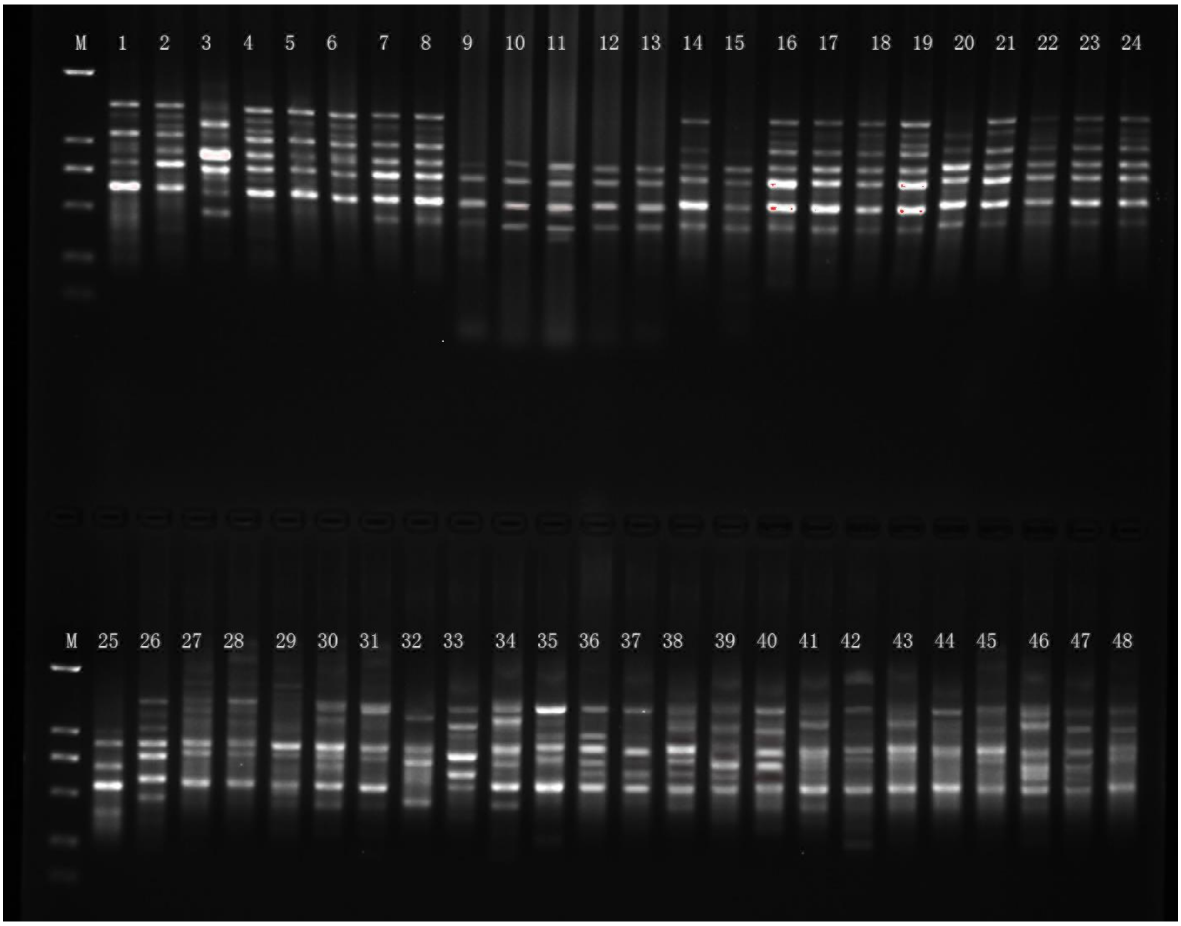

32. Amplification effect of primer ScoT 19 on *A.sinesis* samples (Voucher No. AS 1-48)

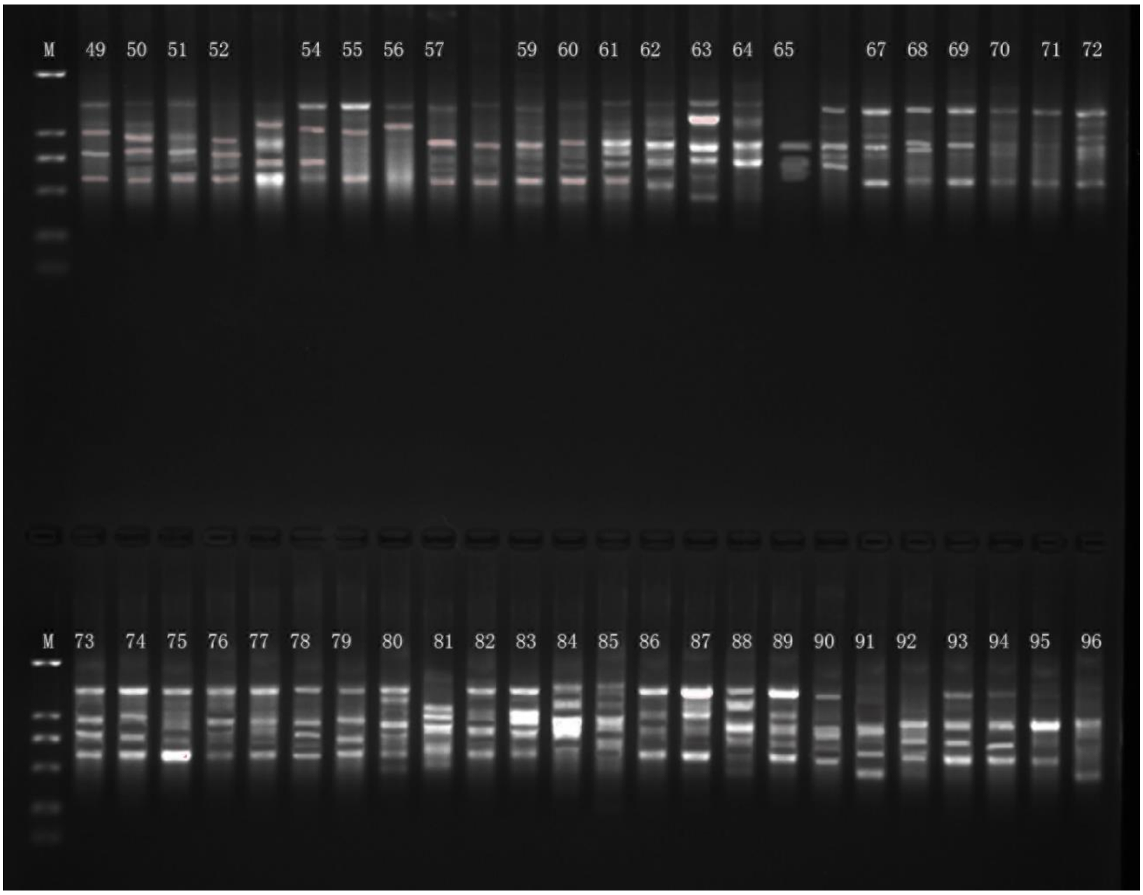

33. Amplification effect of primer ScoT 19 on *A.sinesis* samples (Voucher No. AS 49-96)

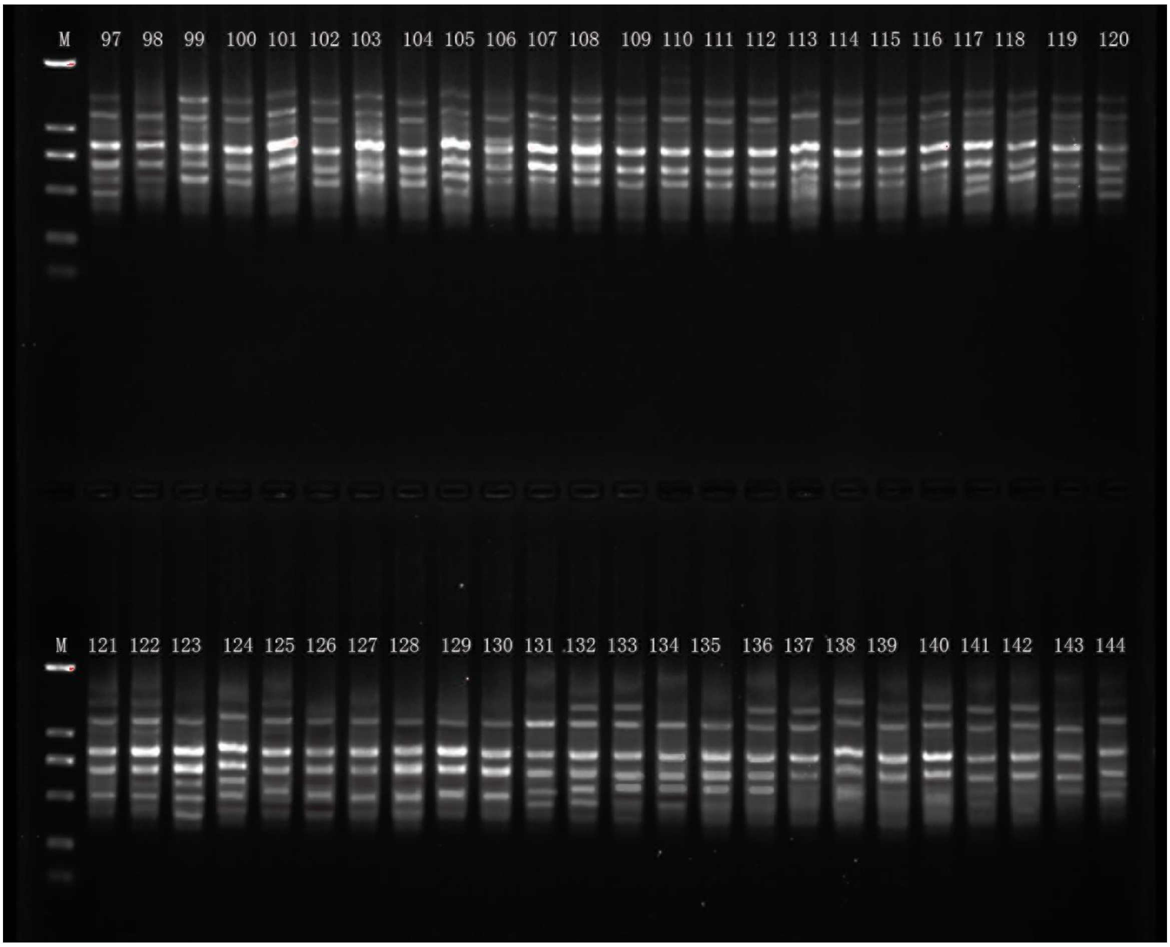

34. Amplification effect of primer ScoT 19 on *A.sinesis* samples (Voucher No. AS 97-144)

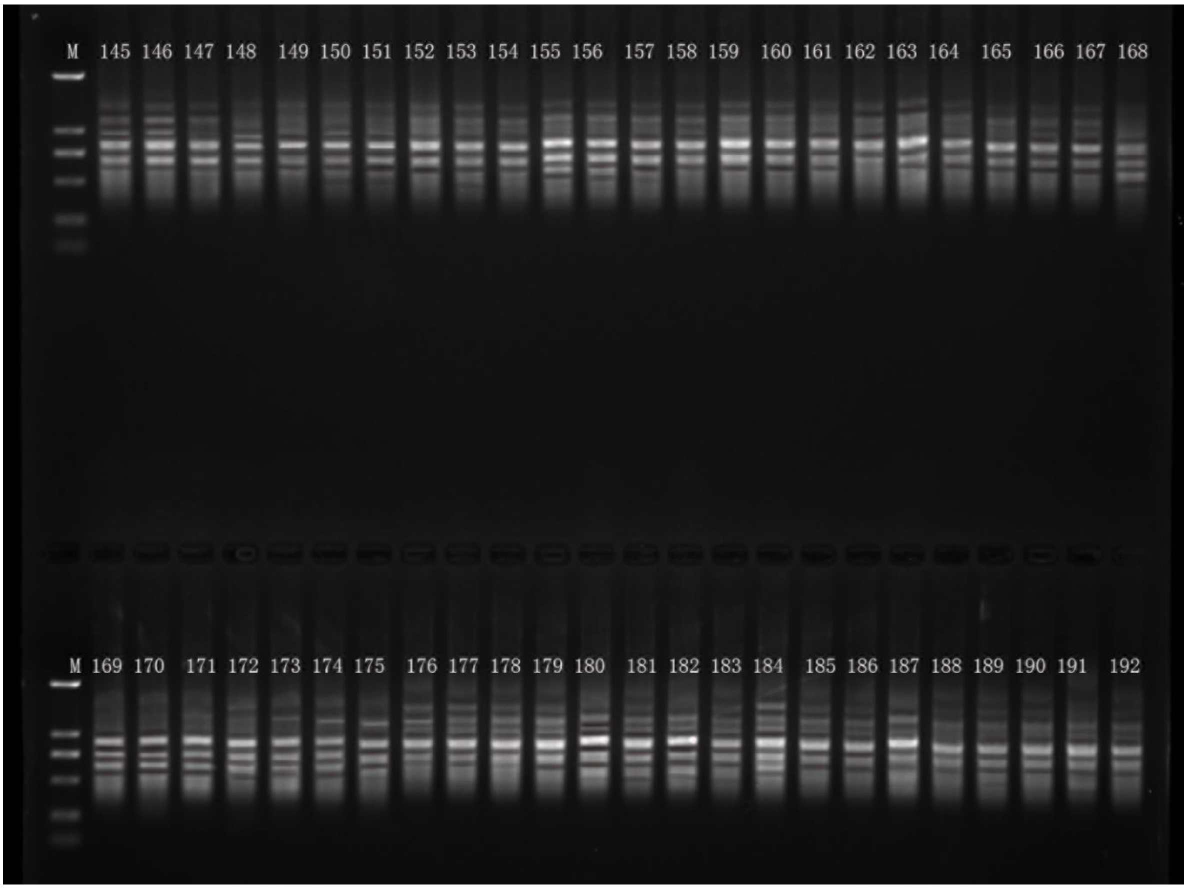

35. Amplification effect of primer ScoT 19 on *A.sinesis* samples (Voucher No. AS 145-192)

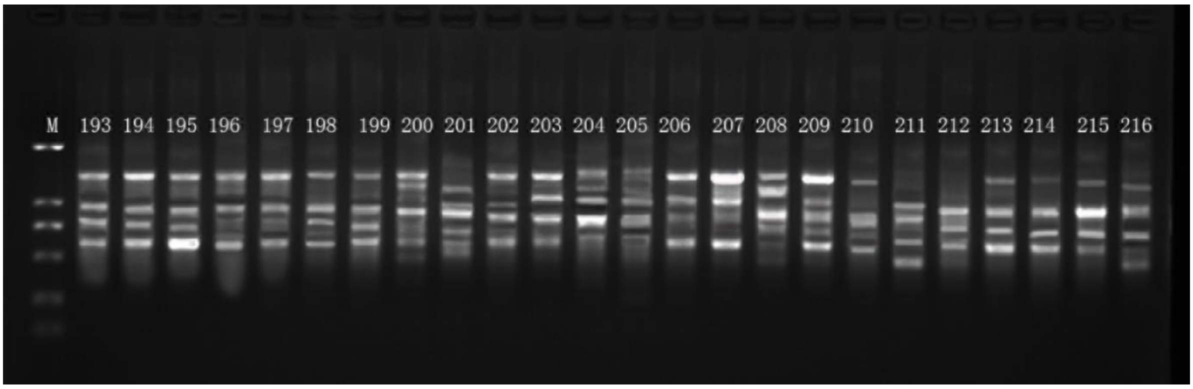

36. Amplification effect of primer ScoT 19 on *A.sinesis* samples (Voucher No. AS 193-216)

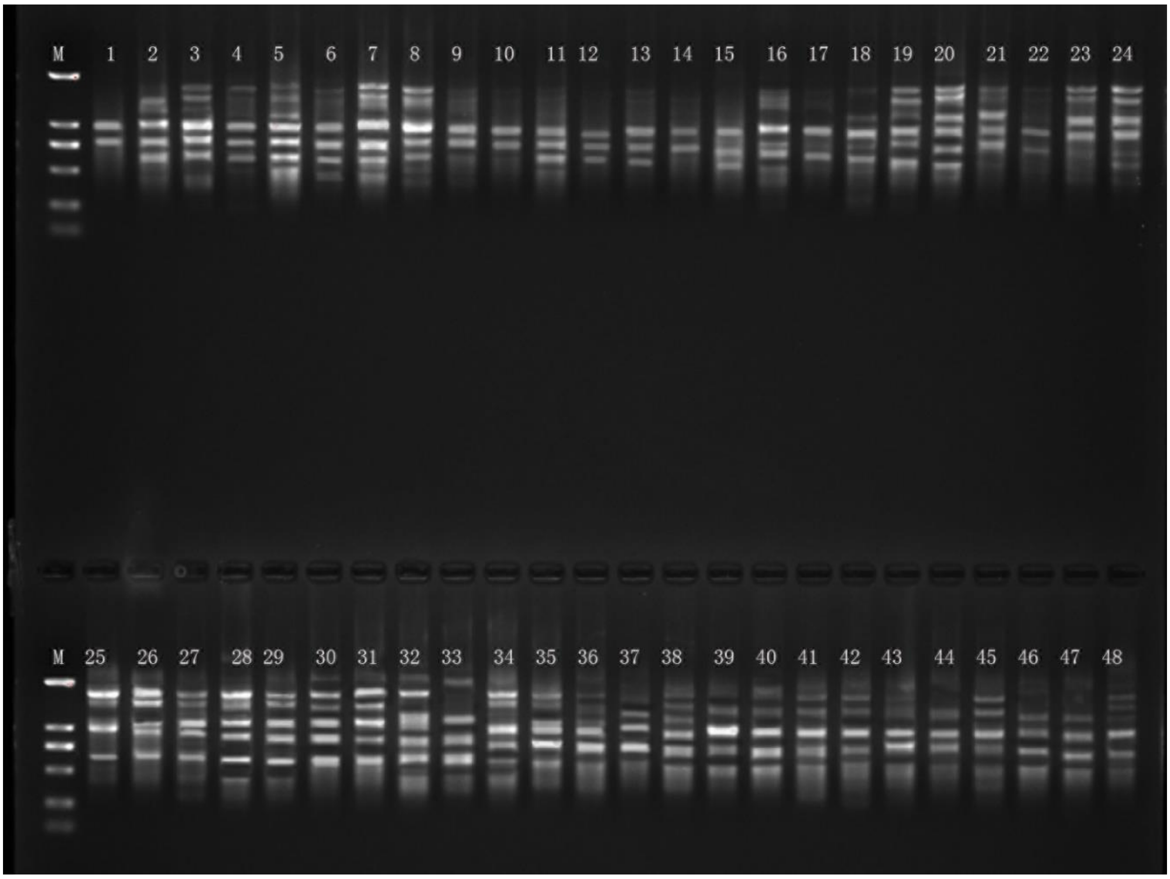

37. Amplification effect of primer ScoT 20 on *A.sinesis* samples (Voucher No. AS 1-48)

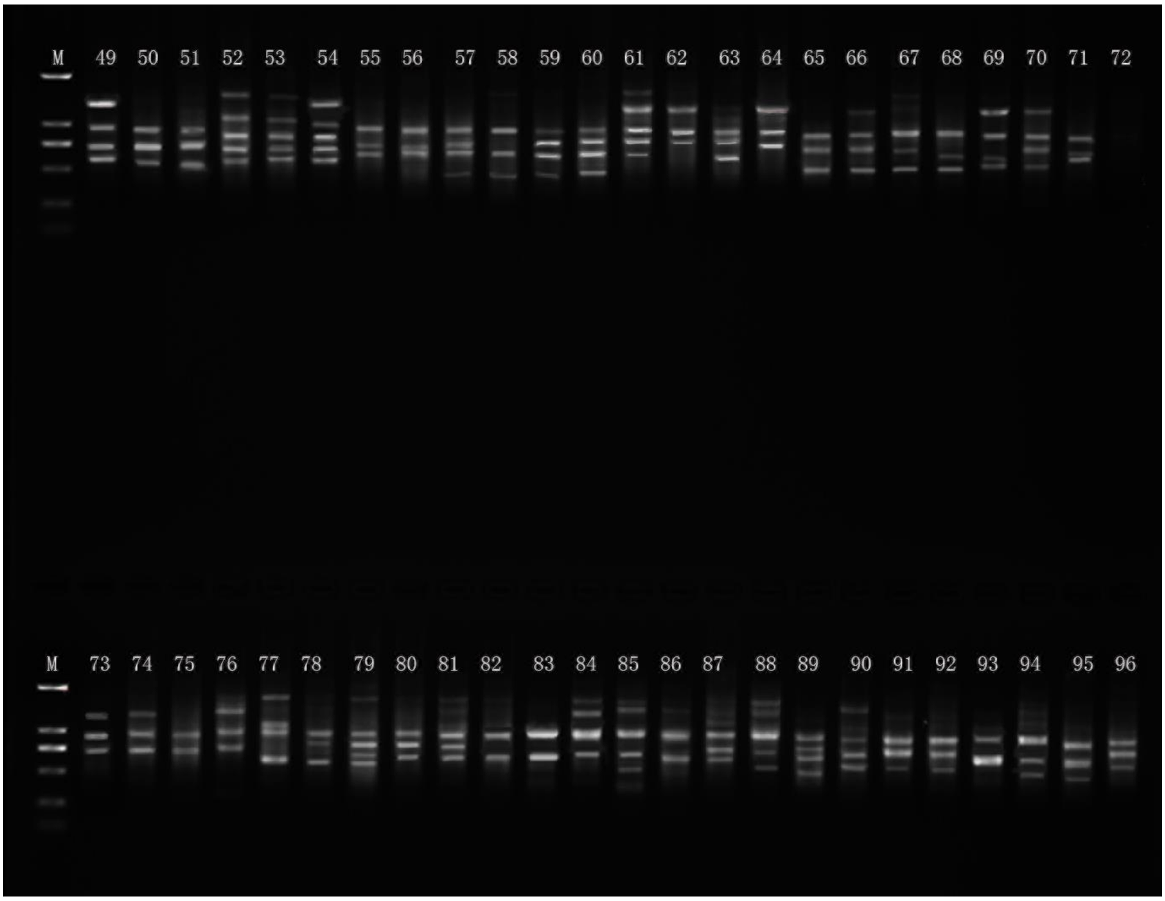

38. Amplification effect of primer ScoT 20 on *A.sinesis* samples (Voucher No. AS 49-96)

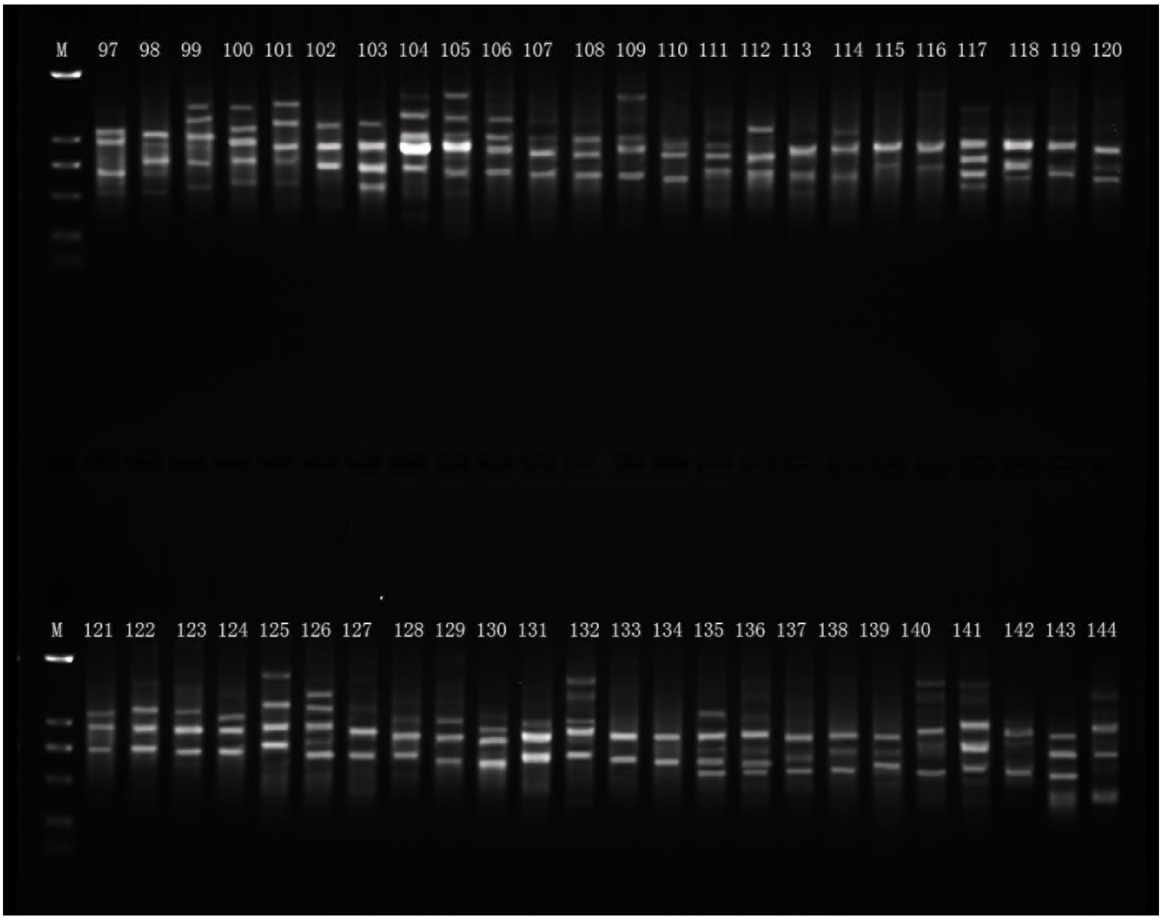

39. Amplification effect of primer ScoT 20 on *A.sinesis* samples (Voucher No. AS 97-144)

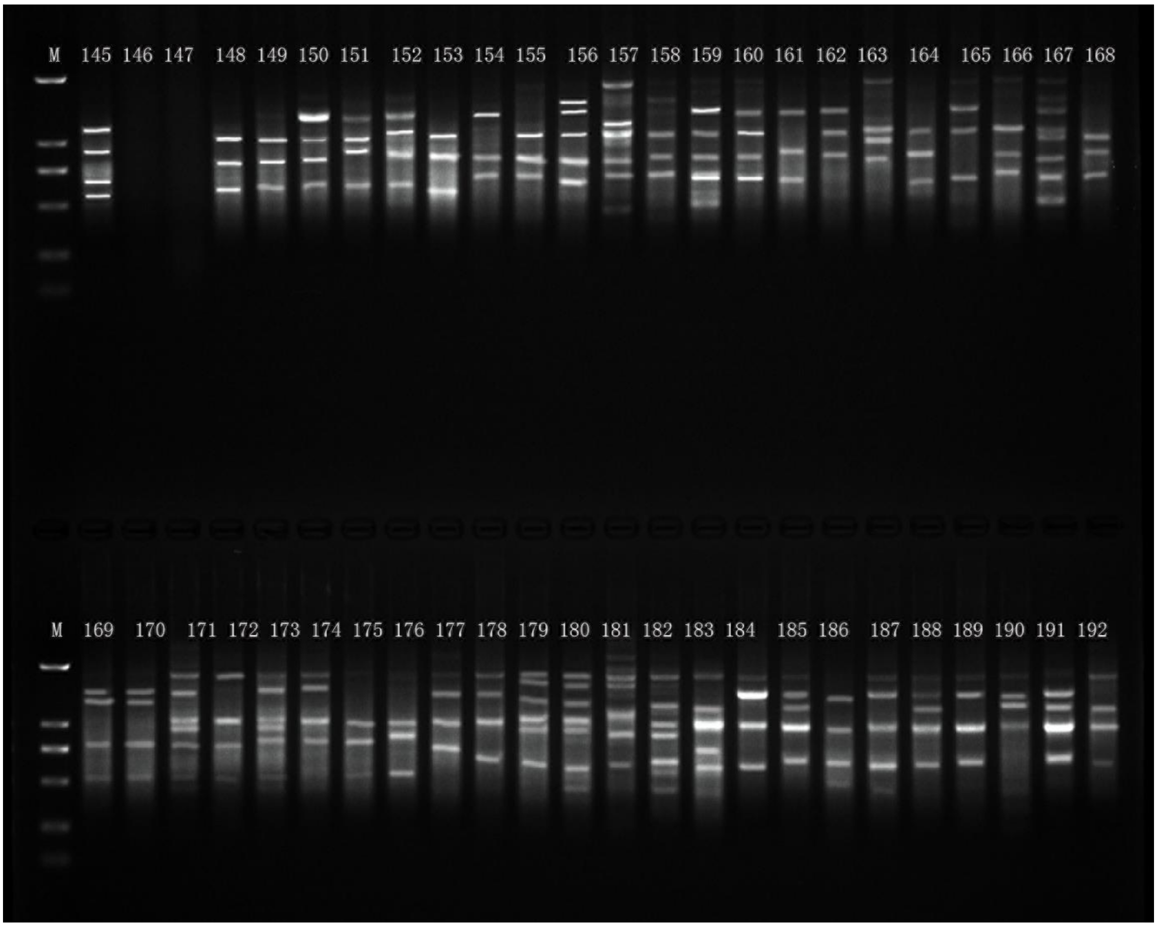

40. Amplification effect of primer ScoT 20 on *A.sinesis* samples (Voucher No. AS 145-192)

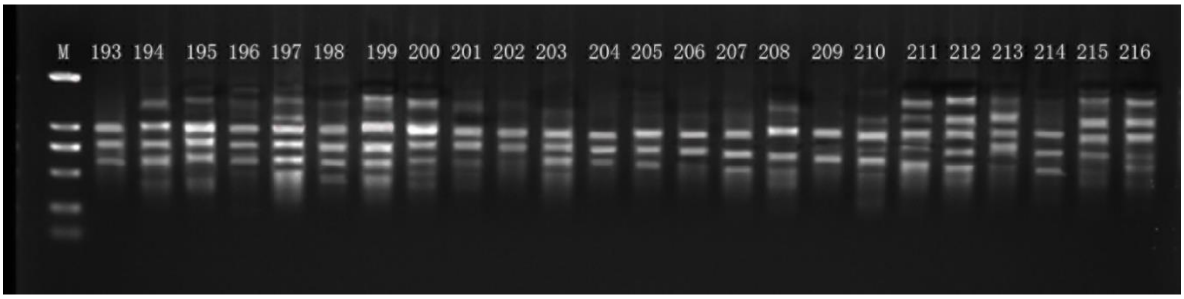

41. Amplification effect of primer ScoT 20 on *A.sinesis* samples (Voucher No. AS 193-216)

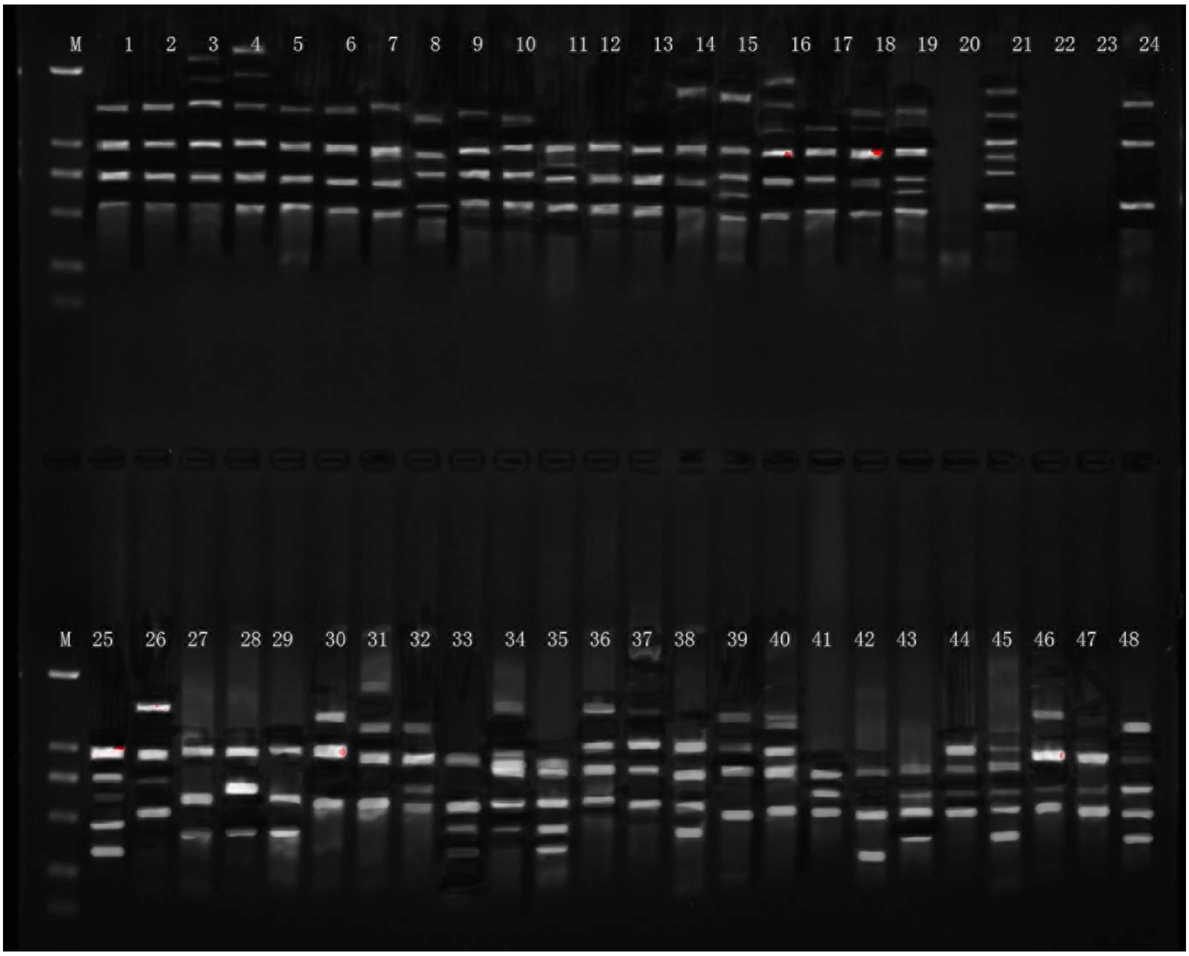

42. Amplification effect of primer ScoT 21 on *A.sinesis* samples (Voucher No. AS 1- 48)

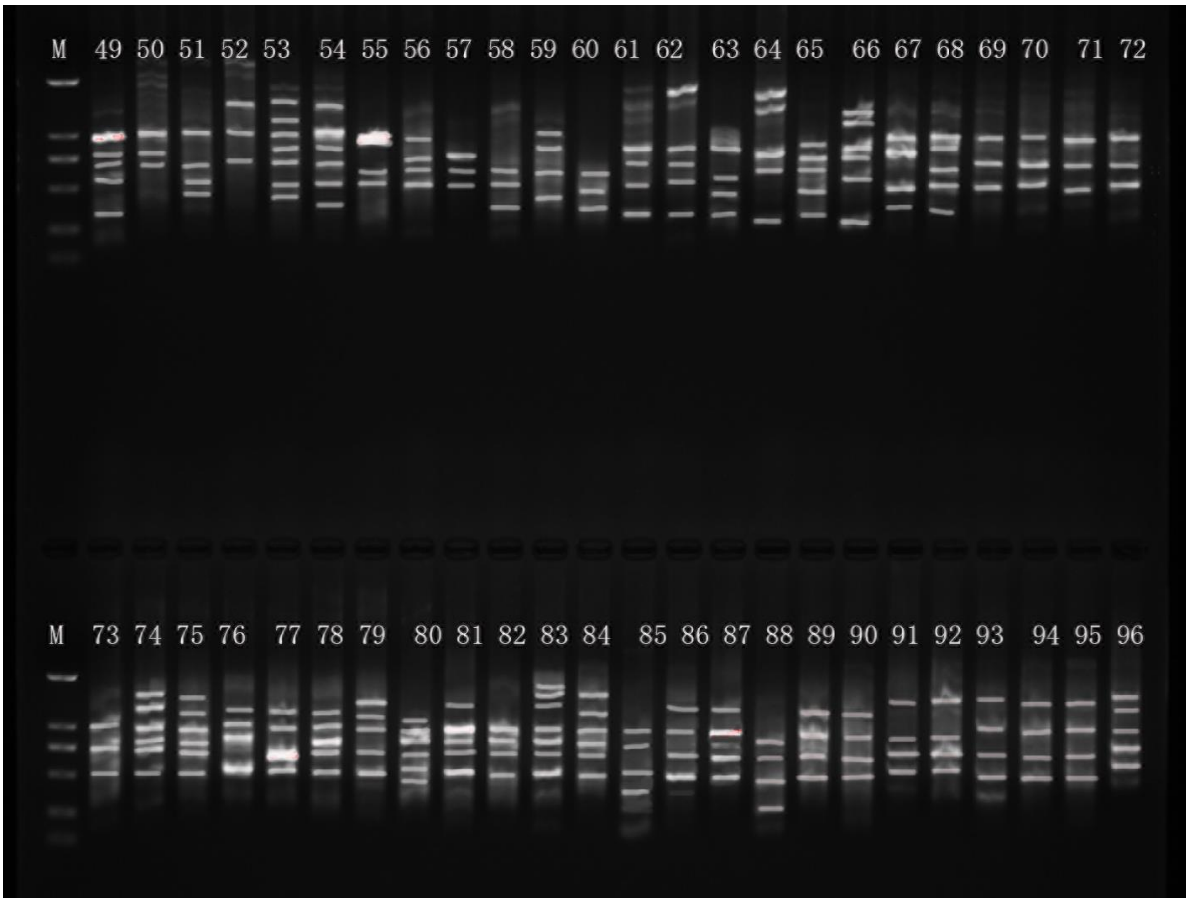

43. Amplification effect of primer ScoT 21 on *A.sinesis* samples (Voucher No. AS 49- 96)

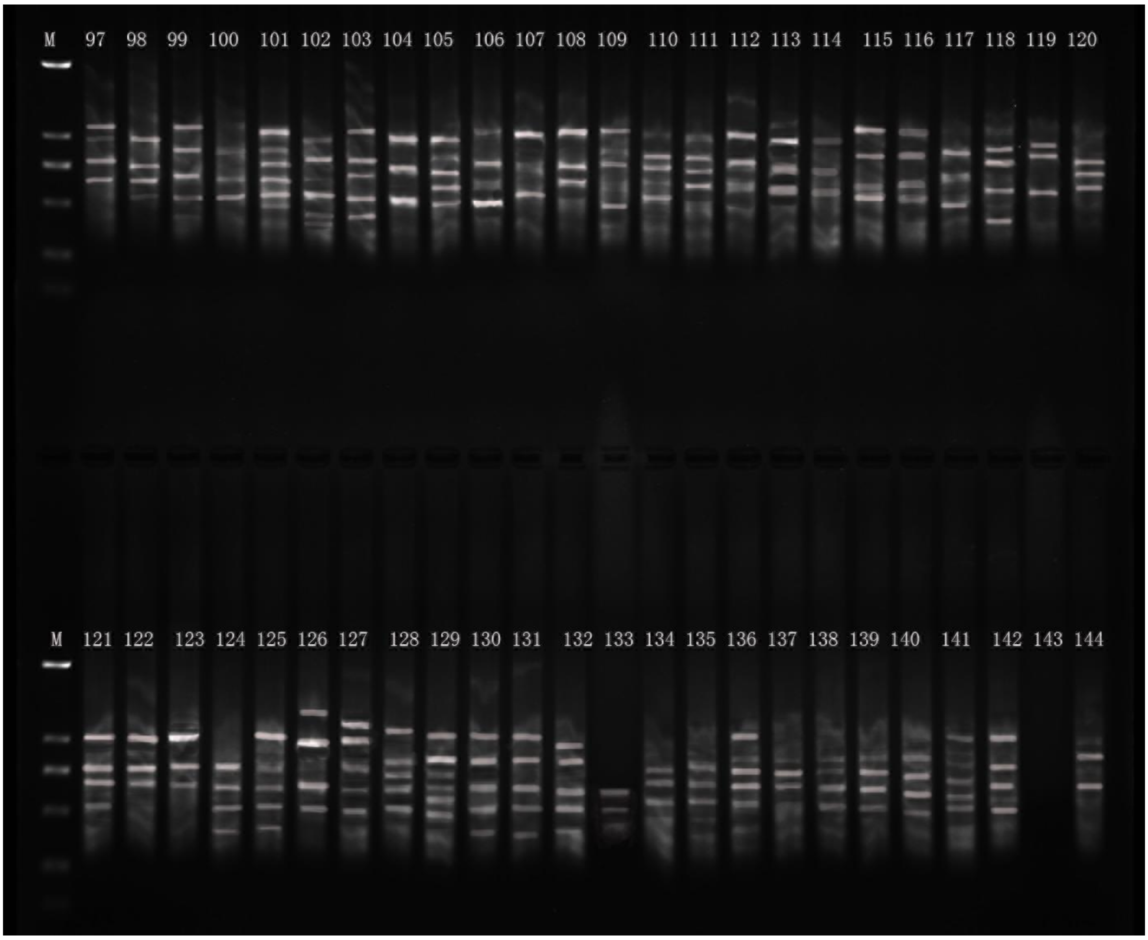

44. Amplification effect of primer ScoT 21 on *A.sinesis* samples (Voucher No. AS 97- 144)

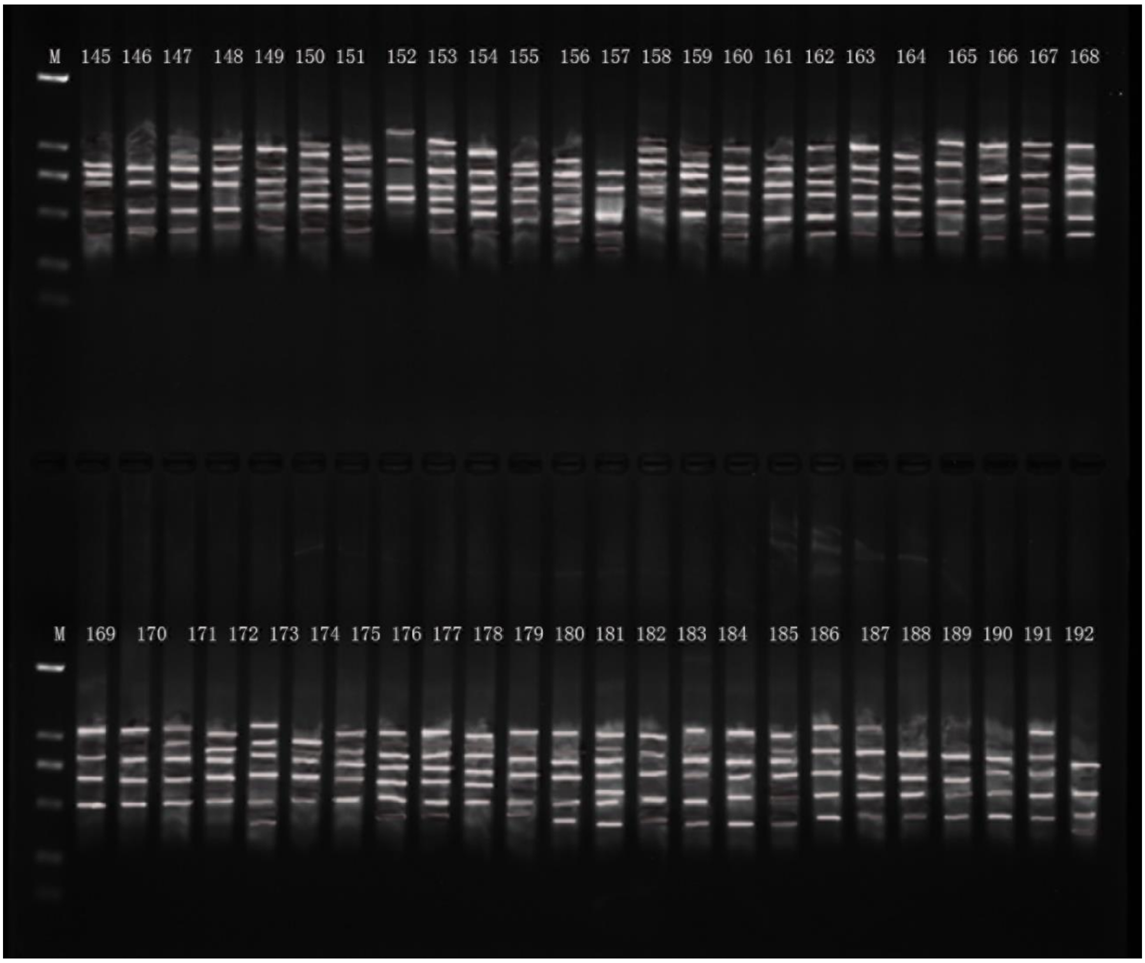

45. Amplification effect of primer ScoT 21 on *A.sinesis* samples (Voucher No. AS 145- 192)

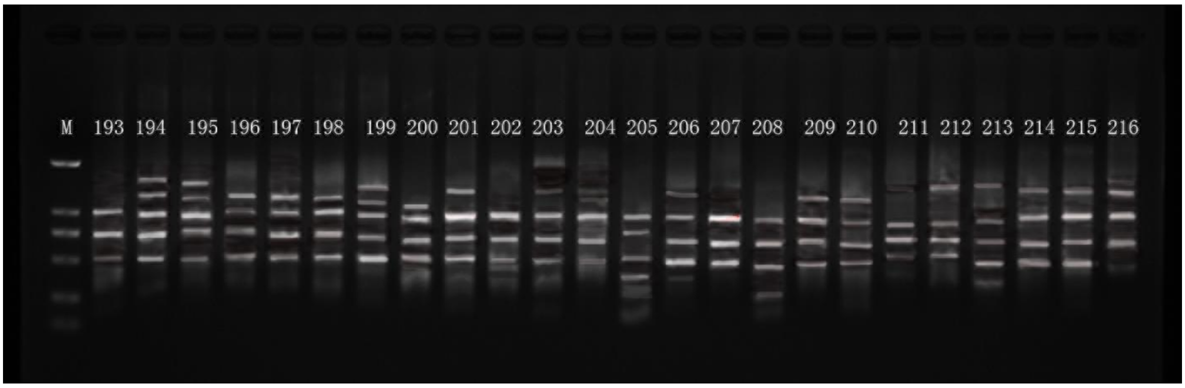

46. Amplification effect of primer ScoT 21 on *A.sinesis* samples (Voucher No. AS 193- 216)

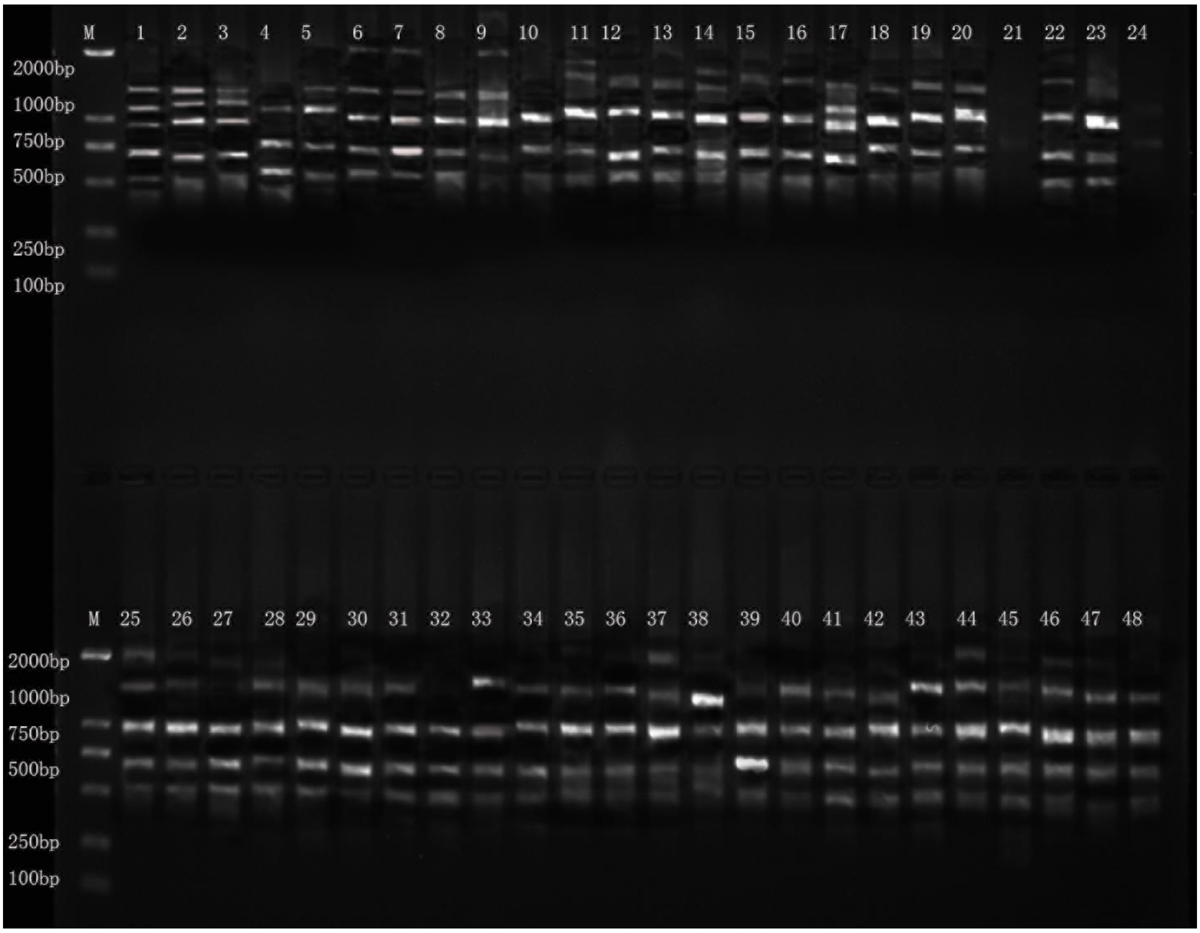

47. Amplification effect of primer ScoT 23 on *A.sinesis* samples (Voucher No. AS 1 - 48)

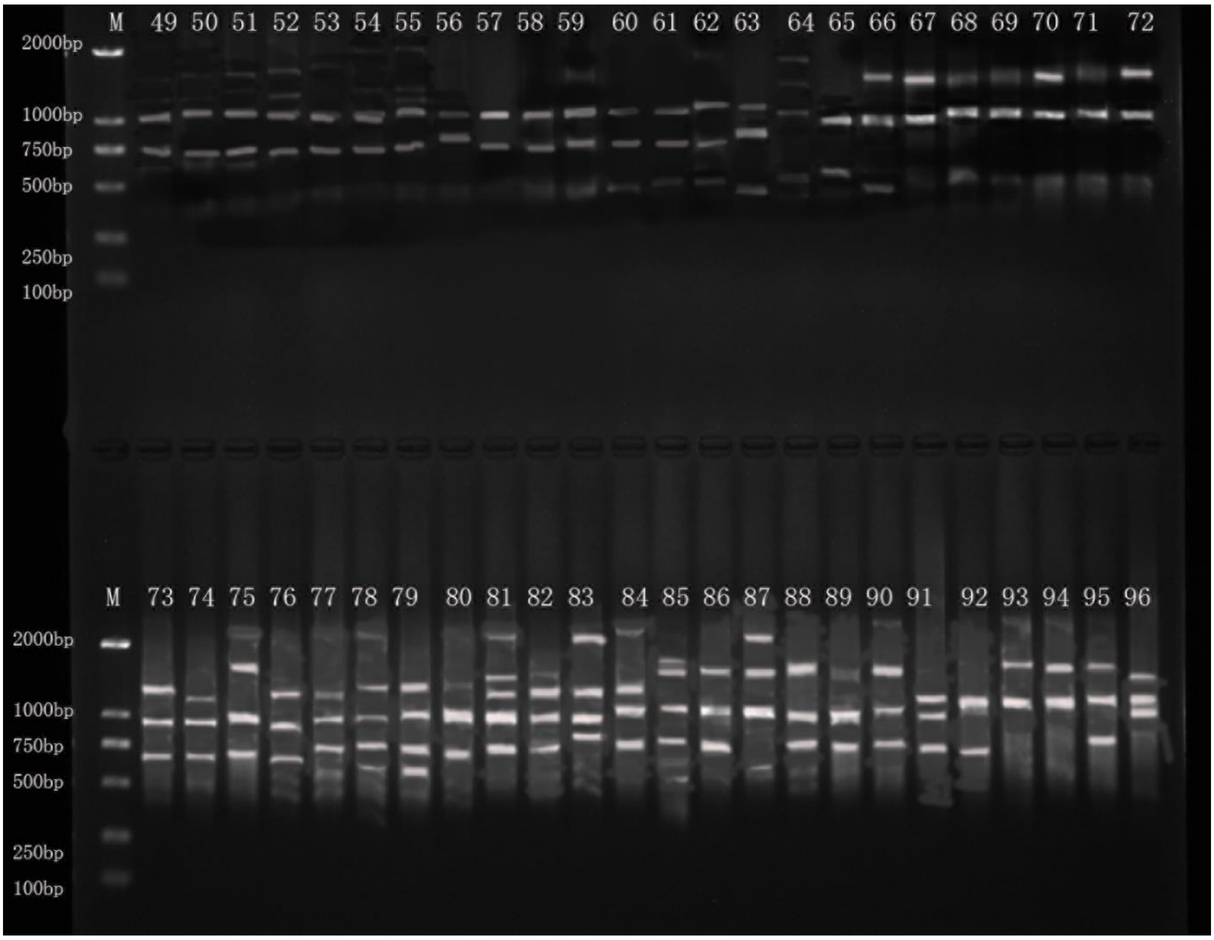

48. Amplification effect of primer ScoT 23 on *A.sinesis* samples (Voucher No. AS 49 - 96)

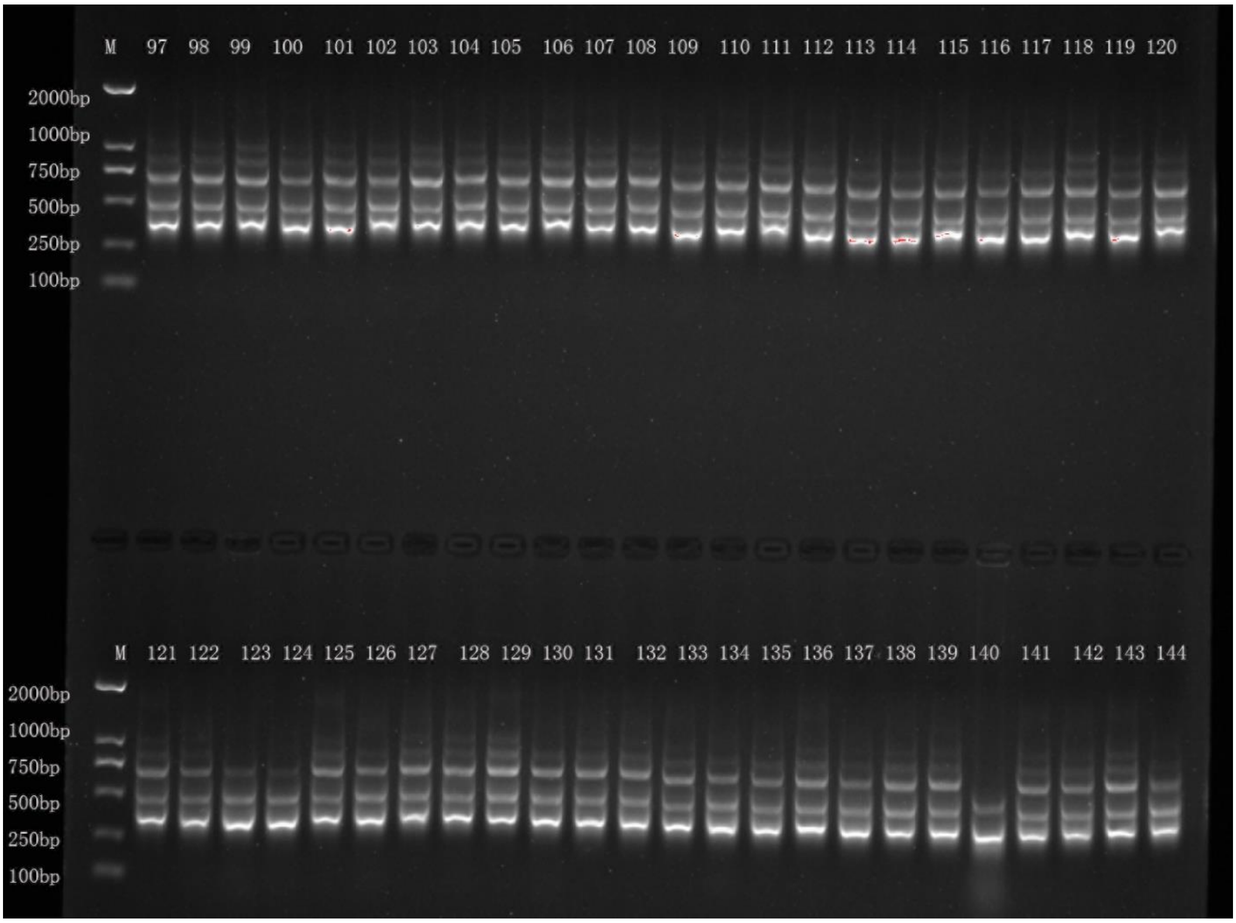

49. Amplification effect of primer ScoT 23 on *A.sinesis* samples (Voucher No. AS 97 - 144)

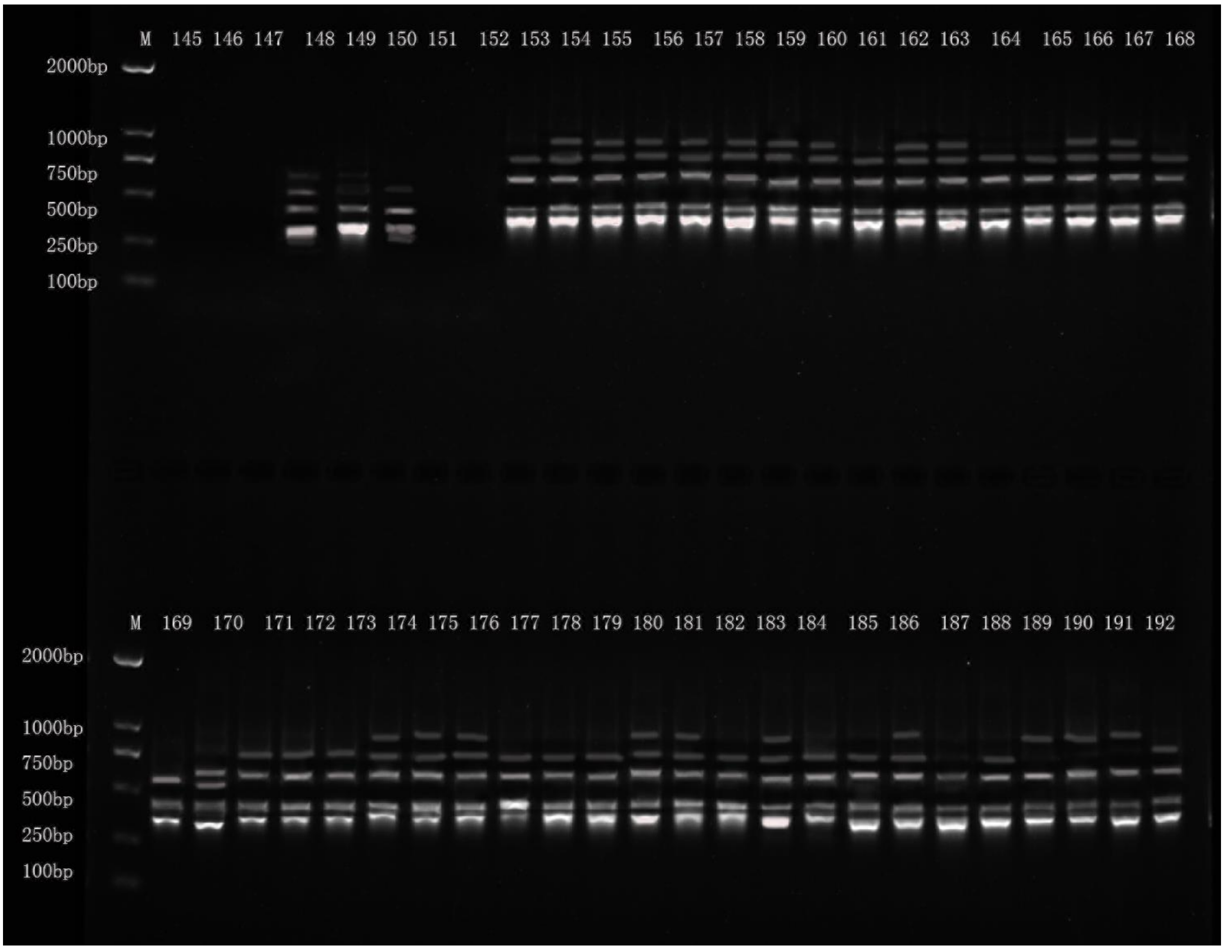

50. Amplification effect of primer ScoT 23 on *A.sinesis* samples (Voucher No. AS 145 - 192)

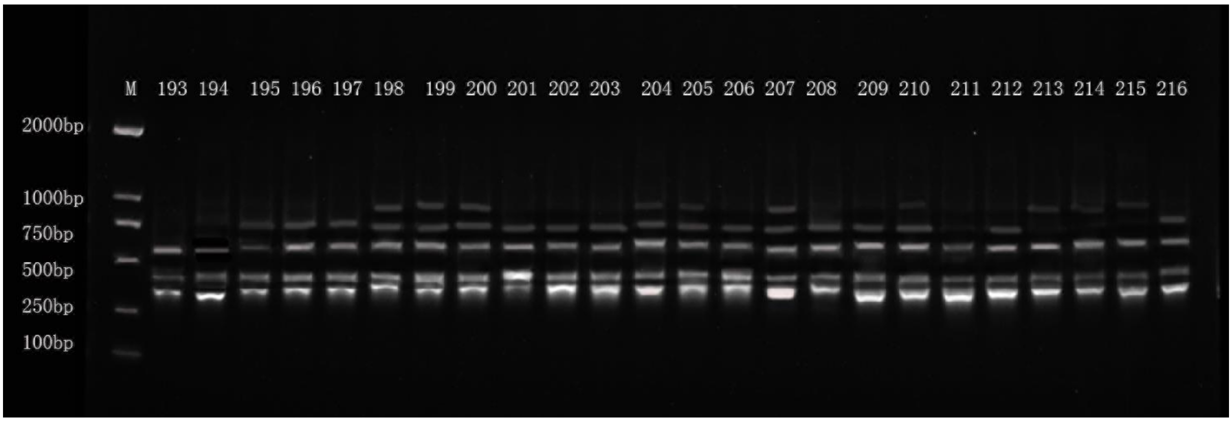

51. Amplification effect of primer ScoT 23 on *A.sinesis* samples (Voucher No. AS 193 - 216)

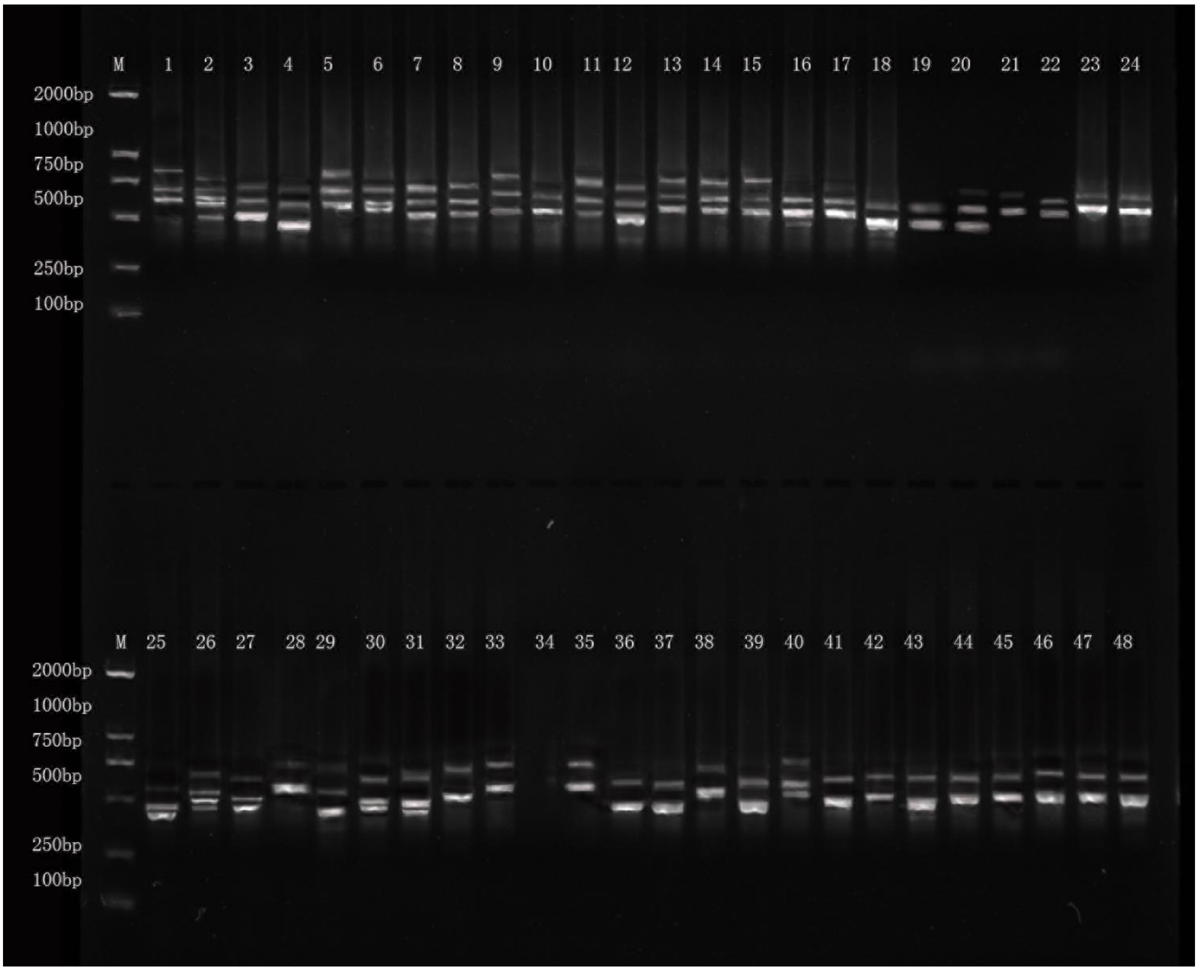

52. Amplification effect of primer ScoT 24 on *A.sinesis* samples (Voucher No. AS 1 - 48)

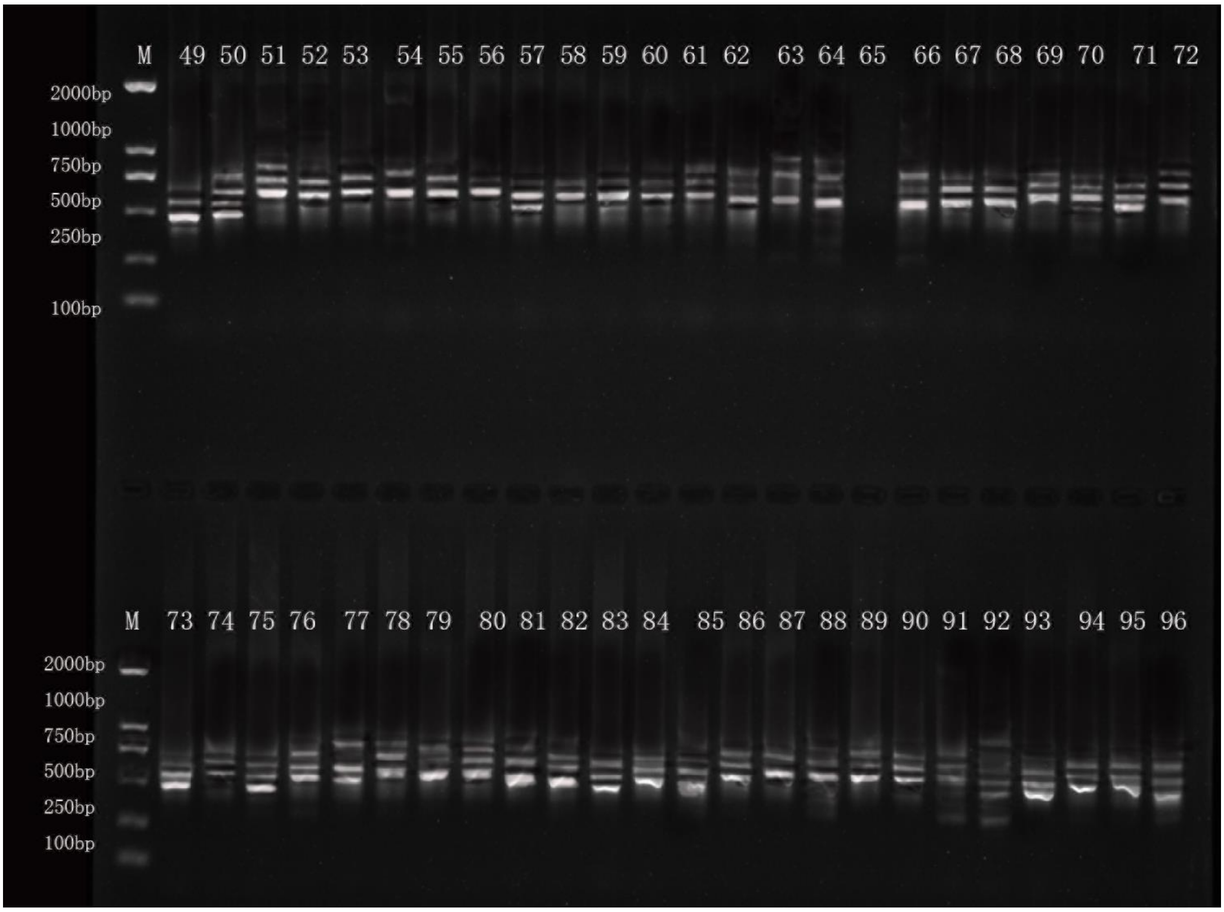

53. Amplification effect of primer ScoT 24 on *A.sinesis* samples (Voucher No. AS 49 - 96)

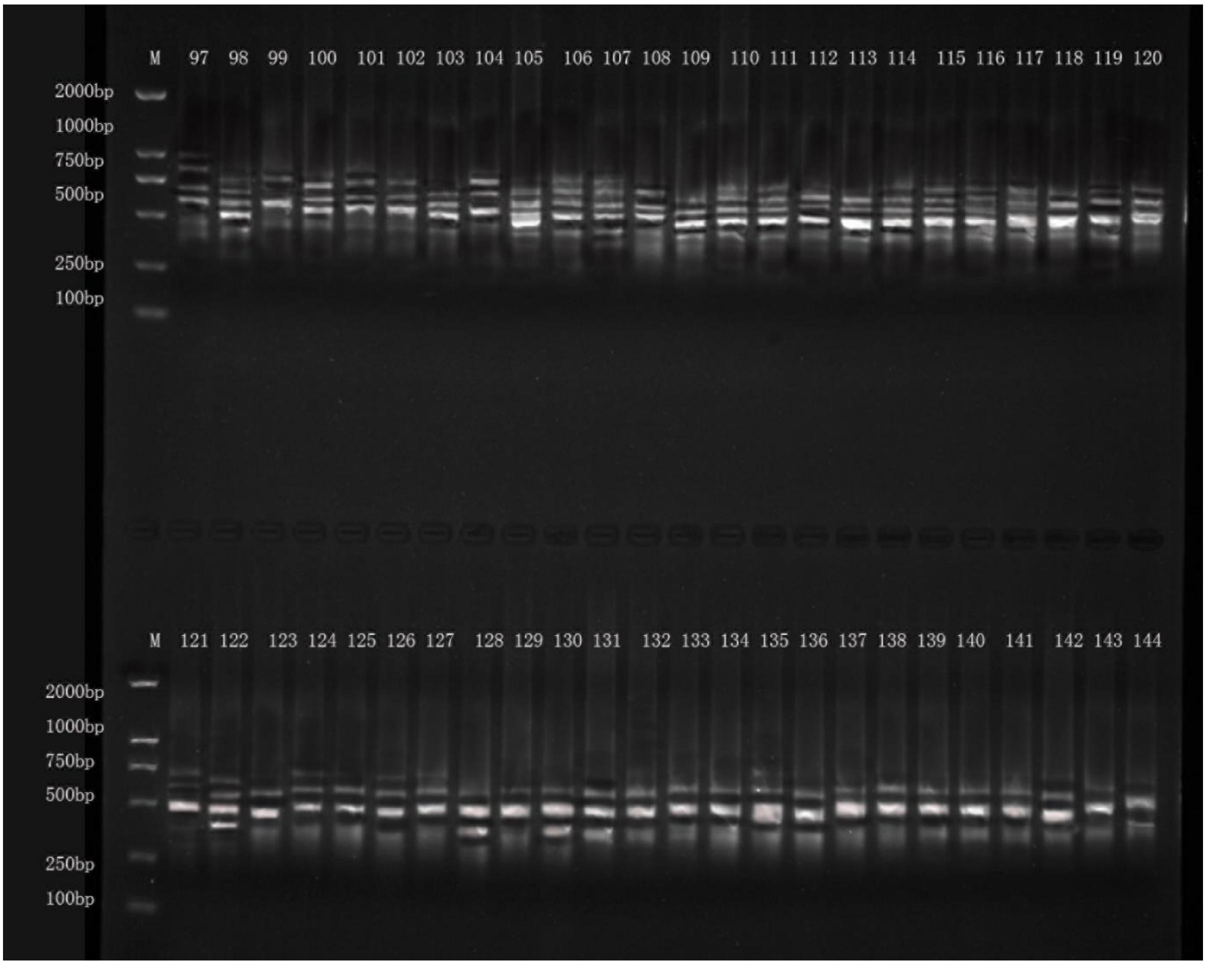

54. Amplification effect of primer ScoT 24 on *A.sinesis* samples (Voucher No. AS 97 - 144)

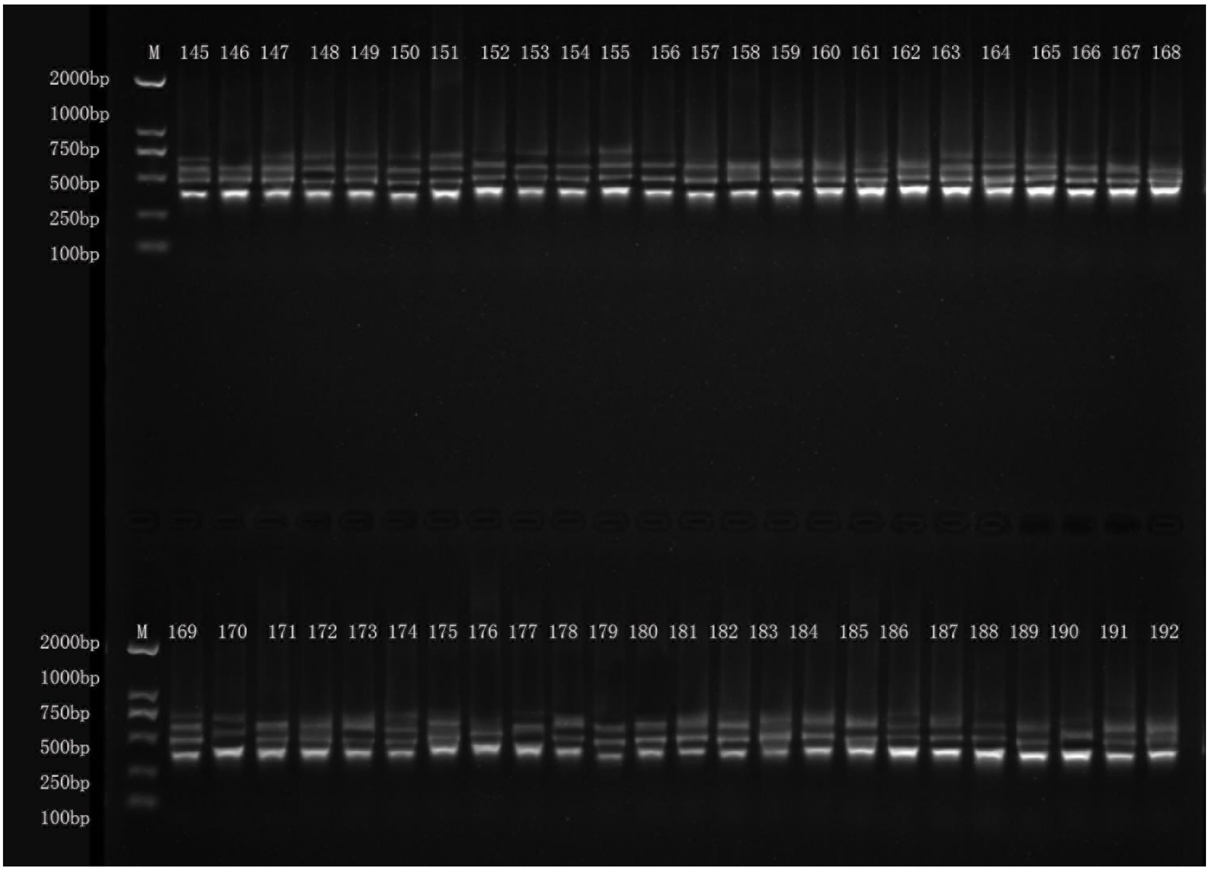

55. Amplification effect of primer ScoT 24 on *A.sinesis* samples (Voucher No. AS 145 - 192)

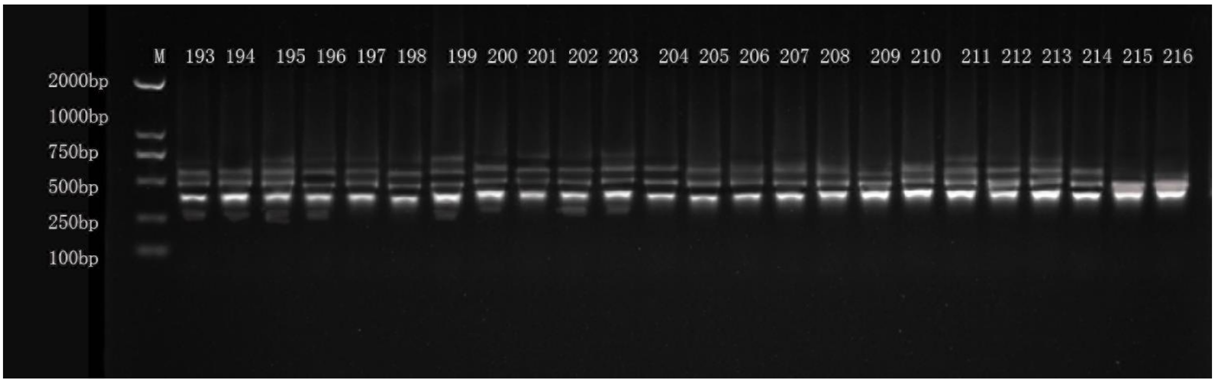

56. Amplification effect of primer ScoT 24 on *A.sinesis* samples (Voucher No. AS 193 - 216)

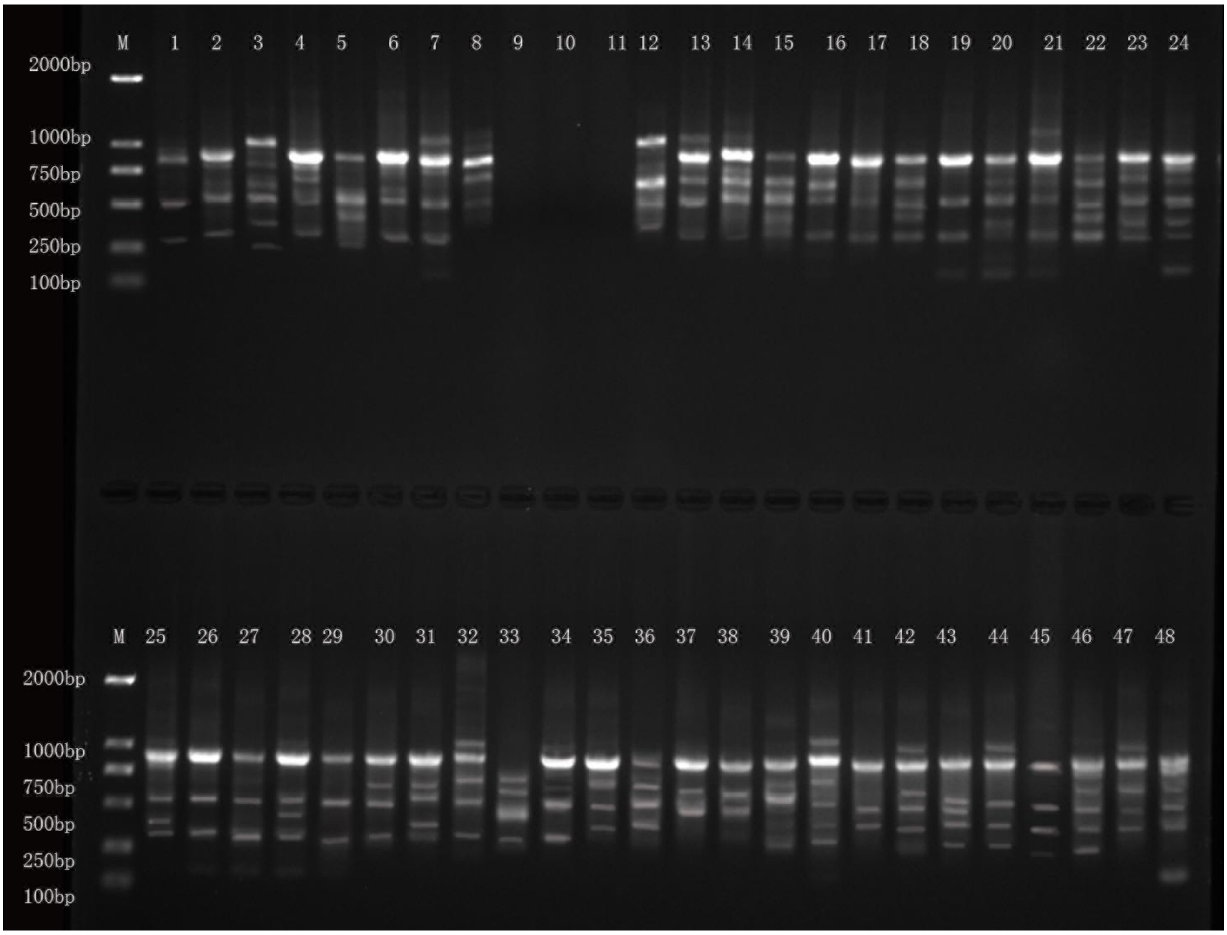

57. Amplification effect of primer ScoT 31 on *A.sinesis* samples (Voucher No. AS 1 - 48)

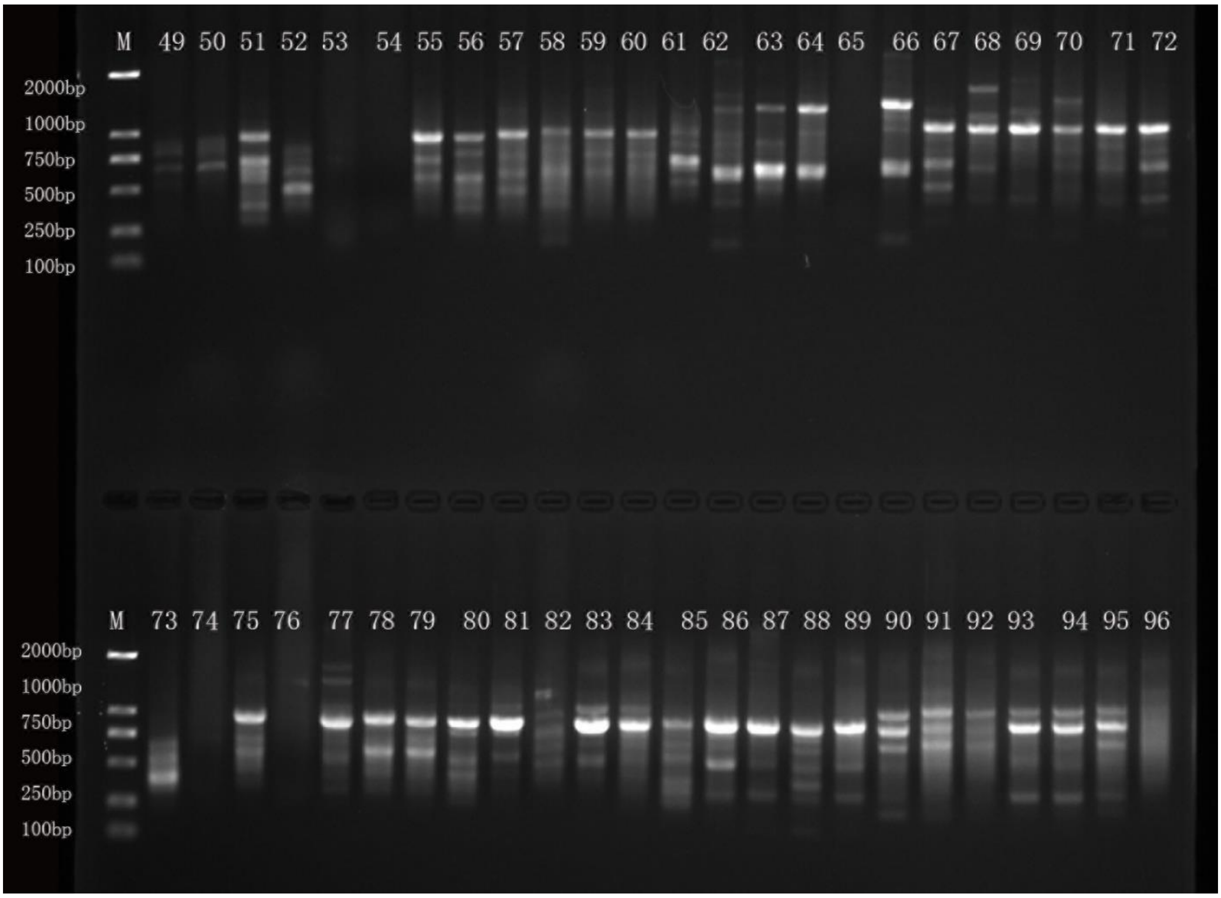

58. Amplification effect of primer ScoT 31 on *A.sinesis* samples (Voucher No. AS 49 - 96)

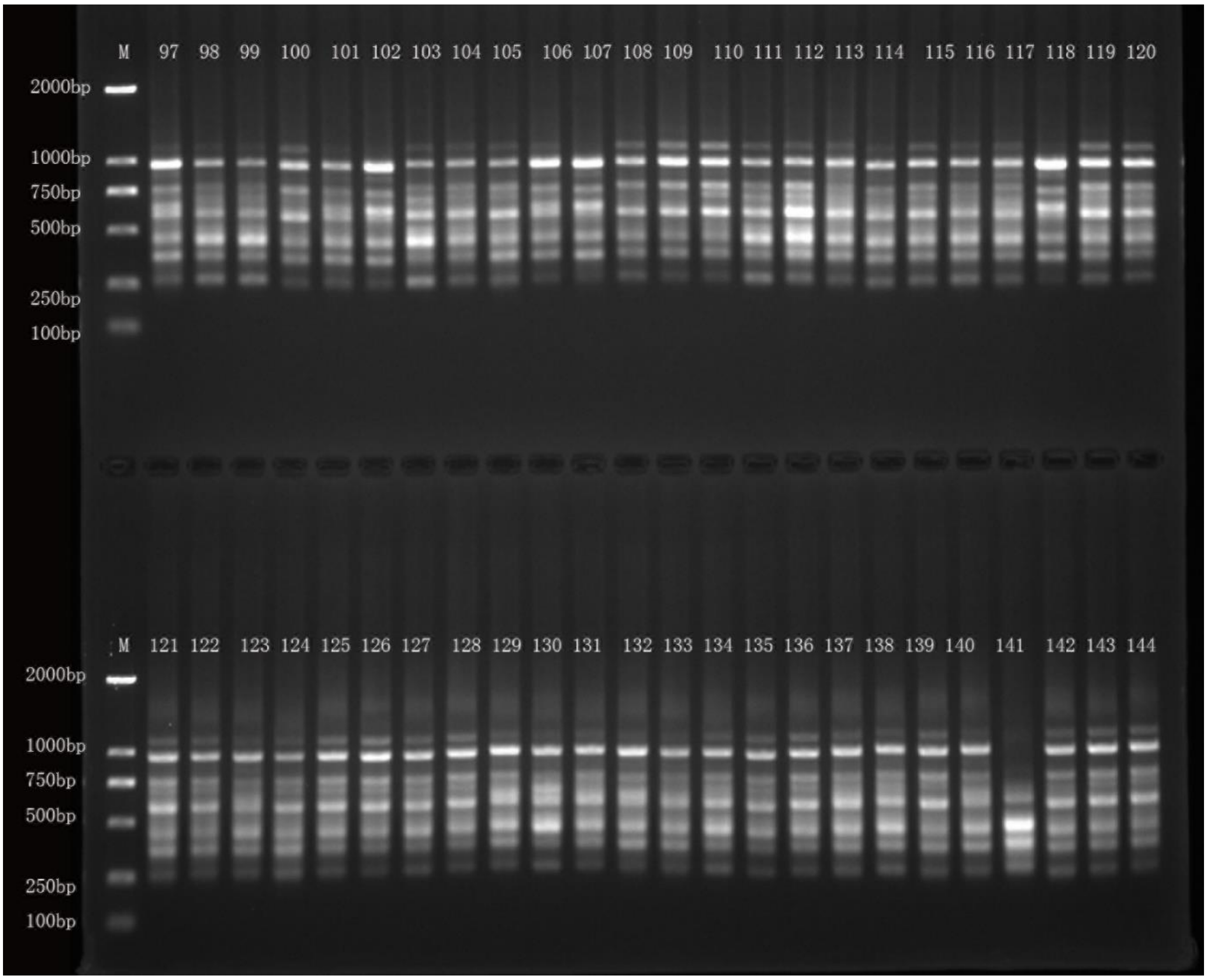

59. Amplification effect of primer ScoT 31 on *A.sinesis* samples (Voucher No. AS 97 - 144)

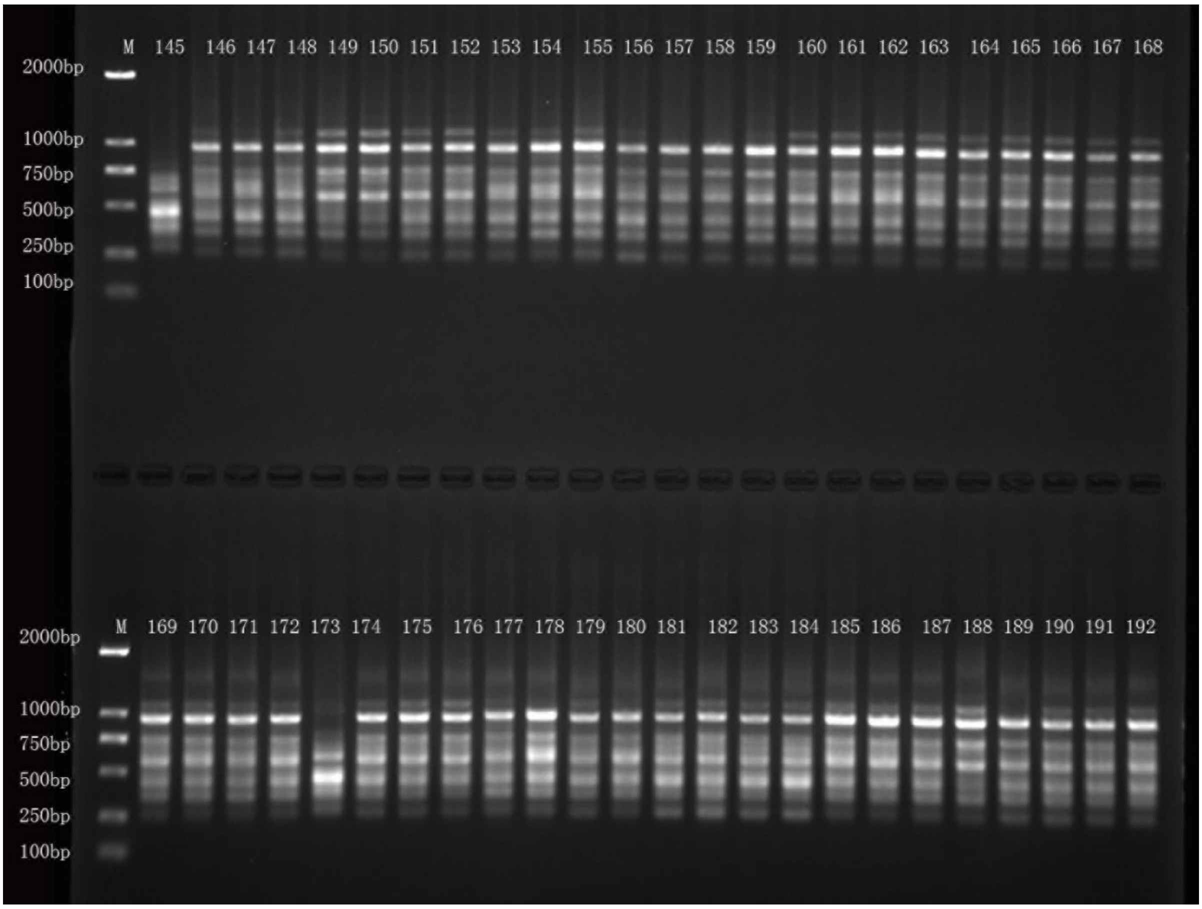

60. Amplification effect of primer ScoT 31 on *A.sinesis* samples (Voucher No. AS 145 - 192)

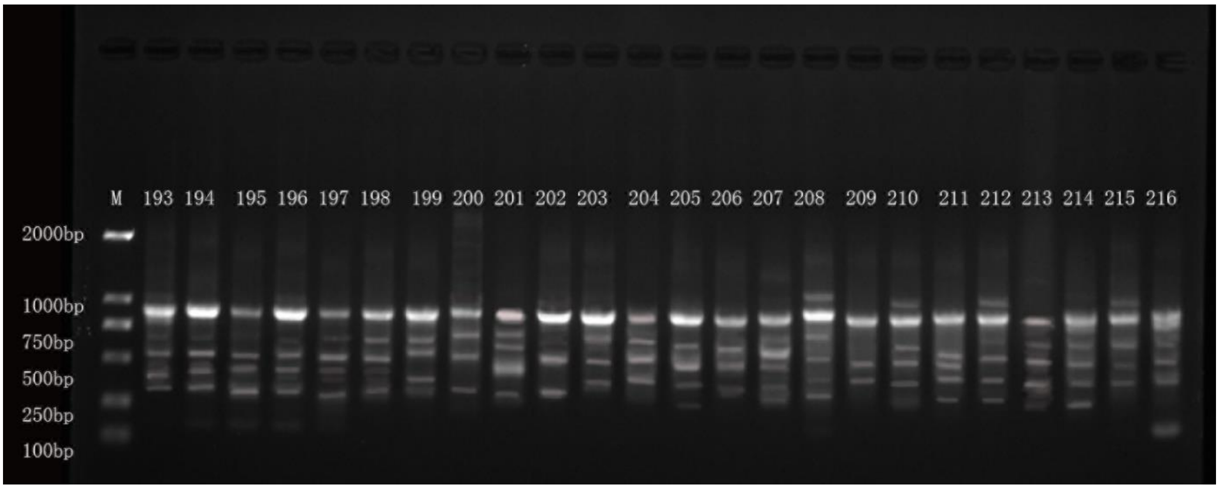

61. Amplification effect of primer ScoT 31 on *A.sinesis* samples (Voucher No. AS 193 - 216)

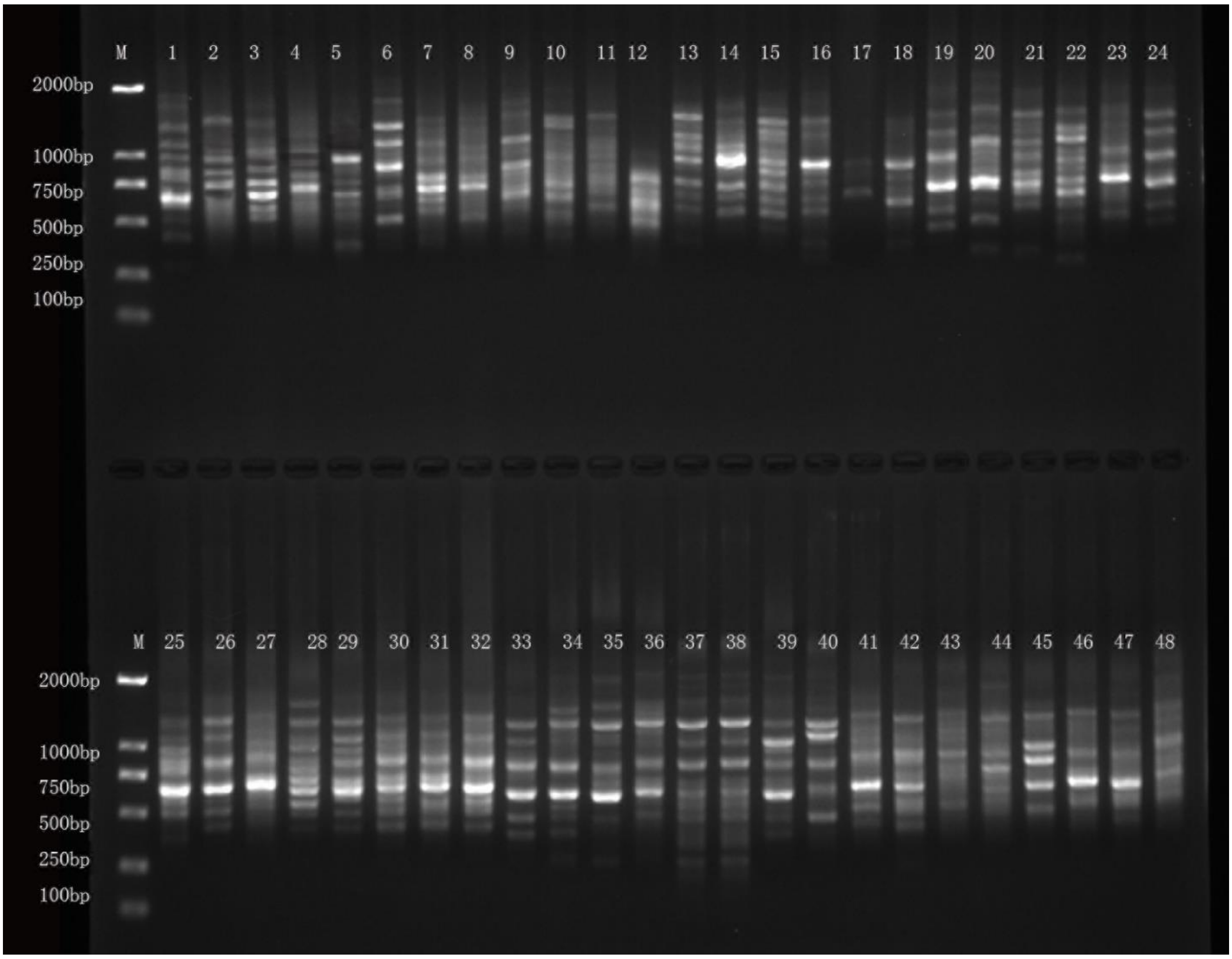

62. Amplification effect of primer ScoT 32 on *A.sinesis* samples (Voucher No. AS 1 - 48)

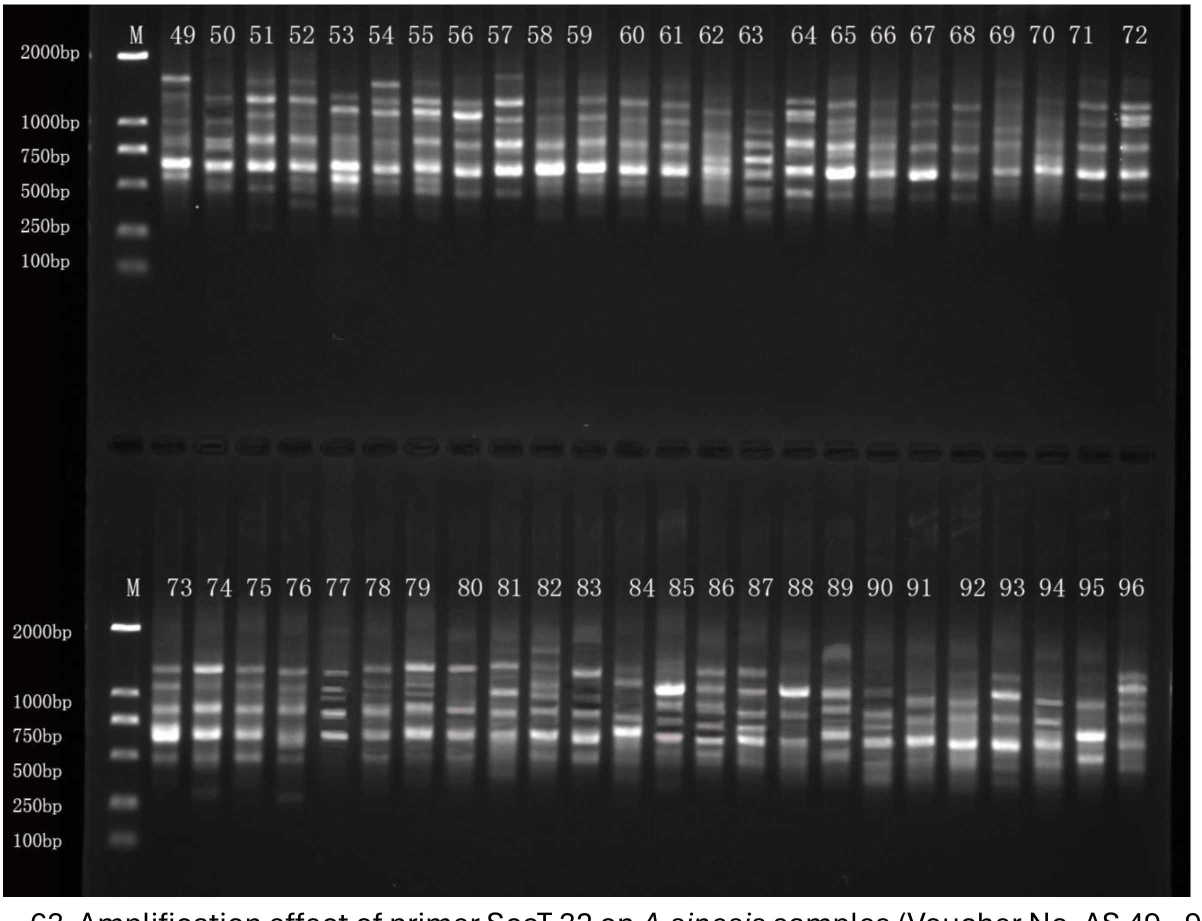

63. Amplification effect of primer ScoT 32 on *A.sinesis* samples (Voucher No. AS 49 - 96)

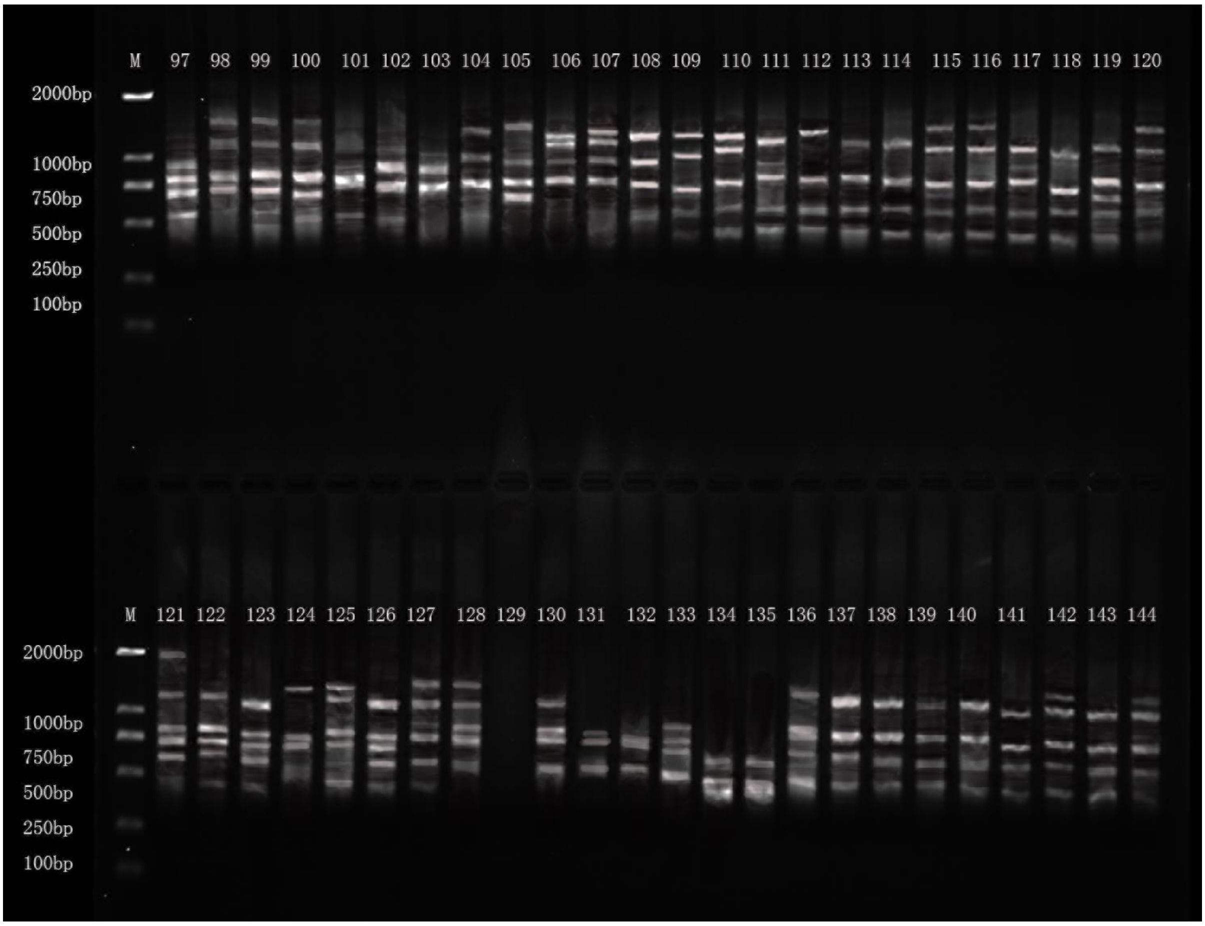

64. Amplification effect of primer ScoT 32 on *A.sinesis* samples (Voucher No. AS 97 - 144)

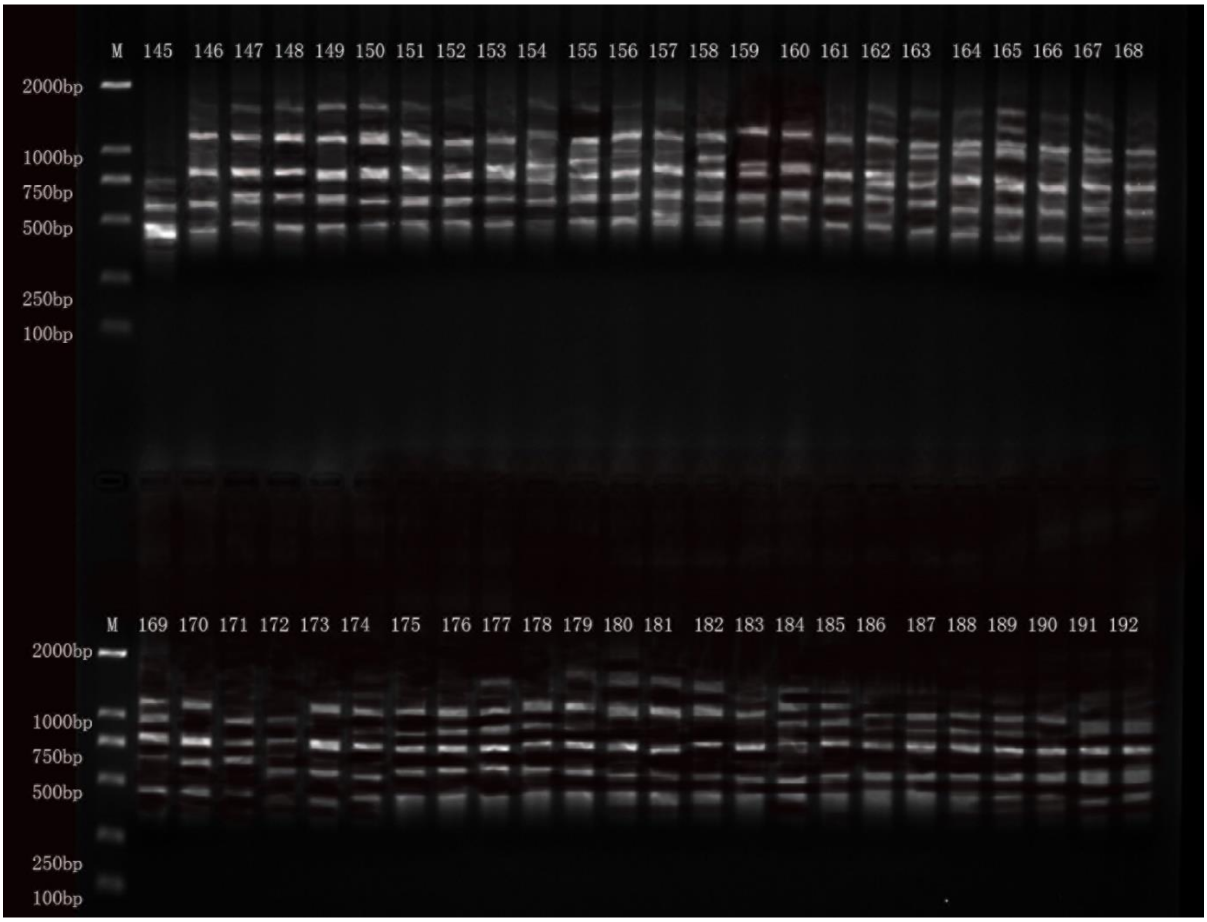

65. Amplification effect of primer ScoT 32 on *A.sinesis* samples (Voucher No. AS 145 - 192)

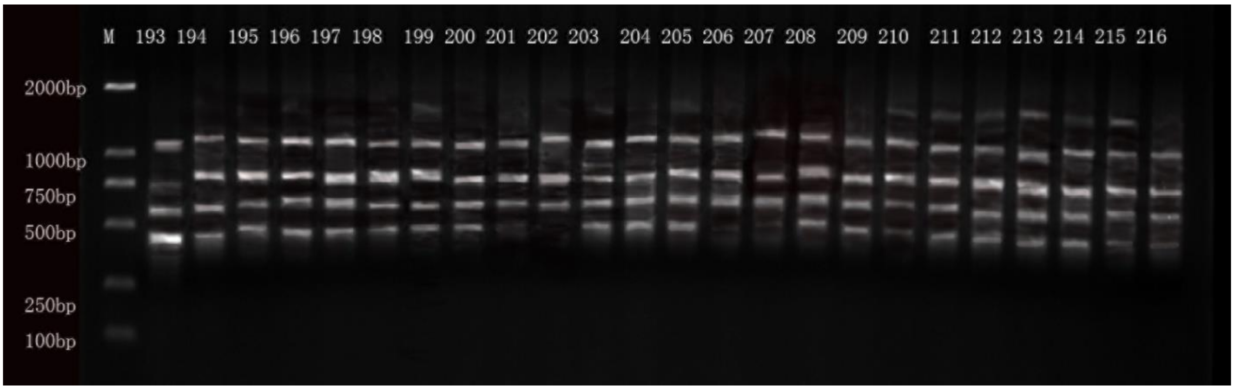

66. Amplification effect of primer ScoT 32 on *A.sinesis* samples (Voucher No. AS 193 - 216)

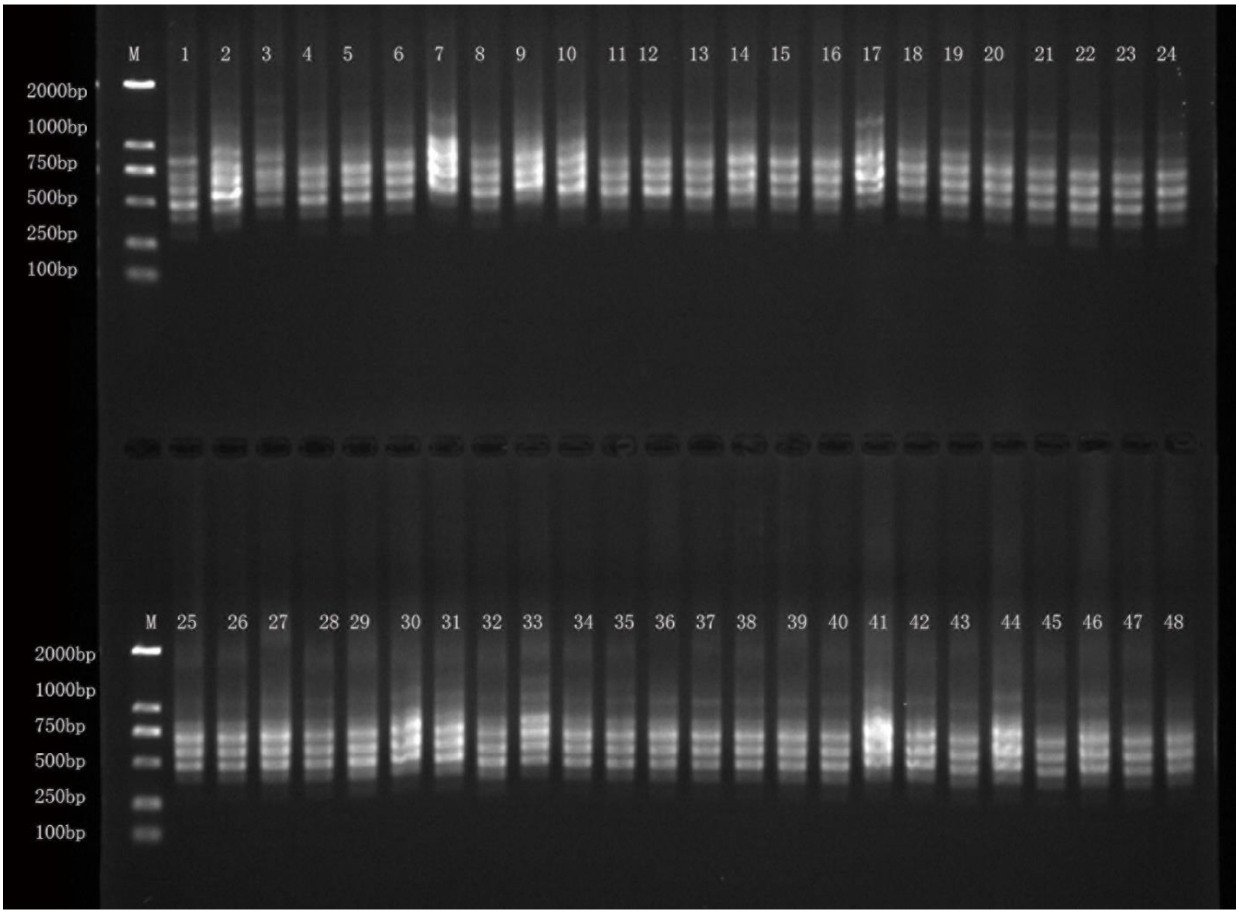

67. Amplification effect of primer ScoT 34 on *A.sinesis* samples (Voucher No. AS 1 - 48)

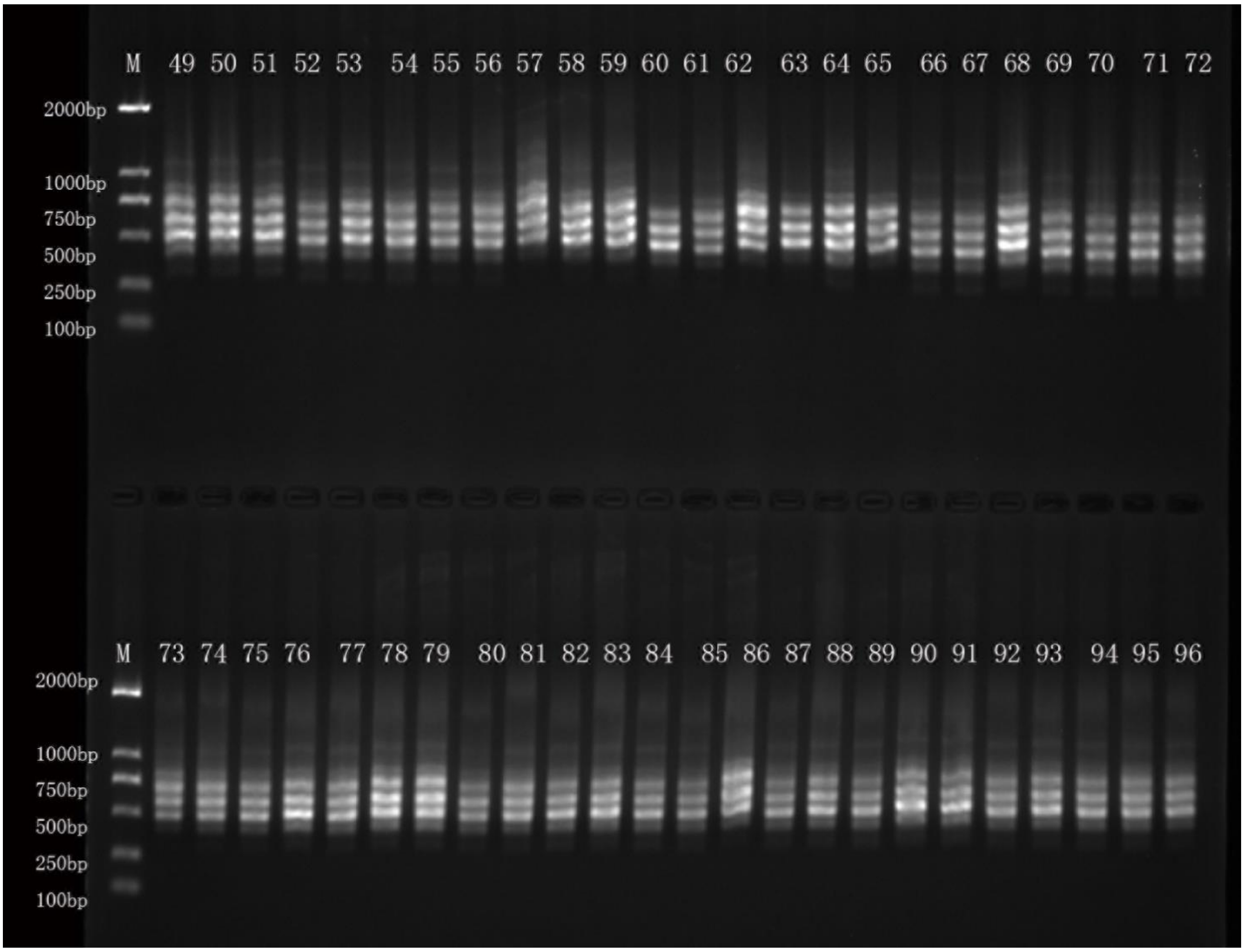

68. Amplification effect of primer ScoT 34 on *A.sinesis* samples (Voucher No. AS 49 - 96)

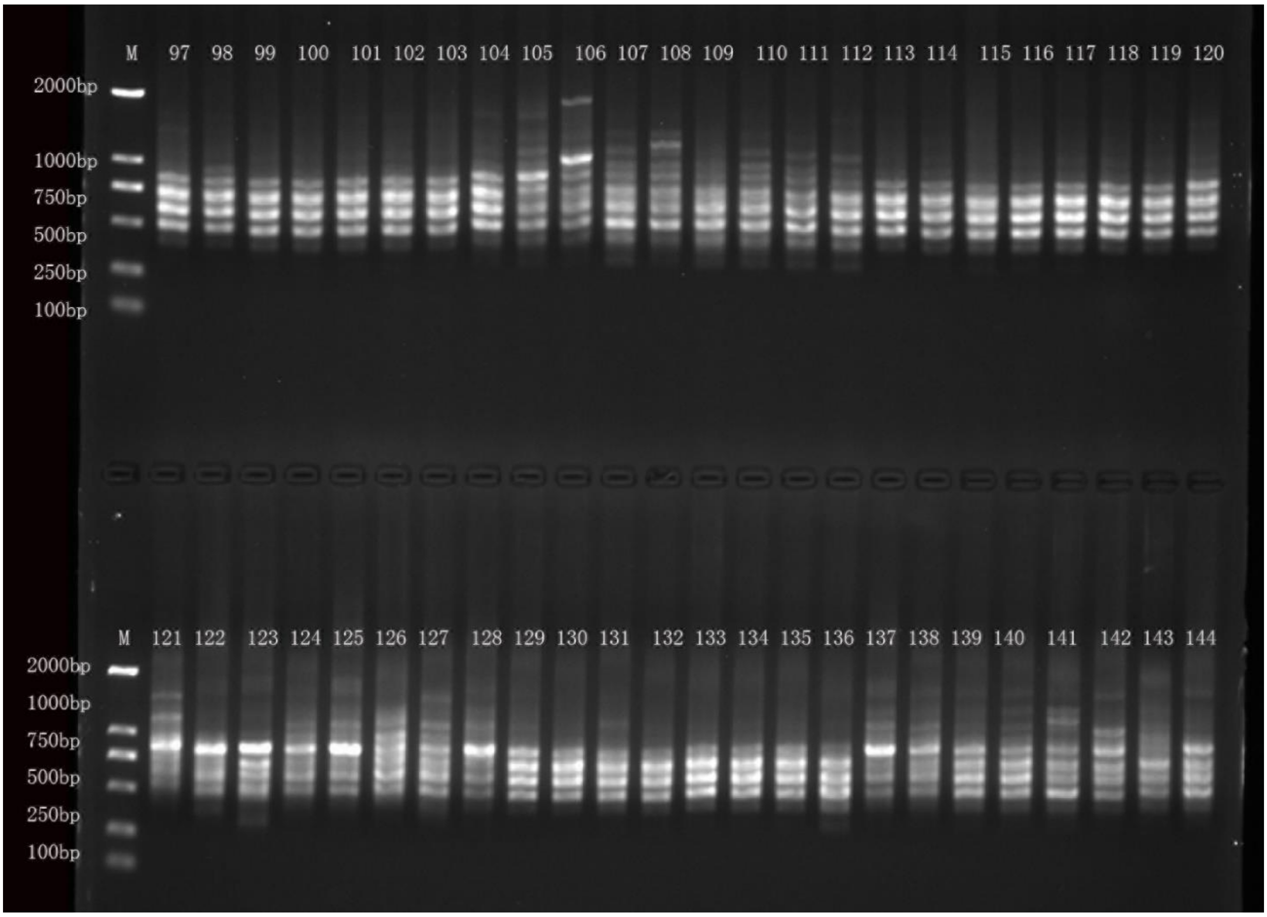

69. Amplification effect of primer ScoT 34 on *A.sinesis* samples (Voucher No. AS 97 - 144)

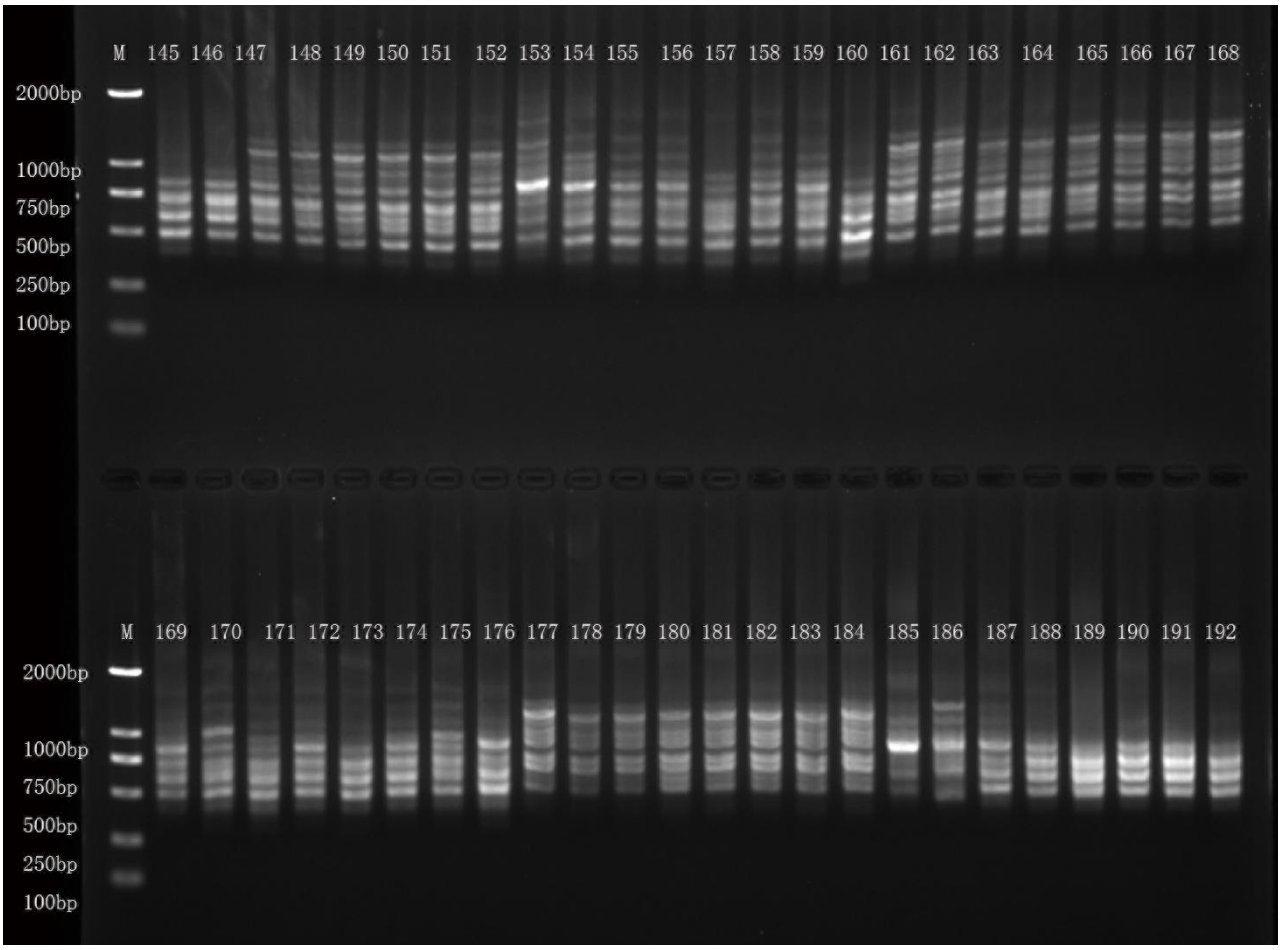

70. Amplification effect of primer ScoT 34 on *A.sinesis* samples (Voucher No. AS 145 - 192)

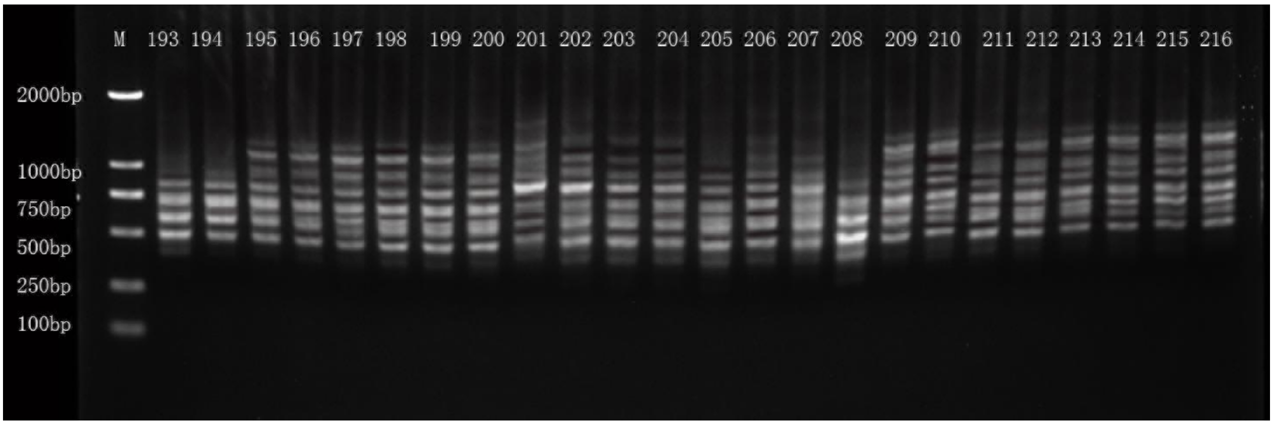

71. Amplification effect of primer ScoT 34 on A.sinesis samples (Voucher No. AS 193 - 216)

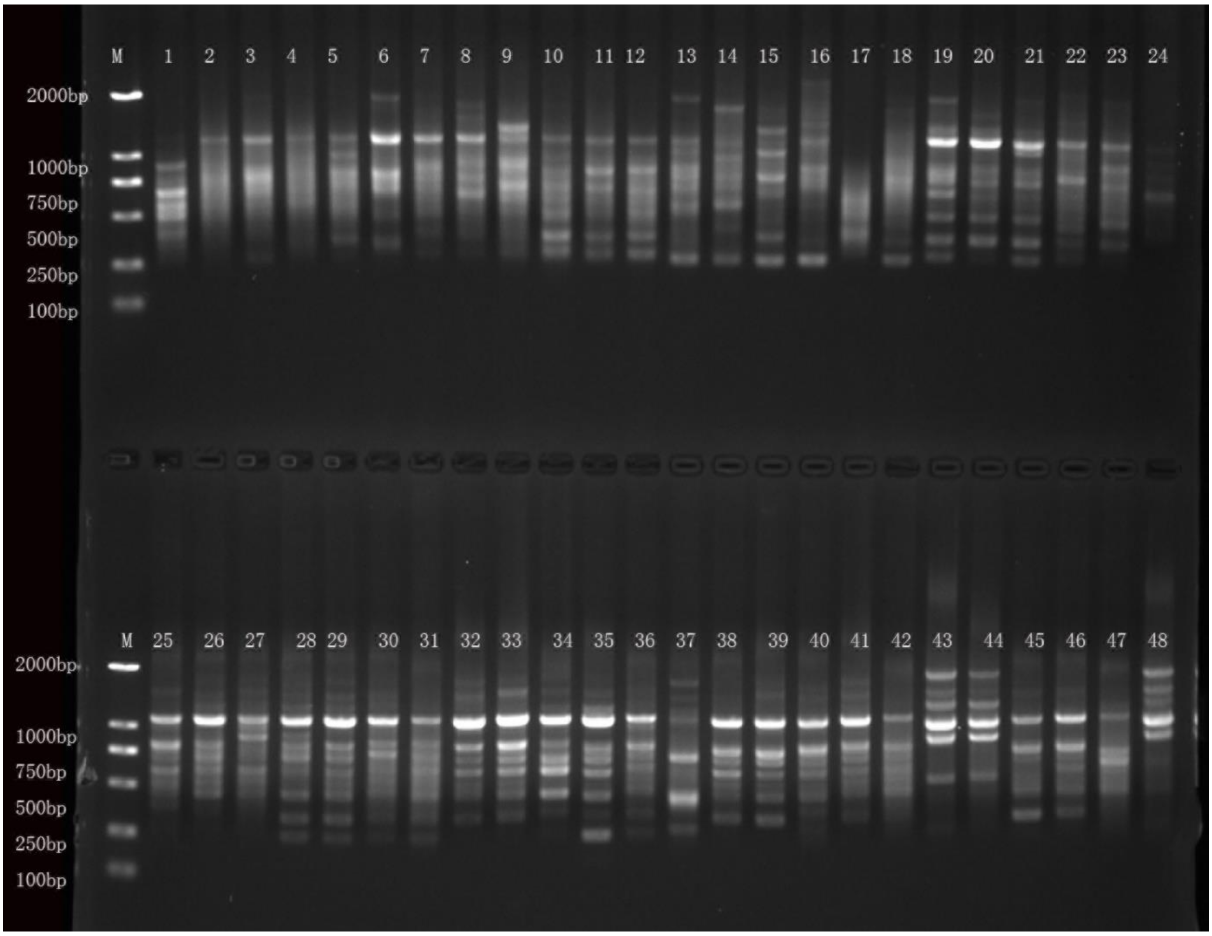

72. Amplification effect of primer ScoT 35 on A.sinesis samples (Voucher No. AS 1 - 48)

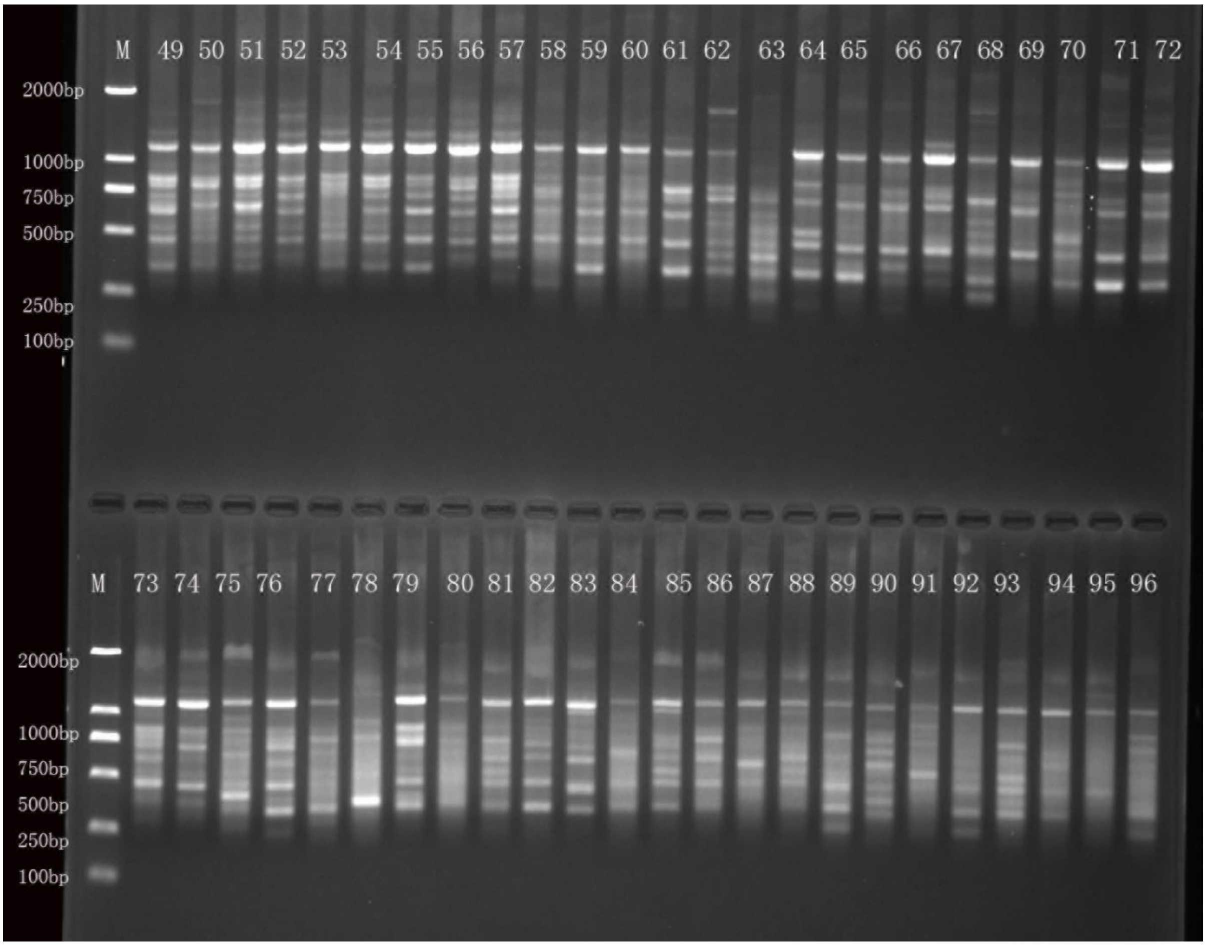

73. Amplification effect of primer ScoT 35 on *A.sinesis* samples (Voucher No. AS 49 - 96)

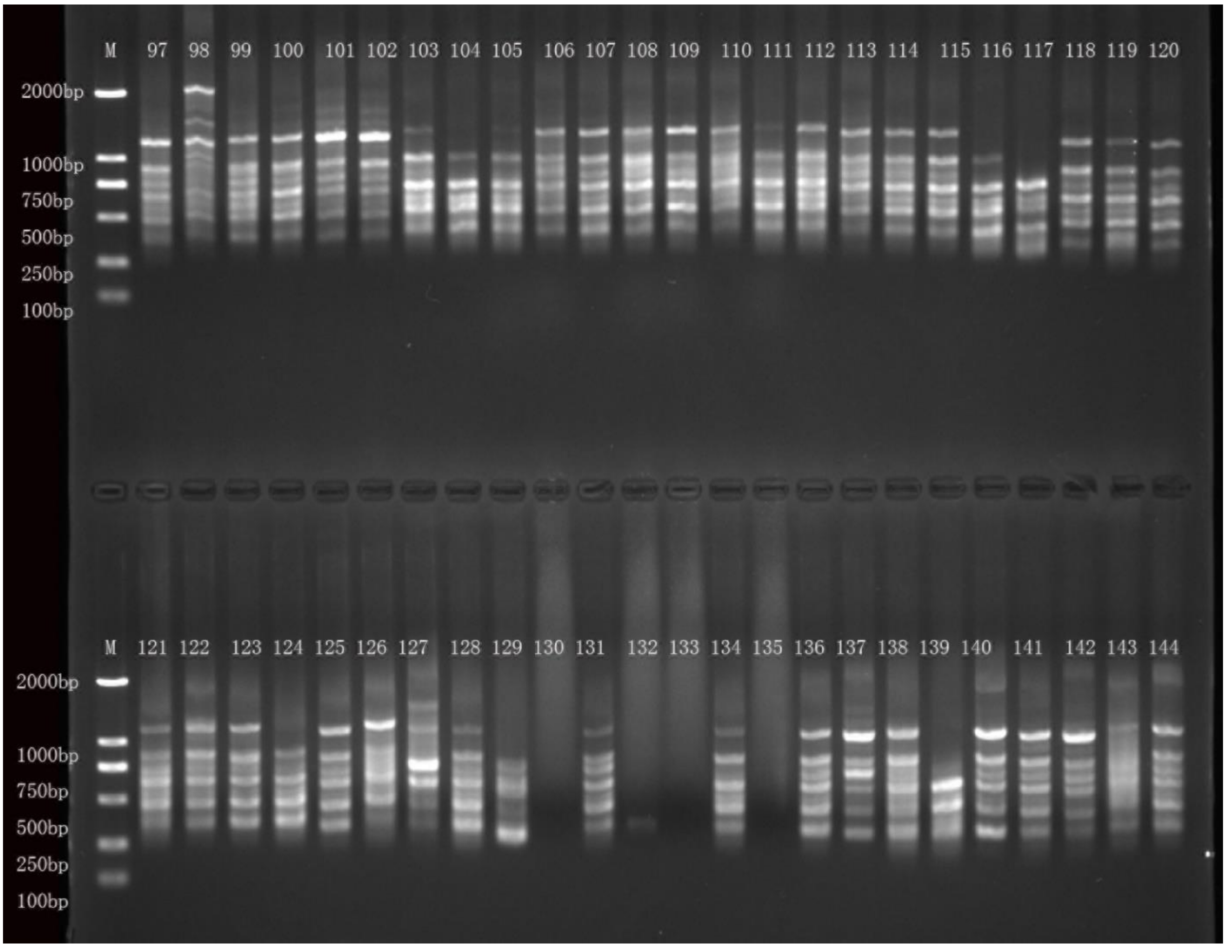

74. Amplification effect of primer ScoT 35 on *A.sinesis* samples (Voucher No. AS 97 - 144)

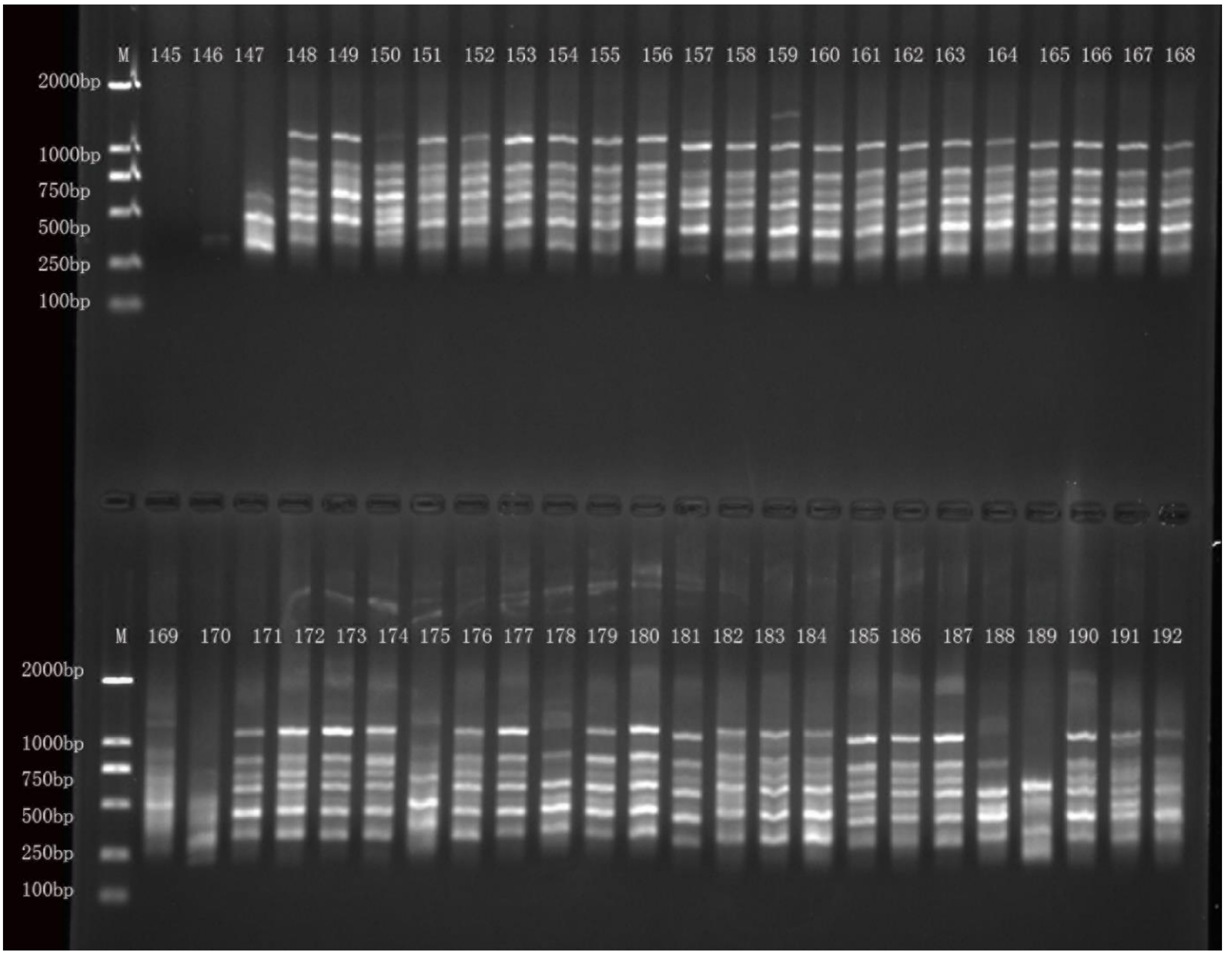

75. Amplification effect of primer ScoT 35 on *A.sinesis* samples (Voucher No. AS 145 - 192)

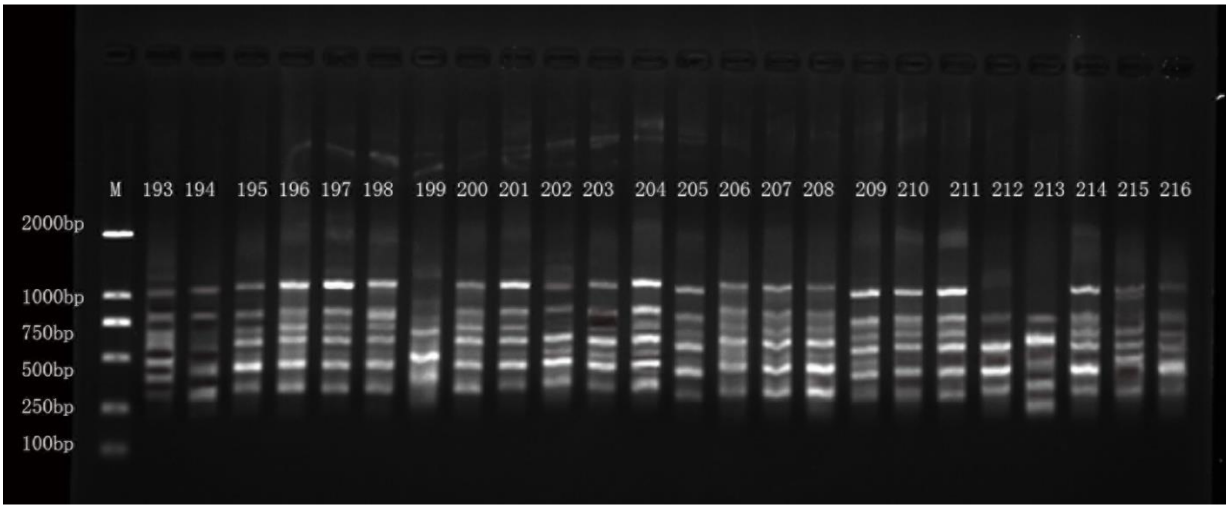

76. Amplification effect of primer ScoT 35 on *A.sinesis* samples (Voucher No. AS 193 - 216)

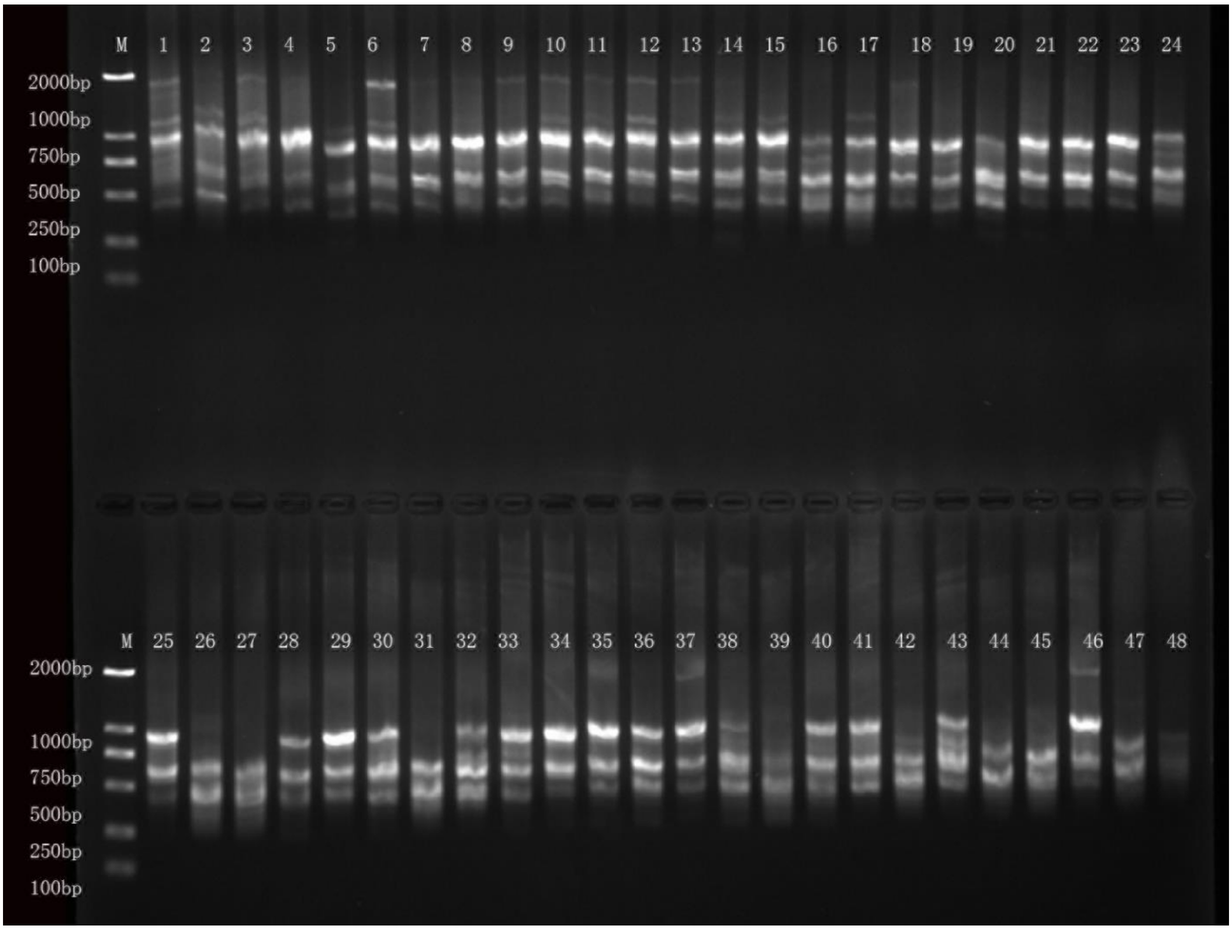

77. Amplification effect of primer ScoT 36 on *A.sinesis* samples (Voucher No. AS 1 - 48)

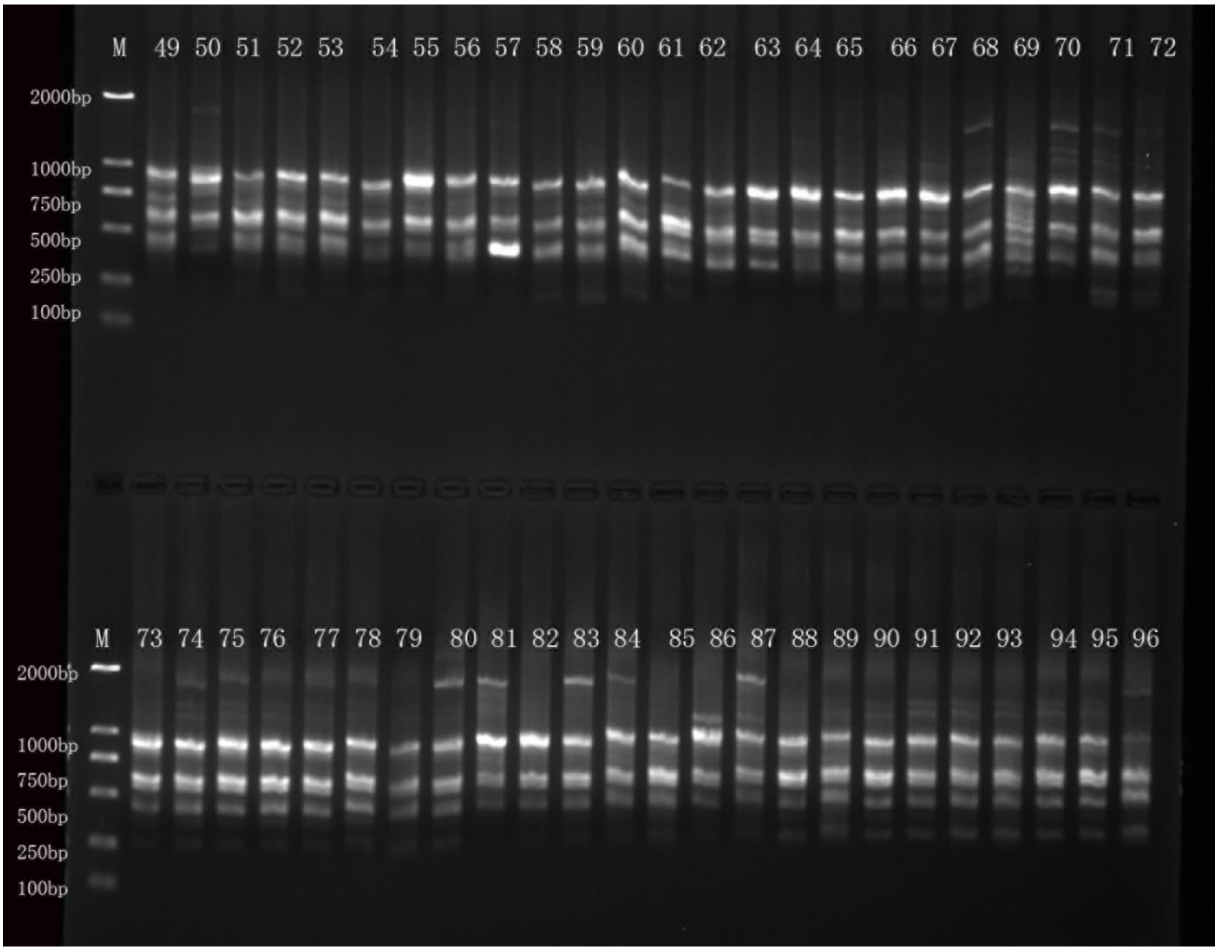

78. Amplification effect of primer ScoT 36 on *A.sinesis* samples (Voucher No. AS 49 - 96)

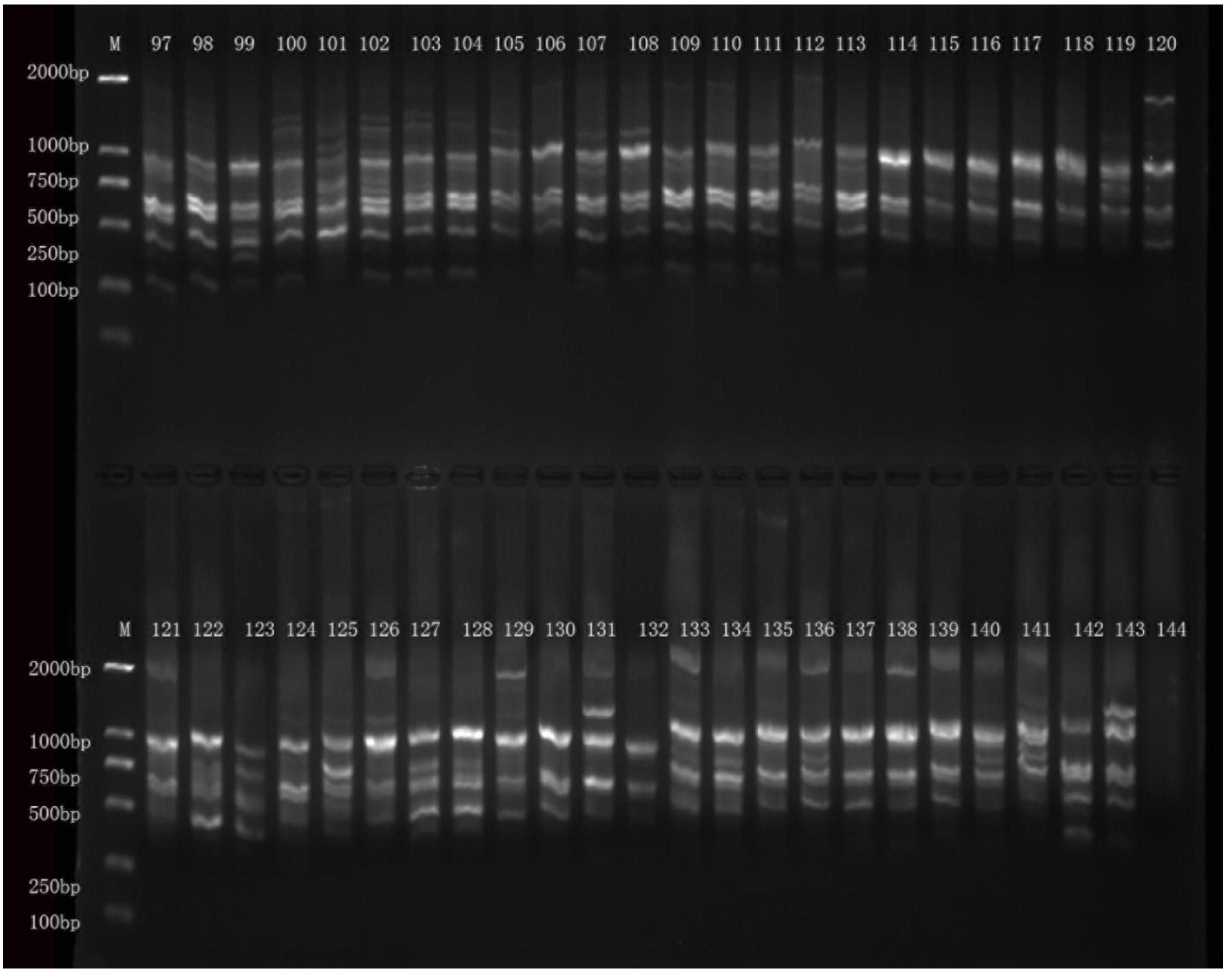

79. Amplification effect of primer ScoT 36 on *A.sinesis* samples (Voucher No. AS 97 - 144)

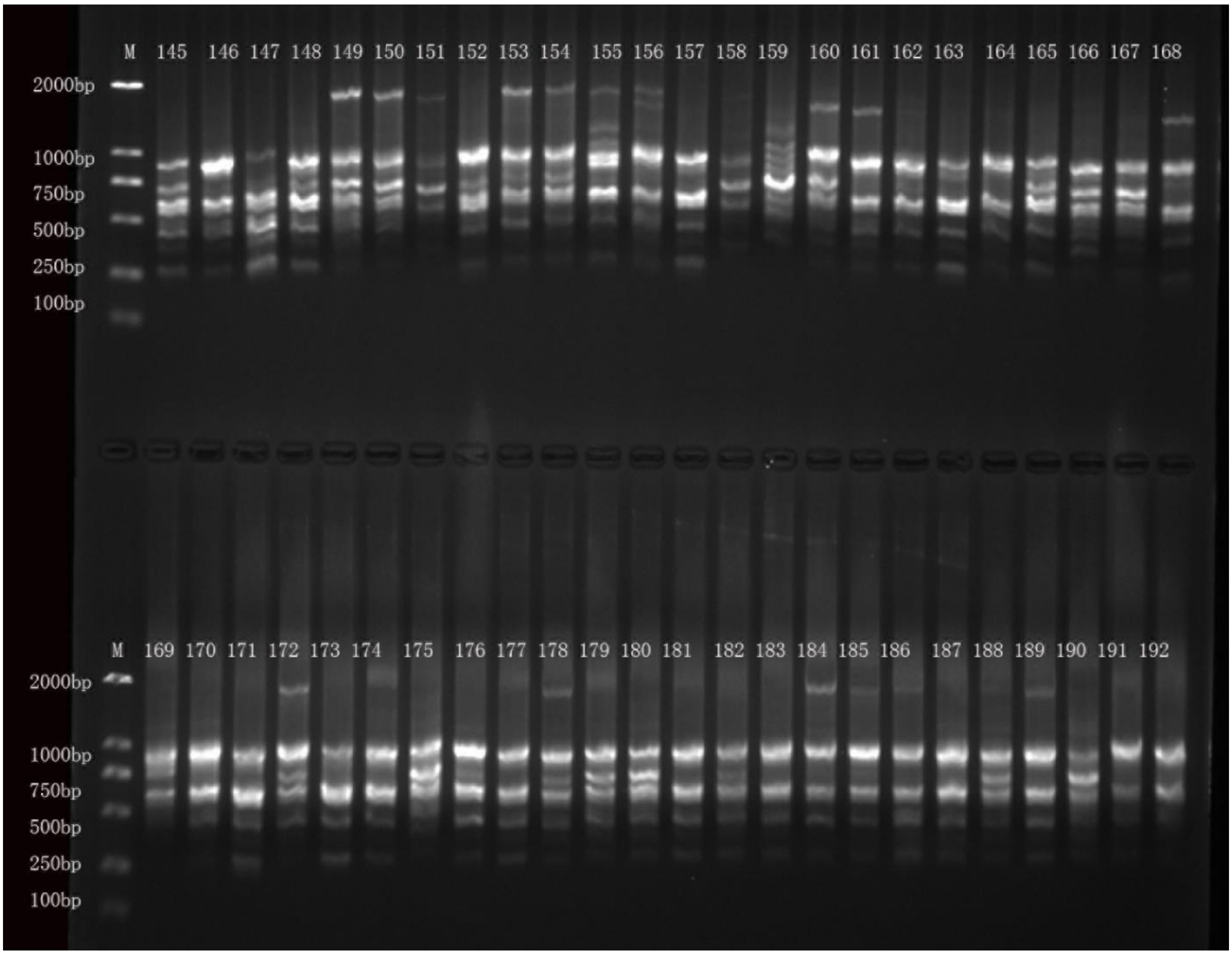

80. Amplification effect of primer ScoT 36 on *A.sinesis* samples (Voucher No. AS 145 - 192)

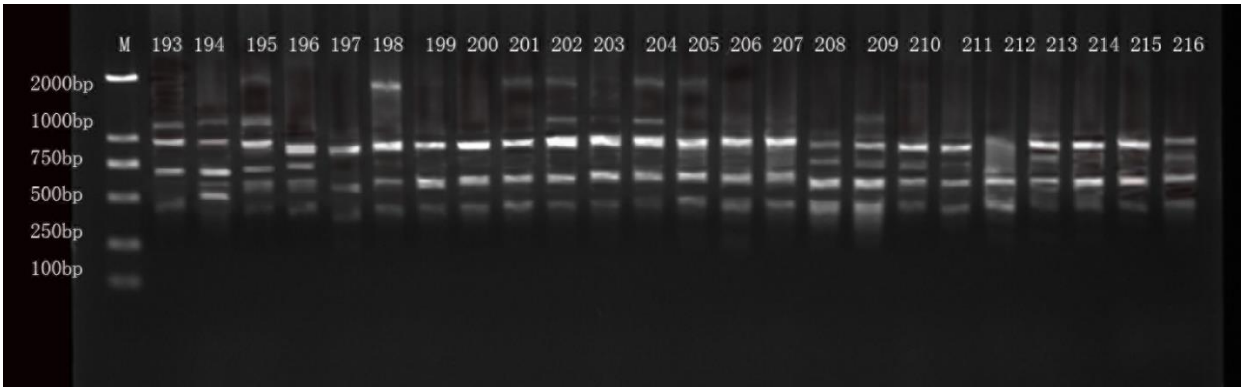

81. Amplification effect of primer ScoT 36 on A.sinesis samples (Voucher No. AS 193 - 216)

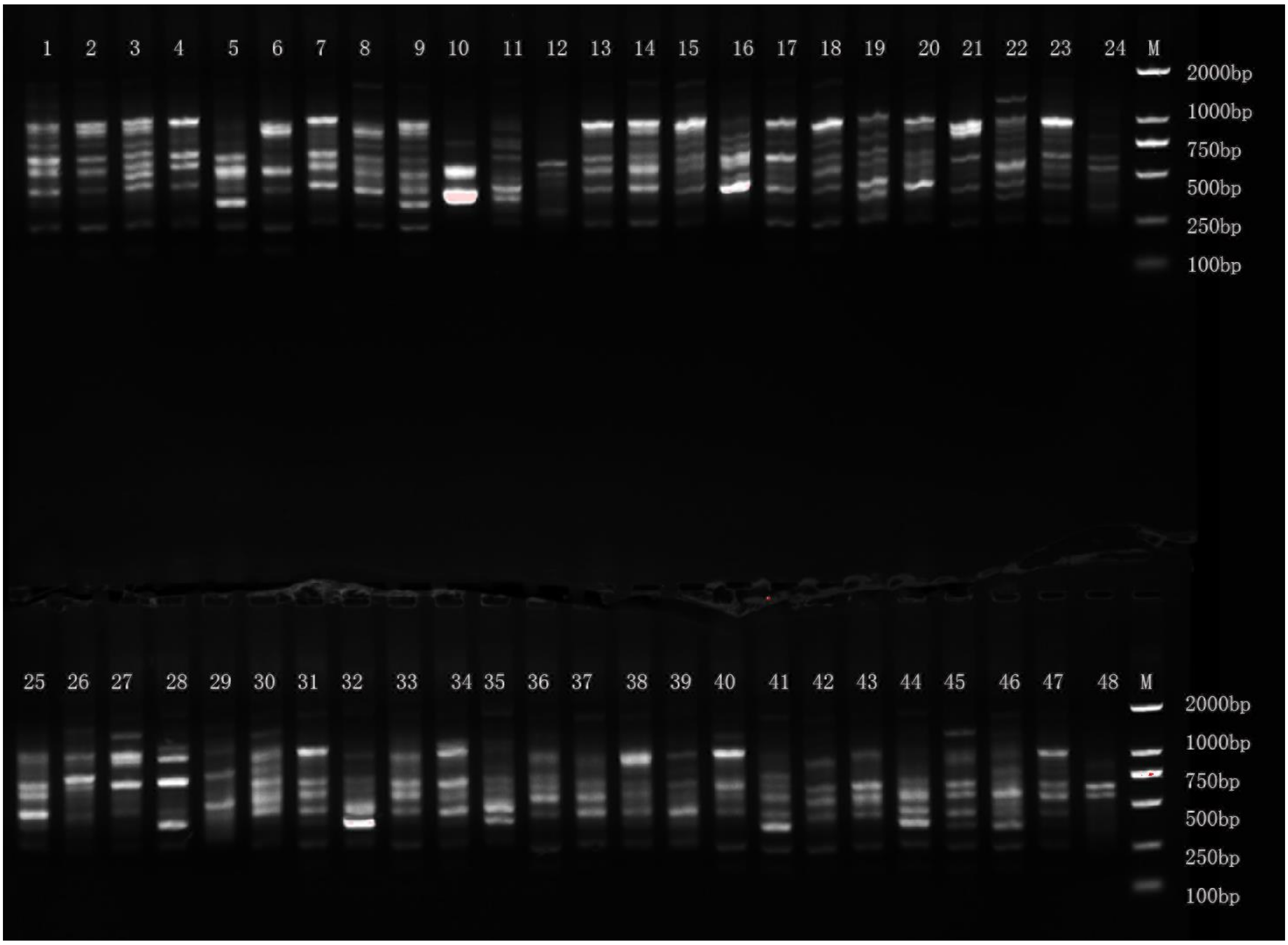

82. Amplification effect of primer ScoT 38 on *A.sinesis* samples (Voucher No. AS 1 - 48)

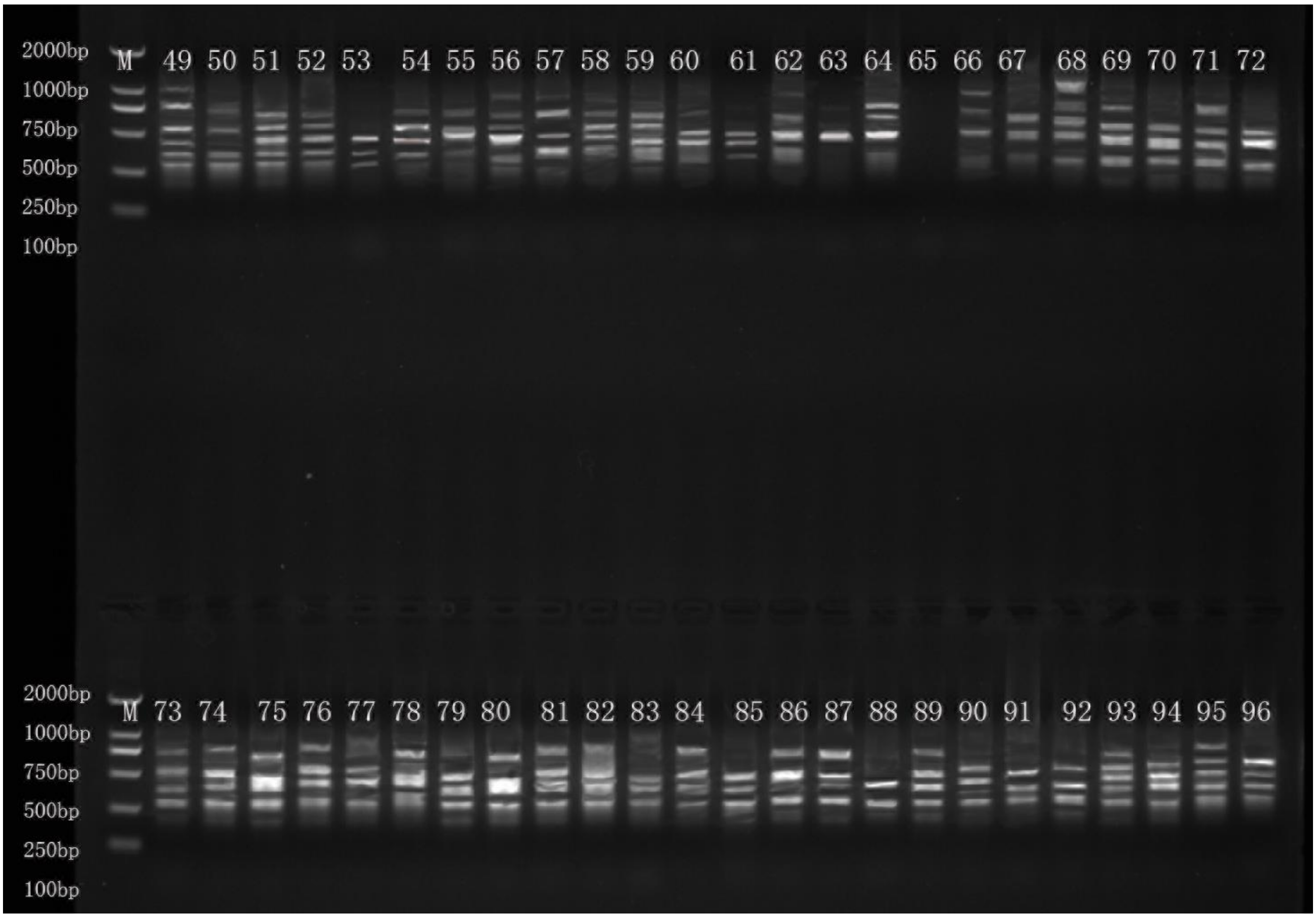

83. Amplification effect of primer ScoT 38 on *A.sinesis* samples (Voucher No. AS 49 - 96)

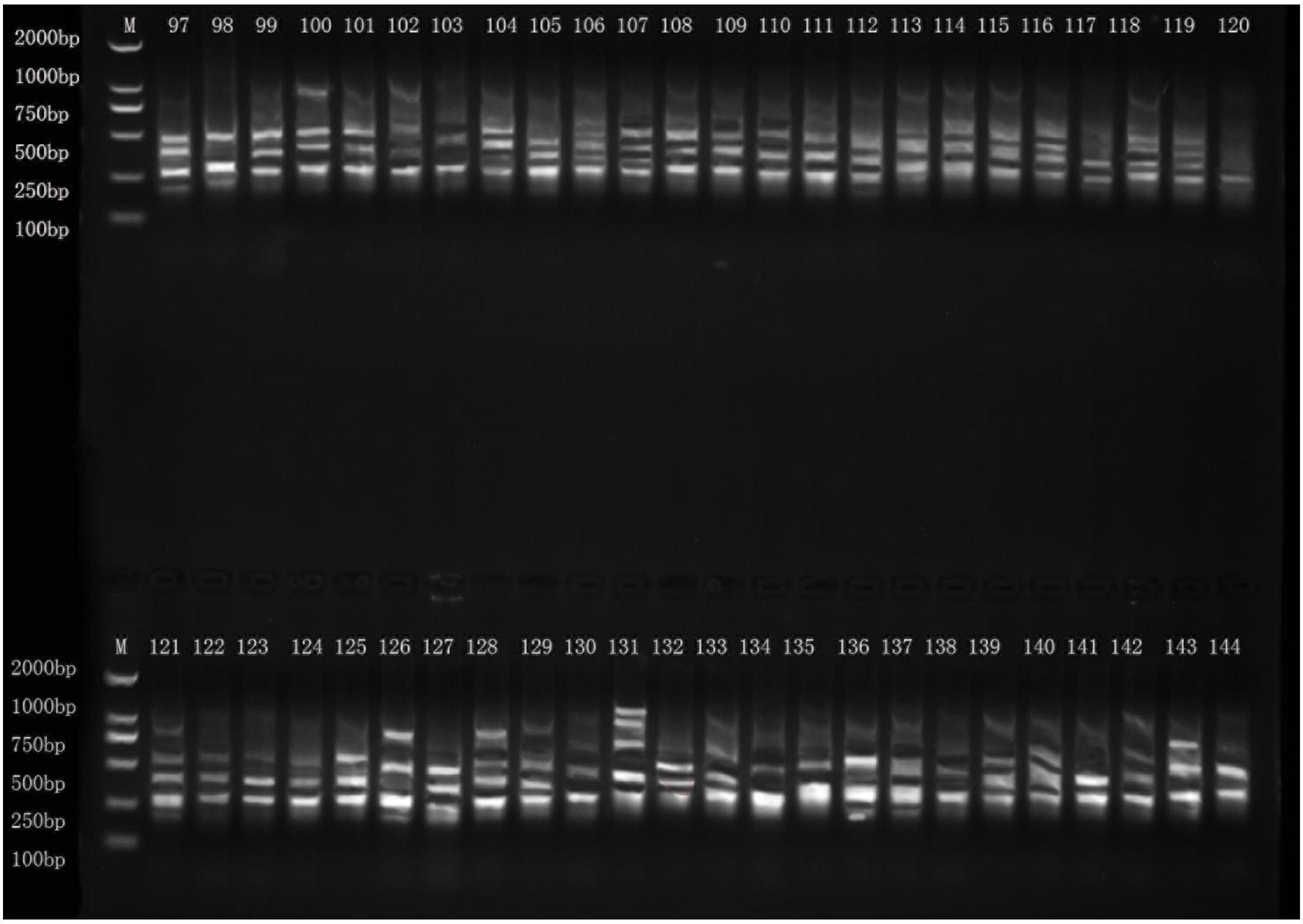

84. Amplification effect of primer ScoT 38 on *A.sinesis* samples (Voucher No. AS 97 - 144)

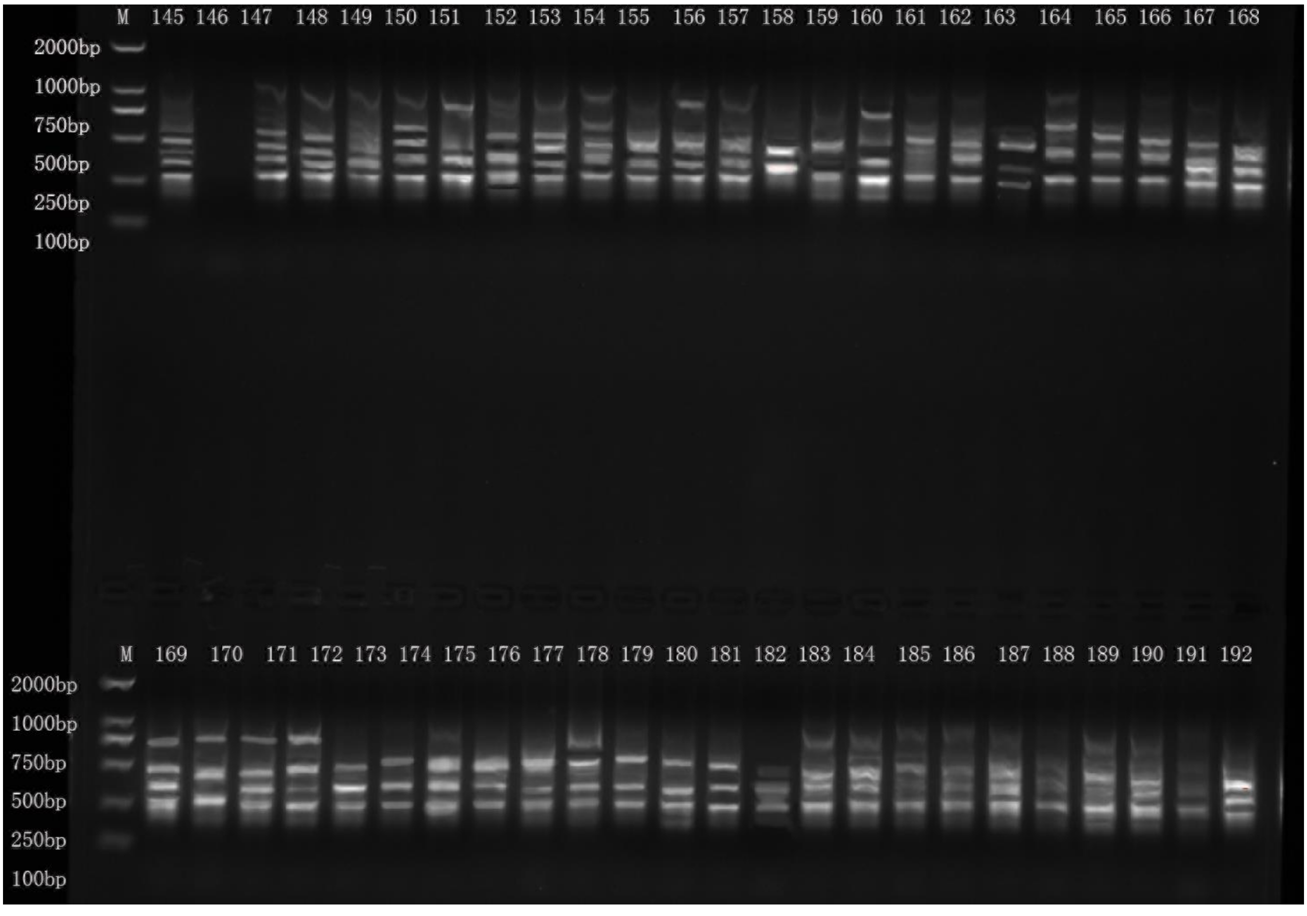

85. Amplification effect of primer ScoT 38 on *A.sinesis* samples (Voucher No. AS 145 - 192)

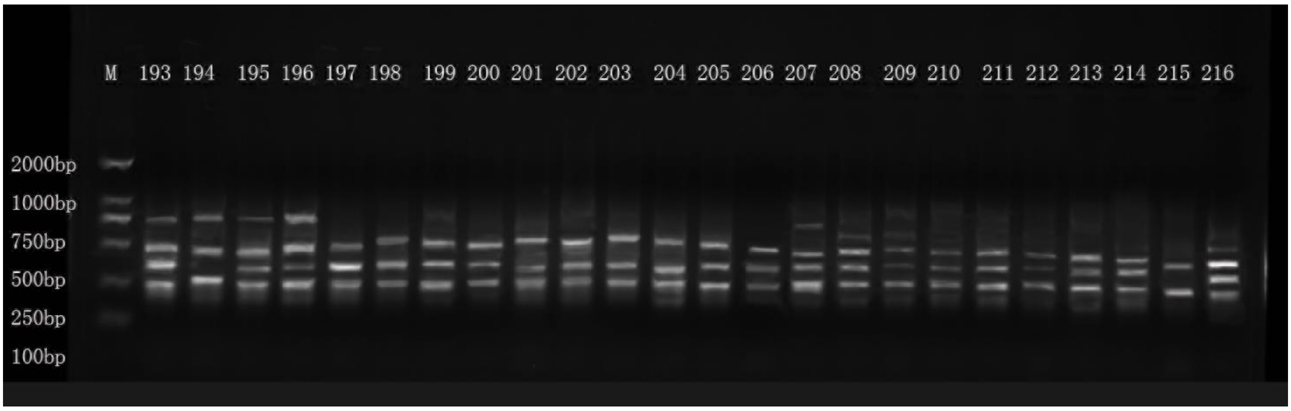

86. Amplification effect of primer ScoT 38 on *A.sinesis* samples (Voucher No. AS 193 - 216)

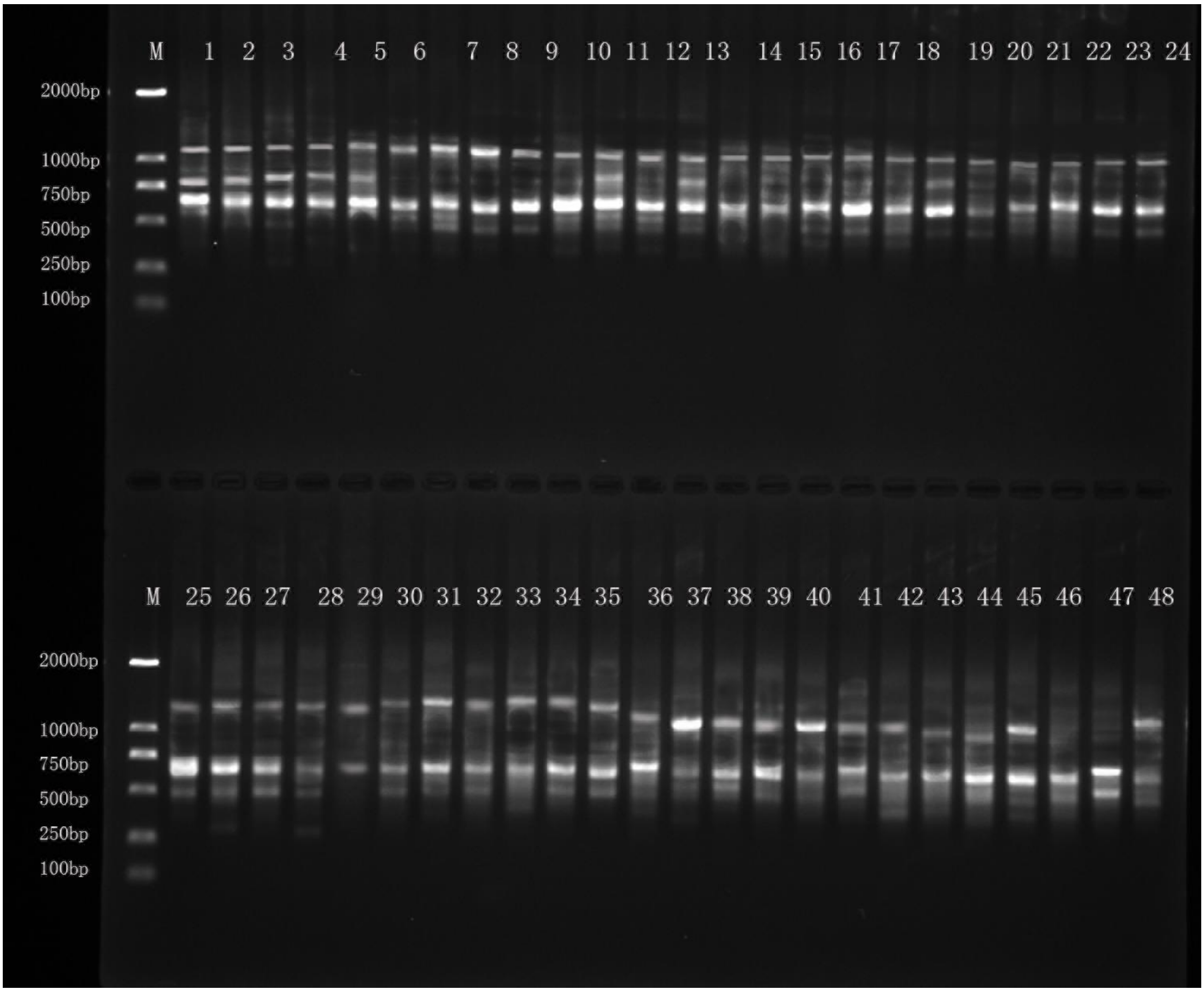

87. Amplification effect of primer ScoT 40 on *A.sinesis* samples (Voucher No. AS 1- 48)

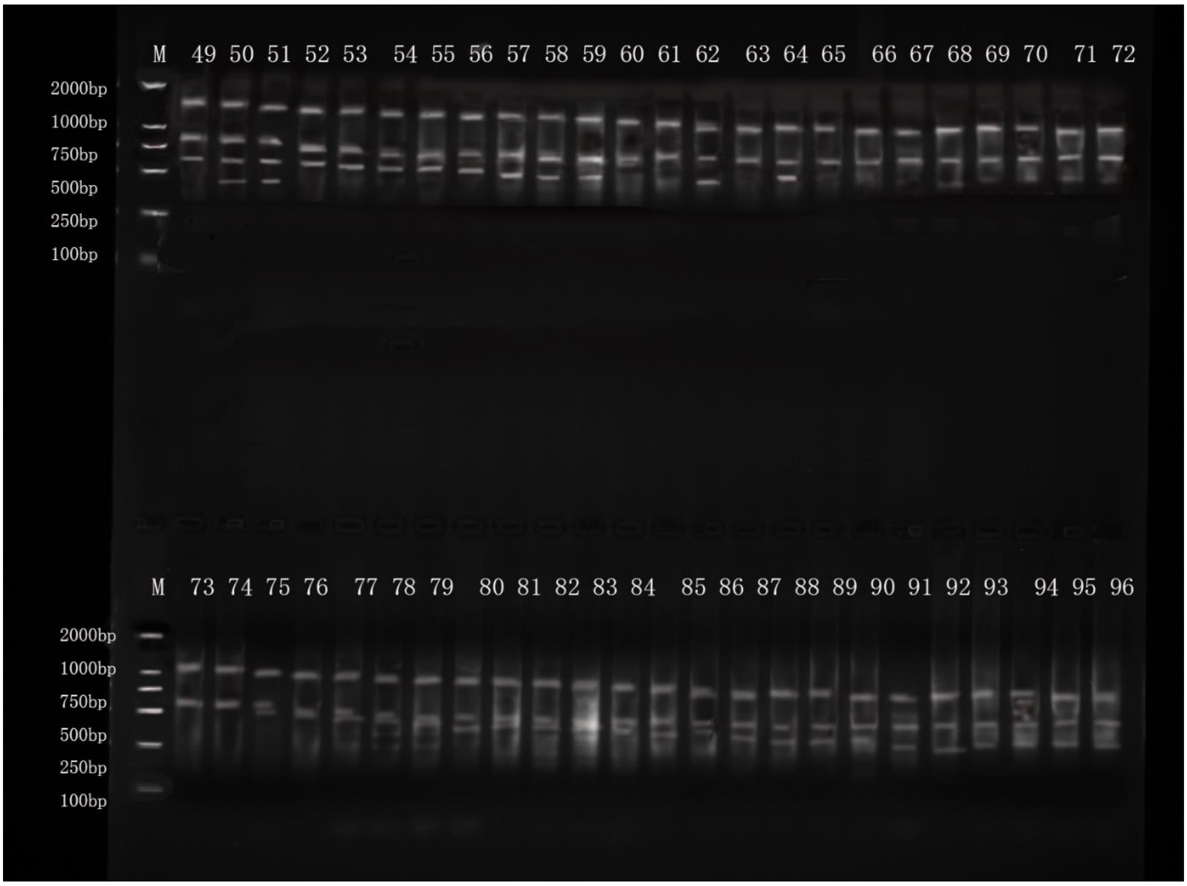

88. Amplification effect of primer ScoT 40 on *A.sinesis* samples (Voucher No. AS 49- 96)

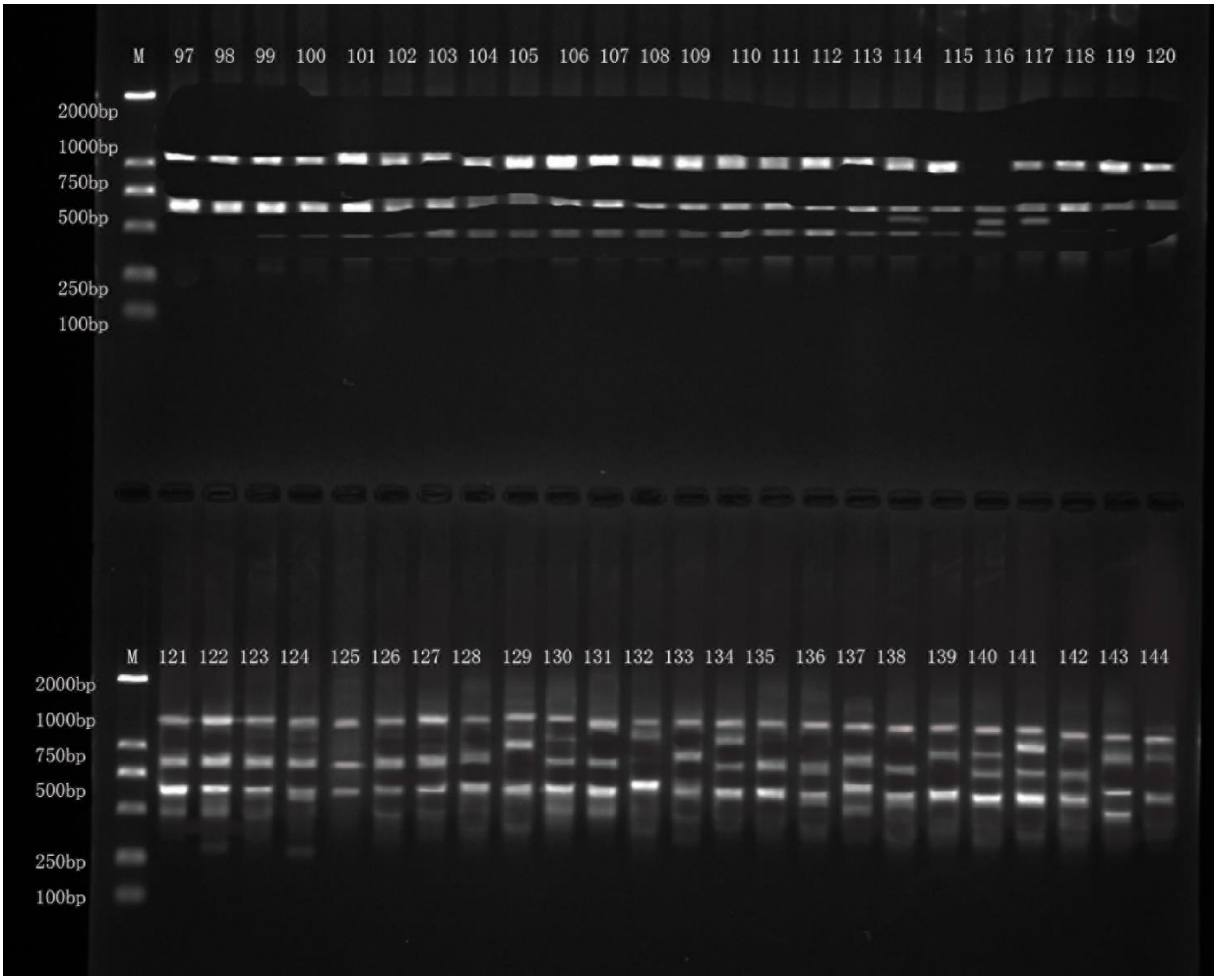

89. Amplification effect of primer ScoT 40 on *A.sinesis* samples (Voucher No. AS 97- 144)

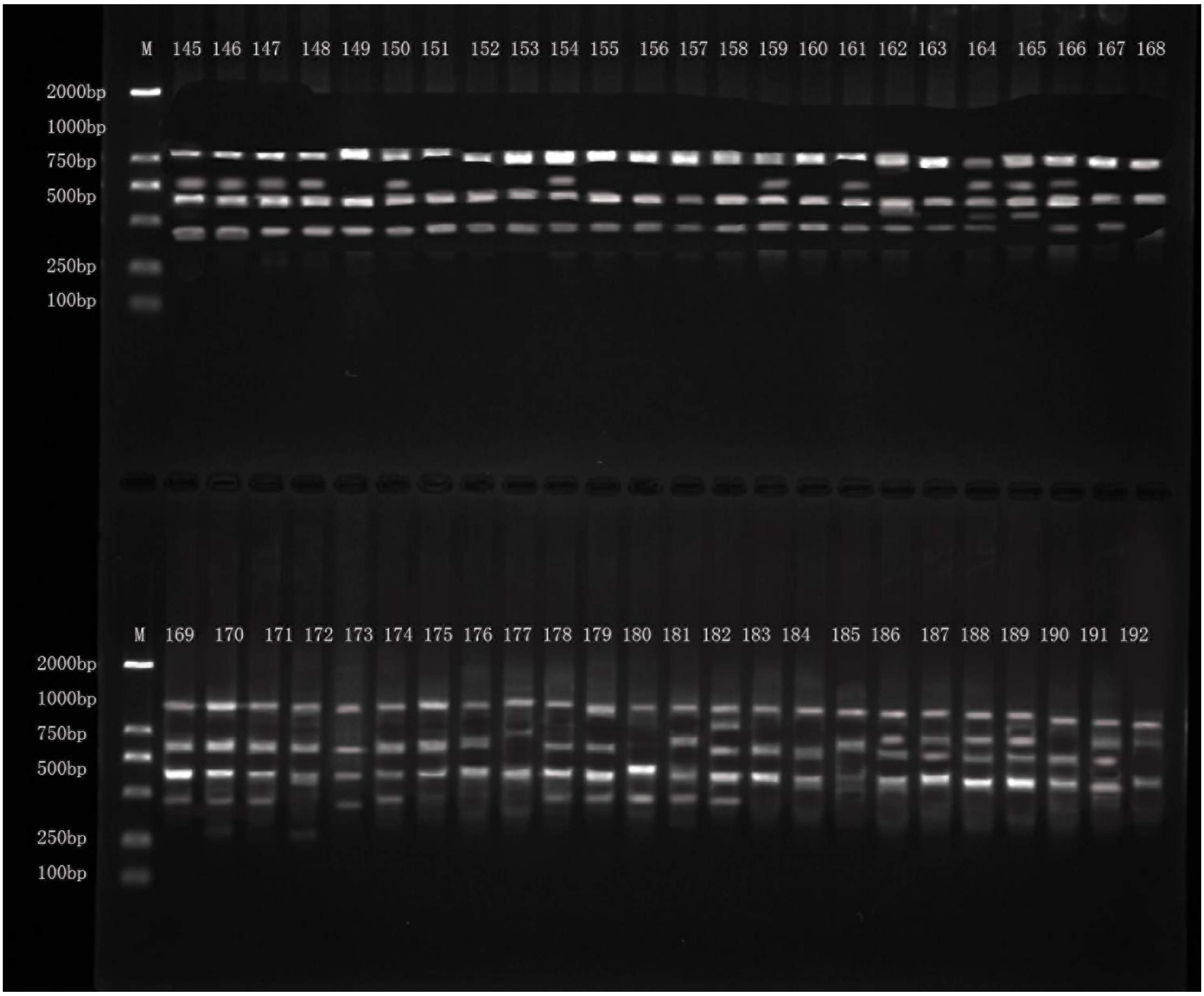

90. Amplification effect of primer ScoT 40 on *A.sinesis* samples (Voucher No. AS 145- 192)

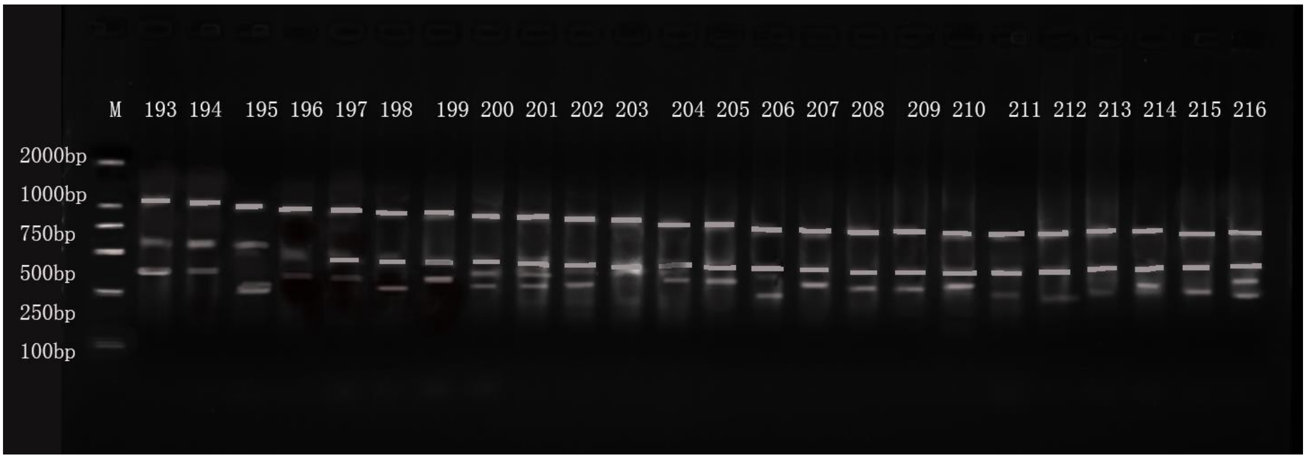

91. Amplification effect of primer ScoT 40 on *A.sinesis* samples (Voucher No. AS 193- 216)
