# Supplementary material for: Evaluation of genetic diversity and population structure of Annamocarya sinensis using SCoT markers
Source: PLoS One. 2024 Sep 4;19(9):e0309283. doi: 10.1371/journal.pone.0309283 (PMC11373820; doi:10.1371/journal.pone.0309283)
Supplement: S1 Table — (PDF) [file pone.0309283.s002.pdf]

S2 Table. Genetic Diversity Index of 20 primers

Primer	Observed Alleles （Na）	Effective Alleles （Ne）	Nei’s Genetic Diversity Index （H）	Shannon Information Index（I）
SCoT6	1.6666	1.1819	0.2400	0.3832
SCoT11	1.6000	1.3937	0.3351	0.4862
SCoT12	1.7500	1.4492	0.3385	0.5008
SCoT14	1.8000	1.4665	0.3398	0.5058
SCoT17	1.8000	1.4025	0.3135	0.4758
SCoT18	1.8334	1.4956	0.3438	0.5115
SCoT19	1.6364	1.3489	0.3148	0.4665
SCoT20	1.6628	1.1894	0.2417	0.3838
SCoT21	1.6924	1.2757	0.2785	0.4297
SCoT23	1.8462	1.3827	0.3009	0.3009
SCoT24	1.7778	1.3970	0.3152	0.4771
SCoT31	1.8334	1.4732	0.3330	0.4991
SCoT32	1.8182	1.4284	0.3244	0.4906
SCoT34	1.8182	1.5502	0.3716	0.5434
SCoT35	1.7142	1.3274	0.2980	0.4540
SCoT36	1.6924	1.2123	0.2503	0.3973
SCoT38	1.6000	1.4267	0.3484	0.5010
SCoT40	1.5556	1.2137	0.2718	0.4127
SCoT43	1.8182	1.5331	0.3645	0.5352
SCoT46	1.8182	1.5470	0.3686	0.5396
Mean	1.7368	1.3927	0.3263	0.4888
