# Supplementary material for: Evaluation of genetic diversity and population structure of Annamocarya sinensis using SCoT markers
Source: PLoS One. 2024 Sep 4;19(9):e0309283. doi: 10.1371/journal.pone.0309283 (PMC11373820; doi:10.1371/journal.pone.0309283)
Supplement: S2 Table — (PDF) [file pone.0309283.s003.pdf]

S3 Table. Genetic distance and geographical distances between populations in Guangxi

population	LY	LE	TL	TE	ND	HJ	LC	JJ	YZ	DL	DA	SJ	LS	JX	LZ	NP	JI	XC
LY	****	44.45	43.45	104.6	135	194.2	219.4	154.6	194.6	77.42	126	321.2	354.8	354.6	355.1	137.5	150.1	240.42
LE	0.122	****	52.88	66.05	112.7	167.8	200.3	148.2	192.7	88.47	140.8	303.5	339.2	361.4	353.9	178.4	191.8	242.30
TL	0.242	0.191	****	118.4	163.7	217.6	246	192.3	234.4	119.4	169.2	353.8	388.8	397.2	395.6	134.9	149.2	286.53
TE	0.146	0.102	0.181	****	63.59	118.1	159.7	116.5	162	98.61	141.2	248.3	285.5	334.4	316.6	241.9	254.7	197.50
ND	0.29	0.271	0.413	0.307	****	159.7	116.5	162	98.61	141.2	190.8	226.8	272.4	253	267.4	278.3	159.6	129.5
HJ	0.35	0.326	0.306	0.36	0.264	****	48.32	48.88	67.67	122.1	117.7	136.3	171.4	227.3	201.1	311.4	321	73.95
LC	0.198	0.168	0.18	0.149	0.26	0.241	****	54.26	31.57	135.7	111.4	120.5	149.1	179.2	156.9	322.9	330.8	35.95
JJ	0.284	0.26	0.237	0.249	0.298	0.306	0.235	****	45.86	82.11	68.96	171.2	202.2	218.3	205.8	271	279.6	81.18
YZ	0.239	0.242	0.311	0.238	0.328	0.382	0.251	0.158	****	118.2	85.08	148	173.4	172.5	161.5	300.3	307.3	35.15
DL	0.244	0.222	0.096	0.196	0.365	0.275	0.138	0.203	0.313	****	52.34	253.1	284.3	277.9	277.8	189.7	199	155.8
DA	0.23	0.27	0.235	0.236	0.338	0.296	0.237	0.184	0.199	0.197	****	231.7	258.4	229.2	235.2	216.1	222.6	103.81
SJ	0.325	0.278	0.247	0.277	0.302	0.176	0.241	0.312	0.374	0.219	0.301	****	38.4	187.5	131.2	442.2	450.6	93.88
LS	0.261	0.237	0.191	0.215	0.288	0.249	0.221	0.174	0.266	0.19	0.214	0.156	****	175	113.9	472	479.8	130.91
JX	0.123	0.151	0.238	0.191	0.271	0.282	0.165	0.234	0.257	0.202	0.233	0.23	0.215	****	62.45	430.3	433	147.2
LZ	0.177	0.2	0.294	0.225	0.324	0.371	0.231	0.266	0.309	0.255	0.323	0.284	0.26	0.127	****	447.8	452.3	149.71
NP	0.158	0.175	0.242	0.144	0.287	0.373	0.168	0.228	0.176	0.251	0.226	0.323	0.237	0.196	0.234	****	14.66	313.6
JI	0.193	0.174	0.296	0.158	0.31	0.443	0.254	0.239	0.191	0.292	0.222	0.367	0.266	0.243	0.287	0.12	****	313.1
XC	0.224	0.261	0.309	0.256	0.35	0.344	0.256	0.188	0.202	0.266	0.131	0.376	0.265	0.221	0.248	0.205	0.23	****

Note：Geographical distance in kilometers above diagonal and genetic distance below diagonal
