# Supplementary material for: Evaluation of genetic diversity and population structure of Annamocarya sinensis using SCoT markers
Source: PLoS One. 2024 Sep 4;19(9):e0309283. doi: 10.1371/journal.pone.0309283 (PMC11373820; doi:10.1371/journal.pone.0309283)
Supplement: S3 Table — (PDF) [file pone.0309283.s004.pdf]

S5 Table. Q value Compositions of 216 *A. sinensis* Accessions

No.	G.C	Q1	Q2	Q3	No.	G.C	Q1	Q2	Q3	No.	G.C	Q1	Q2	Q3
1	LY1	0.989	0.003	0.007	73	LC14	0.135	0.857	0.008	145	LS1	0.005	0.988	0.007
2	LY2	0.989	0.004	0.007	74	JJ1	0.088	0.514	0.397	146	LS2	0.006	0.983	0.011
3	LY3	0.974	0.009	0.017	75	JJ2	0.014	0.544	0.442	147	LS3	0.043	0.926	0.032
4	LY4	0.972	0.006	0.022	76	JJ3	0.015	0.546	0.438	148	LS4	0.007	0.979	0.013
5	LY5	0.975	0.011	0.014	77	JJ4	0.095	0.568	0.337	149	LS5	0.011	0.979	0.010
6	LY6	0.970	0.013	0.018	78	JJ5	0.027	0.635	0.338	150	LS6	0.005	0.984	0.011
7	LY7	0.967	0.018	0.016	79	JJ6	0.037	0.585	0.378	151	LS7	0.005	0.988	0.007
8	LY8	0.988	0.007	0.005	80	JJ7	0.032	0.591	0.377	152	LS8	0.062	0.749	0.189
9	LY9	0.982	0.006	0.012	81	JJ8	0.024	0.478	0.498	153	LS9	0.024	0.939	0.036
10	LY10	0.980	0.008	0.012	82	JJ9	0.018	0.481	0.501	154	LS10	0.017	0.858	0.125
11	LY11	0.977	0.007	0.016	83	JJ10	0.012	0.530	0.458	155	LS11	0.028	0.844	0.128
12	LY12	0.965	0.016	0.020	84	JJ11	0.015	0.613	0.372	156	LS12	0.011	0.870	0.119
13	LY13	0.983	0.007	0.010	85	JJ12	0.008	0.543	0.448	157	LS13	0.017	0.865	0.119
14	LE1	0.922	0.072	0.006	86	JJ13	0.082	0.533	0.385	158	LS14	0.008	0.964	0.028
15	LE2	0.944	0.048	0.007	87	JJ14	0.013	0.452	0.535	159	LS15	0.096	0.722	0.182
16	LE3	0.986	0.010	0.004	88	JJ15	0.006	0.451	0.542	160	LS16	0.011	0.741	0.248
17	LE4	0.973	0.021	0.006	89	JJ16	0.037	0.354	0.608	161	JX1	0.509	0.480	0.011
18	LE5	0.807	0.184	0.009	90	JJ17	0.020	0.396	0.584	162	JX2	0.676	0.314	0.010
19	LE6	0.959	0.036	0.005	91	JJ18	0.008	0.490	0.502	163	JX3	0.732	0.263	0.005
20	LE7	0.983	0.013	0.004	92	YZ1	0.365	0.129	0.506	164	JX4	0.735	0.252	0.013
21	LE8	0.952	0.038	0.010	93	YZ2	0.444	0.032	0.524	165	JX5	0.741	0.234	0.025
22	LE9	0.954	0.036	0.011	94	YZ3	0.597	0.054	0.349	166	JX6	0.577	0.334	0.089
23	LE10	0.973	0.017	0.009	95	YZ4	0.501	0.012	0.487	167	JX7	0.754	0.226	0.020
24	LE11	0.985	0.011	0.004	96	YZ5	0.484	0.026	0.490	168	JX8	0.598	0.375	0.026
25	LE12	0.980	0.008	0.011	97	YZ6	0.515	0.012	0.474	169	JX9	0.572	0.423	0.005
26	LE13	0.979	0.015	0.006	98	YZ7	0.519	0.007	0.474	170	JX10	0.655	0.277	0.068
27	LE14	0.989	0.007	0.004	99	DL1	0.064	0.904	0.032	171	JX11	0.498	0.491	0.010
28	TL1	0.365	0.579	0.056	100	DL2	0.030	0.905	0.065	172	JX12	0.606	0.385	0.009

29	TL2	0.314	0.650	0.036	101	DL3	0.033	0.894	0.073	173	JX13	0.632	0.330	0.038
30	TL3	0.306	0.652	0.042	102	DL4	0.047	0.907	0.046	174	JX14	0.580	0.398	0.022
31	TL4	0.349	0.632	0.020	103	DL5	0.014	0.825	0.161	175	JX15	0.739	0.253	0.008
32	TL5	0.244	0.711	0.045	104	DL6	0.043	0.751	0.207	176	JX16	0.692	0.301	0.007
33	TL6	0.394	0.593	0.013	105	DL7	0.026	0.721	0.253	177	JX17	0.694	0.288	0.018
34	TL7	0.149	0.781	0.069	106	DL8	0.013	0.809	0.179	178	LZ1	0.725	0.260	0.016
35	TL8	0.323	0.627	0.049	107	DL9	0.065	0.739	0.196	179	LZ2	0.898	0.097	0.005
36	TL9	0.349	0.622	0.029	108	DA1	0.003	0.003	0.994	180	LZ3	0.879	0.114	0.006
37	TE1	0.967	0.018	0.015	109	DA2	0.004	0.003	0.993	181	LZ4	0.690	0.304	0.005
38	TE2	0.974	0.016	0.009	110	DA3	0.003	0.003	0.994	182	LZ5	0.814	0.178	0.008
39	TE3	0.890	0.012	0.098	111	DA4	0.008	0.007	0.985	183	LZ6	0.939	0.049	0.012
40	TE4	0.924	0.021	0.055	112	DA5	0.003	0.004	0.993	184	LZ7	0.883	0.101	0.016
41	TE5	0.969	0.013	0.018	113	DA6	0.011	0.010	0.979	185	LZ8	0.537	0.453	0.010
42	TE6	0.950	0.018	0.033	114	DA7	0.004	0.004	0.992	186	LZ9	0.924	0.070	0.007
43	ND1	0.363	0.616	0.020	115	DA8	0.004	0.005	0.991	187	LZ10	0.700	0.287	0.013
44	ND2	0.366	0.620	0.014	116	DA9	0.003	0.003	0.994	188	NP1	0.975	0.007	0.018
45	ND3	0.340	0.648	0.012	117	DA10	0.007	0.007	0.987	189	NP2	0.858	0.031	0.111
46	ND4	0.461	0.525	0.014	118	DA11	0.003	0.004	0.993	190	NP3	0.959	0.005	0.036
47	ND5	0.442	0.551	0.008	119	DA12	0.012	0.011	0.977	191	NP4	0.891	0.006	0.103
48	ND6	0.339	0.649	0.011	120	DA13	0.004	0.004	0.992	192	NP5	0.988	0.005	0.007
49	HJ1	0.005	0.980	0.015	121	DA14	0.004	0.006	0.990	193	NP6	0.966	0.007	0.027
50	HJ2	0.004	0.990	0.006	122	DA15	0.004	0.003	0.993	194	NP7	0.976	0.012	0.012
51	HJ3	0.005	0.990	0.006	123	DA16	0.003	0.004	0.993	195	NP8	0.957	0.033	0.009
52	HJ4	0.009	0.974	0.018	124	DA17	0.004	0.005	0.992	196	NP9	0.961	0.008	0.031
53	HJ5	0.006	0.983	0.011	125	DA18	0.004	0.005	0.991	197	NP10	0.925	0.010	0.064
54	HJ6	0.007	0.988	0.005	126	DA19	0.003	0.005	0.992	198	NP11	0.771	0.012	0.217
55	HJ7	0.005	0.989	0.007	127	DA20	0.004	0.045	0.951	199	NP12	0.853	0.091	0.056
56	HJ8	0.004	0.990	0.007	128	DA21	0.008	0.033	0.960	200	NP13	0.897	0.061	0.042
57	HJ9	0.004	0.991	0.005	129	DA22	0.017	0.053	0.930	201	NP14	0.957	0.028	0.014
58	HJ10	0.004	0.989	0.007	130	DA23	0.013	0.026	0.961	202	NP15	0.943	0.007	0.051

59	HJ11	0.007	0.988	0.004	131	DA24	0.053	0.087	0.860	203	NP16	0.945	0.020	0.035
60	LC1	0.513	0.477	0.010	132	DA25	0.017	0.019	0.964	204	NP17	0.919	0.034	0.046
61	LC2	0.458	0.534	0.009	133	DA26	0.009	0.015	0.976	205	NP18	0.974	0.009	0.017
62	LC3	0.526	0.466	0.008	134	DA27	0.006	0.007	0.987	206	JI1	0.911	0.006	0.083
63	LC4	0.363	0.632	0.005	135	DA28	0.007	0.014	0.979	207	JI2	0.734	0.016	0.250
64	LC5	0.402	0.465	0.133	136	SJ1	0.016	0.957	0.028	208	JI3	0.898	0.005	0.097
65	LC6	0.306	0.640	0.054	137	SJ2	0.011	0.976	0.013	209	JI4	0.820	0.009	0.170
66	LC7	0.568	0.421	0.012	138	SJ3	0.014	0.982	0.005	210	JI5	0.817	0.007	0.177
67	LC8	0.327	0.659	0.015	139	SJ4	0.009	0.984	0.006	211	JI6	0.764	0.008	0.228
68	LC9	0.193	0.798	0.009	140	SJ5	0.012	0.983	0.004	212	XC1	0.318	0.086	0.596
69	LC10	0.238	0.752	0.010	141	SJ6	0.005	0.990	0.004	213	XC2	0.366	0.050	0.584
70	LC11	0.047	0.943	0.009	142	SJ7	0.010	0.981	0.008	214	XC3	0.389	0.019	0.591
71	LC12	0.311	0.681	0.008	143	SJ8	0.006	0.988	0.006	215	XC4	0.470	0.033	0.497
72	LC13	0.304	0.686	0.011	144	SJ9	0.011	0.984	0.005	216	XC5	0.351	0.087	0.562

Note: G.C, Genotype Code
