# Supplementary material for: Evaluation of genetic diversity and population structure of Annamocarya sinensis using SCoT markers
Source: PLoS One. 2024 Sep 4;19(9):e0309283. doi: 10.1371/journal.pone.0309283 (PMC11373820; doi:10.1371/journal.pone.0309283)
Supplement: S1 Fig — (PDF) [file pone.0309283.s005.pdf]

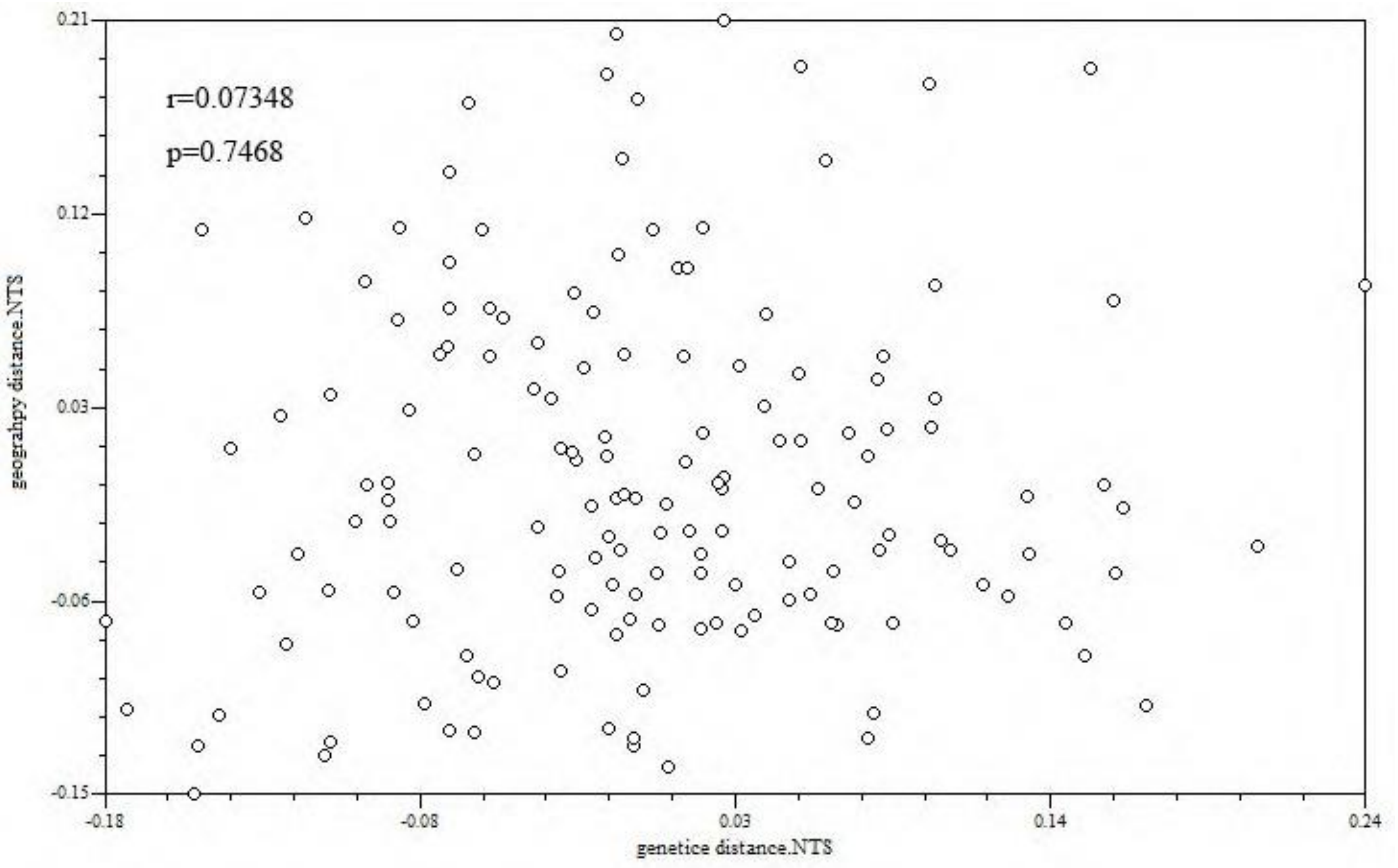

S4 Figure. **Correlation Analysis of Mantel Test between Genetic Distance and Geographical Distance**
