# Supplementary material for: Evaluation of genetic diversity and population structure of Annamocarya sinensis using SCoT markers
Source: PLoS One. 2024 Sep 4;19(9):e0309283. doi: 10.1371/journal.pone.0309283 (PMC11373820; doi:10.1371/journal.pone.0309283)
Supplement: S1 Dataset — (ZIP) [file pone.0309283.s006.zip › Minimal Data Set/Guide to the Minimal Data Set Utilization.docx]

Guide to the Minimal Data Set Utilization

1. The presence （ “1”）or absence （“0”）of the bands created by the SCoT-PCR amplification were counted to establish the file “ SCoT 01 basic data” (a binary data matrix). NTsys2.10 was used to calculate Genetic Similarity Coefficients, FS, TNB, PNB, PPB et al. and was used to performed UPGMA clustering Analysis, the result of clustering analysis (the file “MEGA basic data.meg”) was imported into MEGA 7 to build the Neighbor-Joining cluster diagram (Figure 1 of the manuscript).
2. Cervus 3.0 was used to calculate PIC and Rp values based on the data of “SCoT 01 basic data” [1].
3. The presence （ “1” ）or absence （ “0” ）of the bands created by the SCoT-PCR amplification were counted to establish the file “Popgen basic data” (a binary data matrix). The values of PPB, Na, Ne, I and H of 18 populations (Table 3 of the manuscript) were calculated by Popgene32 (V 1.32) [2]to obtain the file “Popgen Result.rst”.
4. Based on the file “SCoT 01 basic data”, NTsys2.10 was used to calculate the results of Genetic Distances (the file “genetic distance.NTS”). the data of “genetic distance.NTS” were then uploaded to the website ([https://www.chiplot.online/pcoa.html) to](https://www.chiplot.online/pcoa.html)%20to) perform PCoA analysis (Figure 2 of the manuscript).
5. The Mantel test of correlation between Geographical Distances (the file “geograhpy distance.NTS”) and Genetic Distances were also performed by NTsys pc2.10.
6. The presence （ “1” ）or absence （ “0” ）of the bands created by the SCoT-PCR amplification were counted to establish the file “STRUCTURE basic data” (a binary data matrix). STRUCTURE2.3.4 was used to perform population structure analysis, the results were then uploaded to the website (http://taylor0. biology.ucla.edu/struct_harvest) to obtain the population structure diagram and Q value compositions (Figure 3 and S5 Table of the manuscript)
7. The presence （ “1” ）or absence （ “0” ）of the bands created by the SCoT-PCR amplification were counted to establish the file “GENALEX basic data” (a binary data matrix). The AMOMA analysis were then performed by GenAIEx6.51b2 and Popgene 32 (V 1.32) [3].

[1]Kalinowski ST, Taper ML, Marshall TC. Revising how the computer program CERVUS accommodates genotyping error increases success in paternity assignment[J]. Molecular ecology. 2007; 16: 1099-1106.)

[2] Francis C.Yeh and Rong-cai Yang. Popgene version 1.31 Quick User Guide[CP/OL]. University of Alberta and Tim Boyle,Centre for International Forestry Research,1999.

[3]Michaela D.J. Blyton and Nicola S. Flanagan. A Comprehensive Guide to:GenAIEx 6.5[CP/OL]. Australian National University, 2012.http://biology.anu.edu.au/GenAlEx/
